# Search for new phenomena in events containing a same-flavour opposite-sign dilepton pair, jets, and large missing transverse momentum in $$\varvec{\sqrt{s}=13}$$ $$\text {TeV}$$$$\varvec{pp}$$ collisions with the ATLAS detector

**DOI:** 10.1140/epjc/s10052-017-4700-5

**Published:** 2017-03-04

**Authors:** M. Aaboud, G. Aad, B. Abbott, J. Abdallah, O. Abdinov, B. Abeloos, O. S. AbouZeid, N. L. Abraham, H. Abramowicz, H. Abreu, R. Abreu, Y. Abulaiti, B. S. Acharya, S. Adachi, L. Adamczyk, D. L. Adams, J. Adelman, T. Adye, A. A. Affolder, T. Agatonovic-Jovin, C. Agheorghiesei, J. A. Aguilar-Saavedra, S. P. Ahlen, F. Ahmadov, G. Aielli, H. Akerstedt, T. P. A. Åkesson, A. V. Akimov, G. L. Alberghi, J. Albert, M. J. Alconada Verzini, M. Aleksa, I. N. Aleksandrov, C. Alexa, G. Alexander, T. Alexopoulos, M. Alhroob, B. Ali, M. Aliev, G. Alimonti, J. Alison, S. P. Alkire, B. M. M. Allbrooke, B. W. Allen, P. P. Allport, A. Aloisio, A. Alonso, F. Alonso, C. Alpigiani, A. A. Alshehri, M. Alstaty, B. Alvarez Gonzalez, D. Álvarez Piqueras, M. G. Alviggi, B. T. Amadio, Y. Amaral Coutinho, C. Amelung, D. Amidei, S. P. Amor Dos Santos, A. Amorim, S. Amoroso, G. Amundsen, C. Anastopoulos, L. S. Ancu, N. Andari, T. Andeen, C. F. Anders, J. K. Anders, K. J. Anderson, A. Andreazza, V. Andrei, S. Angelidakis, I. Angelozzi, A. Angerami, F. Anghinolfi, A. V. Anisenkov, N. Anjos, A. Annovi, C. Antel, M. Antonelli, A. Antonov, D. J. Antrim, F. Anulli, M. Aoki, L. Aperio Bella, G. Arabidze, Y. Arai, J. P. Araque, V. Araujo Ferraz, A. T. H. Arce, F. A. Arduh, J.-F. Arguin, S. Argyropoulos, M. Arik, A. J. Armbruster, L. J. Armitage, O. Arnaez, H. Arnold, M. Arratia, O. Arslan, A. Artamonov, G. Artoni, S. Artz, S. Asai, N. Asbah, A. Ashkenazi, B. Åsman, L. Asquith, K. Assamagan, R. Astalos, M. Atkinson, N. B. Atlay, K. Augsten, G. Avolio, B. Axen, M. K. Ayoub, G. Azuelos, M. A. Baak, A. E. Baas, M. J. Baca, H. Bachacou, K. Bachas, M. Backes, M. Backhaus, P. Bagiacchi, P. Bagnaia, Y. Bai, J. T. Baines, M. Bajic, O. K. Baker, E. M. Baldin, P. Balek, T. Balestri, F. Balli, W. K. Balunas, E. Banas, Sw. Banerjee, A. A. E. Bannoura, L. Barak, E. L. Barberio, D. Barberis, M. Barbero, T. Barillari, M-S Barisits, T. Barklow, N. Barlow, S. L. Barnes, B. M. Barnett, R. M. Barnett, Z. Barnovska-Blenessy, A. Baroncelli, G. Barone, A. J. Barr, L. Barranco Navarro, F. Barreiro, J. Barreiro Guimarães da Costa, R. Bartoldus, A. E. Barton, P. Bartos, A. Basalaev, A. Bassalat, R. L. Bates, S. J. Batista, J. R. Batley, M. Battaglia, M. Bauce, F. Bauer, H. S. Bawa, J. B. Beacham, M. D. Beattie, T. Beau, P. H. Beauchemin, P. Bechtle, H. P. Beck, K. Becker, M. Becker, M. Beckingham, C. Becot, A. J. Beddall, A. Beddall, V. A. Bednyakov, M. Bedognetti, C. P. Bee, L. J. Beemster, T. A. Beermann, M. Begel, J. K. Behr, A. S. Bell, G. Bella, L. Bellagamba, A. Bellerive, M. Bellomo, K. Belotskiy, O. Beltramello, N. L. Belyaev, O. Benary, D. Benchekroun, M. Bender, K. Bendtz, N. Benekos, Y. Benhammou, E. Benhar Noccioli, J. Benitez, D. P. Benjamin, J. R. Bensinger, S. Bentvelsen, L. Beresford, M. Beretta, D. Berge, E. Bergeaas Kuutmann, N. Berger, J. Beringer, S. Berlendis, N. R. Bernard, C. Bernius, F. U. Bernlochner, T. Berry, P. Berta, C. Bertella, G. Bertoli, F. Bertolucci, I. A. Bertram, C. Bertsche, D. Bertsche, G. J. Besjes, O. Bessidskaia Bylund, M. Bessner, N. Besson, C. Betancourt, A. Bethani, S. Bethke, A. J. Bevan, R. M. Bianchi, M. Bianco, O. Biebel, D. Biedermann, R. Bielski, N. V. Biesuz, M. Biglietti, J. Bilbao De Mendizabal, T. R. V. Billoud, H. Bilokon, M. Bindi, A. Bingul, C. Bini, S. Biondi, T. Bisanz, D. M. Bjergaard, C. W. Black, J. E. Black, K. M. Black, D. Blackburn, R. E. Blair, T. Blazek, I. Bloch, C. Blocker, A. Blue, W. Blum, U. Blumenschein, S. Blunier, G. J. Bobbink, V. S. Bobrovnikov, S. S. Bocchetta, A. Bocci, C. Bock, M. Boehler, D. Boerner, J. A. Bogaerts, D. Bogavac, A. G. Bogdanchikov, C. Bohm, V. Boisvert, P. Bokan, T. Bold, A. S. Boldyrev, M. Bomben, M. Bona, M. Boonekamp, A. Borisov, G. Borissov, J. Bortfeldt, D. Bortoletto, V. Bortolotto, K. Bos, D. Boscherini, M. Bosman, J. D. Bossio Sola, J. Boudreau, J. Bouffard, E. V. Bouhova-Thacker, D. Boumediene, C. Bourdarios, S. K. Boutle, A. Boveia, J. Boyd, I. R. Boyko, J. Bracinik, A. Brandt, G. Brandt, O. Brandt, U. Bratzler, B. Brau, J. E. Brau, W. D. Breaden Madden, K. Brendlinger, A. J. Brennan, L. Brenner, R. Brenner, S. Bressler, T. M. Bristow, D. Britton, D. Britzger, F. M. Brochu, I. Brock, R. Brock, G. Brooijmans, T. Brooks, W. K. Brooks, J. Brosamer, E. Brost, J. H Broughton, P. A. Bruckman de Renstrom, D. Bruncko, A. Bruni, G. Bruni, L. S. Bruni, BH Brunt, M. Bruschi, N. Bruscino, P. Bryant, L. Bryngemark, T. Buanes, Q. Buat, P. Buchholz, A. G. Buckley, I. A. Budagov, F. Buehrer, M. K. Bugge, O. Bulekov, D. Bullock, H. Burckhart, S. Burdin, C. D. Burgard, A. M. Burger, B. Burghgrave, K. Burka, S. Burke, I. Burmeister, J. T. P. Burr, E. Busato, D. Büscher, V. Büscher, P. Bussey, J. M. Butler, C. M. Buttar, J. M. Butterworth, P. Butti, W. Buttinger, A. Buzatu, A. R. Buzykaev, S. Cabrera Urbán, D. Caforio, V. M. Cairo, O. Cakir, N. Calace, P. Calafiura, A. Calandri, G. Calderini, P. Calfayan, G. Callea, L. P. Caloba, S. Calvente Lopez, D. Calvet, S. Calvet, T. P. Calvet, R. Camacho Toro, S. Camarda, P. Camarri, D. Cameron, R. Caminal Armadans, C. Camincher, S. Campana, M. Campanelli, A. Camplani, A. Campoverde, V. Canale, A. Canepa, M. Cano Bret, J. Cantero, T. Cao, M. D. M. Capeans Garrido, I. Caprini, M. Caprini, M. Capua, R. M. Carbone, R. Cardarelli, F. Cardillo, I. Carli, T. Carli, G. Carlino, B. T. Carlson, L. Carminati, R. M. D. Carney, S. Caron, E. Carquin, G. D. Carrillo-Montoya, J. R. Carter, J. Carvalho, D. Casadei, M. P. Casado, M. Casolino, D. W. Casper, R. Castelijn, A. Castelli, V. Castillo Gimenez, N. F. Castro, A. Catinaccio, J. R. Catmore, A. Cattai, J. Caudron, V. Cavaliere, E. Cavallaro, D. Cavalli, M. Cavalli-Sforza, V. Cavasinni, F. Ceradini, L. Cerda Alberich, A. S. Cerqueira, A. Cerri, L. Cerrito, F. Cerutti, A. Cervelli, S. A. Cetin, A. Chafaq, D. Chakraborty, S. K. Chan, Y. L. Chan, P. Chang, J. D. Chapman, D. G. Charlton, A. Chatterjee, C. C. Chau, C. A. Chavez Barajas, S. Che, S. Cheatham, A. Chegwidden, S. Chekanov, S. V. Chekulaev, G. A. Chelkov, M. A. Chelstowska, C. Chen, H. Chen, S. Chen, S. Chen, X. Chen, Y. Chen, H. C. Cheng, H. J. Cheng, Y. Cheng, A. Cheplakov, E. Cheremushkina, R. Cherkaoui El Moursli, V. Chernyatin, E. Cheu, L. Chevalier, V. Chiarella, G. Chiarelli, G. Chiodini, A. S. Chisholm, A. Chitan, Y. H. Chiu, M. V. Chizhov, K. Choi, A. R. Chomont, S. Chouridou, B. K. B. Chow, V. Christodoulou, D. Chromek-Burckhart, J. Chudoba, A. J. Chuinard, J. J. Chwastowski, L. Chytka, A. K. Ciftci, D. Cinca, V. Cindro, I. A. Cioara, C. Ciocca, A. Ciocio, F. Cirotto, Z. H. Citron, M. Citterio, M. Ciubancan, A. Clark, B. L. Clark, M. R. Clark, P. J. Clark, R. N. Clarke, C. Clement, Y. Coadou, M. Cobal, A. Coccaro, J. Cochran, L. Colasurdo, B. Cole, A. P. Colijn, J. Collot, T. Colombo, P. Conde Muiño, E. Coniavitis, S. H. Connell, I. A. Connelly, V. Consorti, S. Constantinescu, G. Conti, F. Conventi, M. Cooke, B. D. Cooper, A. M. Cooper-Sarkar, F. Cormier, K. J. R. Cormier, T. Cornelissen, M. Corradi, F. Corriveau, A. Cortes-Gonzalez, G. Cortiana, G. Costa, M. J. Costa, D. Costanzo, G. Cottin, G. Cowan, B. E. Cox, K. Cranmer, S. J. Crawley, G. Cree, S. Crépé-Renaudin, F. Crescioli, W. A. Cribbs, M. Crispin Ortuzar, M. Cristinziani, V. Croft, G. Crosetti, A. Cueto, T. Cuhadar Donszelmann, J. Cummings, M. Curatolo, J. Cúth, H. Czirr, P. Czodrowski, G. D’amen, S. D’Auria, M. D’Onofrio, M. J. Da Cunha Sargedas De Sousa, C. Da Via, W. Dabrowski, T. Dado, T. Dai, O. Dale, F. Dallaire, C. Dallapiccola, M. Dam, J. R. Dandoy, N. P. Dang, A. C. Daniells, N. S. Dann, M. Danninger, M. Dano Hoffmann, V. Dao, G. Darbo, S. Darmora, J. Dassoulas, A. Dattagupta, T. Daubney, W. Davey, C. David, T. Davidek, M. Davies, P. Davison, E. Dawe, I. Dawson, K. De, R. de Asmundis, A. De Benedetti, S. De Castro, S. De Cecco, N. De Groot, P. de Jong, H. De la Torre, F. De Lorenzi, A. De Maria, D. De Pedis, A. De Salvo, U. De Sanctis, A. De Santo, J. B. De Vivie De Regie, W. J. Dearnaley, R. Debbe, C. Debenedetti, D. V. Dedovich, N. Dehghanian, I. Deigaard, M. Del Gaudio, J. Del Peso, T. Del Prete, D. Delgove, F. Deliot, C. M. Delitzsch, A. Dell’Acqua, L. Dell’Asta, M. Dell’Orso, M. Della Pietra, D. della Volpe, M. Delmastro, P. A. Delsart, D. A. DeMarco, S. Demers, M. Demichev, A. Demilly, S. P. Denisov, D. Denysiuk, D. Derendarz, J. E. Derkaoui, F. Derue, P. Dervan, K. Desch, C. Deterre, K. Dette, P. O. Deviveiros, A. Dewhurst, S. Dhaliwal, A. Di Ciaccio, L. Di Ciaccio, W. K. Di Clemente, C. Di Donato, A. Di Girolamo, B. Di Girolamo, B. Di Micco, R. Di Nardo, K. F. Di Petrillo, A. Di Simone, R. Di Sipio, D. Di Valentino, C. Diaconu, M. Diamond, F. A. Dias, M. A. Diaz, E. B. Diehl, J. Dietrich, S. Díez Cornell, A. Dimitrievska, J. Dingfelder, P. Dita, S. Dita, F. Dittus, F. Djama, T. Djobava, J. I. Djuvsland, M. A. B. do Vale, D. Dobos, M. Dobre, C. Doglioni, J. Dolejsi, Z. Dolezal, M. Donadelli, S. Donati, P. Dondero, J. Donini, J. Dopke, A. Doria, M. T. Dova, A. T. Doyle, E. Drechsler, M. Dris, Y. Du, J. Duarte-Campderros, E. Duchovni, G. Duckeck, O. A. Ducu, D. Duda, A. Dudarev, A. Chr. Dudder, E. M. Duffield, L. Duflot, M. Dührssen, M. Dumancic, A. K. Duncan, M. Dunford, H. Duran Yildiz, M. Düren, A. Durglishvili, D. Duschinger, B. Dutta, M. Dyndal, C. Eckardt, K. M. Ecker, R. C. Edgar, N. C. Edwards, T. Eifert, G. Eigen, K. Einsweiler, T. Ekelof, M. El Kacimi, V. Ellajosyula, M. Ellert, S. Elles, F. Ellinghaus, A. A. Elliot, N. Ellis, J. Elmsheuser, M. Elsing, D. Emeliyanov, Y. Enari, O. C. Endner, J. S. Ennis, J. Erdmann, A. Ereditato, G. Ernis, J. Ernst, M. Ernst, S. Errede, E. Ertel, M. Escalier, H. Esch, C. Escobar, B. Esposito, A. I. Etienvre, E. Etzion, H. Evans, A. Ezhilov, M. Ezzi, F. Fabbri, L. Fabbri, G. Facini, R. M. Fakhrutdinov, S. Falciano, R. J. Falla, J. Faltova, Y. Fang, M. Fanti, A. Farbin, A. Farilla, C. Farina, E. M. Farina, T. Farooque, S. Farrell, S. M. Farrington, P. Farthouat, F. Fassi, P. Fassnacht, D. Fassouliotis, M. Faucci Giannelli, A. Favareto, W. J. Fawcett, L. Fayard, O. L. Fedin, W. Fedorko, S. Feigl, L. Feligioni, C. Feng, E. J. Feng, H. Feng, A. B. Fenyuk, L. Feremenga, P. Fernandez Martinez, S. Fernandez Perez, J. Ferrando, A. Ferrari, P. Ferrari, R. Ferrari, D. E. Ferreira de Lima, A. Ferrer, D. Ferrere, C. Ferretti, F. Fiedler, A. Filipčič, M. Filipuzzi, F. Filthaut, M. Fincke-Keeler, K. D. Finelli, M. C. N. Fiolhais, L. Fiorini, A. Fischer, C. Fischer, J. Fischer, W. C. Fisher, N. Flaschel, I. Fleck, P. Fleischmann, G. T. Fletcher, R. R. M. Fletcher, T. Flick, B. M. Flierl, L. R. Flores Castillo, M. J. Flowerdew, G. T. Forcolin, A. Formica, A. Forti, A. G. Foster, D. Fournier, H. Fox, S. Fracchia, P. Francavilla, M. Franchini, D. Francis, L. Franconi, M. Franklin, M. Frate, M. Fraternali, D. Freeborn, S. M. Fressard-Batraneanu, D. Froidevaux, J. A. Frost, C. Fukunaga, E. Fullana Torregrosa, T. Fusayasu, J. Fuster, C. Gabaldon, O. Gabizon, A. Gabrielli, A. Gabrielli, G. P. Gach, S. Gadatsch, G. Gagliardi, L. G. Gagnon, P. Gagnon, C. Galea, B. Galhardo, E. J. Gallas, B. J. Gallop, P. Gallus, G. Galster, K. K. Gan, S. Ganguly, J. Gao, Y. Gao, Y. S. Gao, F. M. Garay Walls, C. García, J. E. García Navarro, M. Garcia-Sciveres, R. W. Gardner, N. Garelli, V. Garonne, A. Gascon Bravo, K. Gasnikova, C. Gatti, A. Gaudiello, G. Gaudio, L. Gauthier, I. L. Gavrilenko, C. Gay, G. Gaycken, E. N. Gazis, Z. Gecse, C. N. P. Gee, Ch. Geich-Gimbel, M. Geisen, M. P. Geisler, K. Gellerstedt, C. Gemme, M. H. Genest, C. Geng, S. Gentile, C. Gentsos, S. George, D. Gerbaudo, A. Gershon, S. Ghasemi, M. Ghneimat, B. Giacobbe, S. Giagu, P. Giannetti, S. M. Gibson, M. Gignac, M. Gilchriese, T. P. S. Gillam, D. Gillberg, G. Gilles, D. M. Gingrich, N. Giokaris, M. P. Giordani, F. M. Giorgi, P. F. Giraud, P. Giromini, D. Giugni, F. Giuli, C. Giuliani, M. Giulini, B. K. Gjelsten, S. Gkaitatzis, I. Gkialas, E. L. Gkougkousis, L. K. Gladilin, C. Glasman, J. Glatzer, P. C. F. Glaysher, A. Glazov, M. Goblirsch-Kolb, J. Godlewski, S. Goldfarb, T. Golling, D. Golubkov, A. Gomes, R. Gonçalo, R. Goncalves Gama, J. Goncalves Pinto Firmino Da Costa, G. Gonella, L. Gonella, A. Gongadze, S. González de la Hoz, S. Gonzalez-Sevilla, L. Goossens, P. A. Gorbounov, H. A. Gordon, I. Gorelov, B. Gorini, E. Gorini, A. Gorišek, A. T. Goshaw, C. Gössling, M. I. Gostkin, C. R. Goudet, D. Goujdami, A. G. Goussiou, N. Govender, E. Gozani, L. Graber, I. Grabowska-Bold, P. O. J. Gradin, P. Grafström, J. Gramling, E. Gramstad, S. Grancagnolo, V. Gratchev, P. M. Gravila, H. M. Gray, E. Graziani, Z. D. Greenwood, C. Grefe, K. Gregersen, I. M. Gregor, P. Grenier, K. Grevtsov, J. Griffiths, A. A. Grillo, K. Grimm, S. Grinstein, Ph. Gris, J. -F. Grivaz, S. Groh, E. Gross, J. Grosse-Knetter, G. C. Grossi, Z. J. Grout, L. Guan, W. Guan, J. Guenther, F. Guescini, D. Guest, O. Gueta, B. Gui, E. Guido, T. Guillemin, S. Guindon, U. Gul, C. Gumpert, J. Guo, W. Guo, Y. Guo, R. Gupta, S. Gupta, G. Gustavino, P. Gutierrez, N. G. Gutierrez Ortiz, C. Gutschow, C. Guyot, C. Gwenlan, C. B. Gwilliam, A. Haas, C. Haber, H. K. Hadavand, N. Haddad, A. Hadef, S. Hageböck, M. Hagihara, H. Hakobyan, M. Haleem, J. Haley, G. Halladjian, G. D. Hallewell, K. Hamacher, P. Hamal, K. Hamano, A. Hamilton, G. N. Hamity, P. G. Hamnett, L. Han, S. Han, K. Hanagaki, K. Hanawa, M. Hance, B. Haney, P. Hanke, R. Hanna, J. B. Hansen, J. D. Hansen, M. C. Hansen, P. H. Hansen, K. Hara, A. S. Hard, T. Harenberg, F. Hariri, S. Harkusha, R. D. Harrington, P. F. Harrison, F. Hartjes, N. M. Hartmann, M. Hasegawa, Y. Hasegawa, A. Hasib, S. Hassani, S. Haug, R. Hauser, L. Hauswald, M. Havranek, C. M. Hawkes, R. J. Hawkings, D. Hayakawa, D. Hayden, C. P. Hays, J. M. Hays, H. S. Hayward, S. J. Haywood, S. J. Head, T. Heck, V. Hedberg, L. Heelan, S. Heim, T. Heim, B. Heinemann, J. J. Heinrich, L. Heinrich, C. Heinz, J. Hejbal, L. Helary, S. Hellman, C. Helsens, J. Henderson, R. C. W. Henderson, Y. Heng, S. Henkelmann, A. M. Henriques Correia, S. Henrot-Versille, G. H. Herbert, H. Herde, V. Herget, Y. Hernández Jiménez, G. Herten, R. Hertenberger, L. Hervas, T. C. Herwig, G. G. Hesketh, N. P. Hessey, J. W. Hetherly, E. Higón-Rodriguez, E. Hill, J. C. Hill, K. H. Hiller, S. J. Hillier, I. Hinchliffe, E. Hines, M. Hirose, D. Hirschbuehl, O. Hladik, X. Hoad, J. Hobbs, N. Hod, M. C. Hodgkinson, P. Hodgson, A. Hoecker, M. R. Hoeferkamp, F. Hoenig, D. Hohn, T. R. Holmes, M. Homann, S. Honda, T. Honda, T. M. Hong, B. H. Hooberman, W. H. Hopkins, Y. Horii, A. J. Horton, J.-Y. Hostachy, S. Hou, A. Hoummada, J. Howarth, J. Hoya, M. Hrabovsky, I. Hristova, J. Hrivnac, T. Hryn’ova, A. Hrynevich, P. J. Hsu, S. -C. Hsu, Q. Hu, S. Hu, Y. Huang, Z. Hubacek, F. Hubaut, F. Huegging, T. B. Huffman, E. W. Hughes, G. Hughes, M. Huhtinen, P. Huo, N. Huseynov, J. Huston, J. Huth, G. Iacobucci, G. Iakovidis, I. Ibragimov, L. Iconomidou-Fayard, Z. Idrissi, P. Iengo, O. Igonkina, T. Iizawa, Y. Ikegami, M. Ikeno, Y. Ilchenko, D. Iliadis, N. Ilic, G. Introzzi, P. Ioannou, M. Iodice, K. Iordanidou, V. Ippolito, N. Ishijima, M. Ishino, M. Ishitsuka, C. Issever, S. Istin, F. Ito, J. M. Iturbe Ponce, R. Iuppa, H. Iwasaki, J. M. Izen, V. Izzo, S. Jabbar, P. Jackson, V. Jain, K. B. Jakobi, K. Jakobs, S. Jakobsen, T. Jakoubek, D. O. Jamin, D. K. Jana, R. Jansky, J. Janssen, M. Janus, P. A. Janus, G. Jarlskog, N. Javadov, T. Javůrek, M. Javurkova, F. Jeanneau, L. Jeanty, J. Jejelava, G. -Y. Jeng, P. Jenni, C. Jeske, S. Jézéquel, H. Ji, J. Jia, H. Jiang, Y. Jiang, Z. Jiang, S. Jiggins, J. Jimenez Pena, S. Jin, A. Jinaru, O. Jinnouchi, H. Jivan, P. Johansson, K. A. Johns, C. A. Johnson, W. J. Johnson, K. Jon-And, G. Jones, R. W. L. Jones, S. Jones, T. J. Jones, J. Jongmanns, P. M. Jorge, J. Jovicevic, X. Ju, A. Juste Rozas, M. K. Köhler, A. Kaczmarska, M. Kado, H. Kagan, M. Kagan, S. J. Kahn, T. Kaji, E. Kajomovitz, C. W. Kalderon, A. Kaluza, S. Kama, A. Kamenshchikov, N. Kanaya, S. Kaneti, L. Kanjir, V. A. Kantserov, J. Kanzaki, B. Kaplan, L. S. Kaplan, A. Kapliy, D. Kar, K. Karakostas, A. Karamaoun, N. Karastathis, M. J. Kareem, E. Karentzos, S. N. Karpov, Z. M. Karpova, K. Karthik, V. Kartvelishvili, A. N. Karyukhin, K. Kasahara, L. Kashif, R. D. Kass, A. Kastanas, Y. Kataoka, C. Kato, A. Katre, J. Katzy, K. Kawade, K. Kawagoe, T. Kawamoto, G. Kawamura, V. F. Kazanin, R. Keeler, R. Kehoe, J. S. Keller, J. J. Kempster, H. Keoshkerian, O. Kepka, B. P. Kerševan, S. Kersten, R. A. Keyes, M. Khader, F. Khalil-zada, A. Khanov, A. G. Kharlamov, T. Kharlamova, T. J. Khoo, V. Khovanskiy, E. Khramov, J. Khubua, S. Kido, C. R. Kilby, H. Y. Kim, S. H. Kim, Y. K. Kim, N. Kimura, O. M. Kind, B. T. King, M. King, D. Kirchmeier, J. Kirk, A. E. Kiryunin, T. Kishimoto, D. Kisielewska, K. Kiuchi, O. Kivernyk, E. Kladiva, T. Klapdor-Kleingrothaus, M. H. Klein, M. Klein, U. Klein, K. Kleinknecht, P. Klimek, A. Klimentov, R. Klingenberg, T. Klioutchnikova, E. -E. Kluge, P. Kluit, S. Kluth, J. Knapik, E. Kneringer, E. B. F. G. Knoops, A. Knue, A. Kobayashi, D. Kobayashi, T. Kobayashi, M. Kobel, M. Kocian, P. Kodys, T. Koffas, E. Koffeman, N. M. Köhler, T. Koi, H. Kolanoski, M. Kolb, I. Koletsou, A. A. Komar, Y. Komori, T. Kondo, N. Kondrashova, K. Köneke, A. C. König, T. Kono, R. Konoplich, N. Konstantinidis, R. Kopeliansky, S. Koperny, A. K. Kopp, K. Korcyl, K. Kordas, A. Korn, A. A. Korol, I. Korolkov, E. V. Korolkova, O. Kortner, S. Kortner, T. Kosek, V. V. Kostyukhin, A. Kotwal, A. Koulouris, A. Kourkoumeli-Charalampidi, C. Kourkoumelis, V. Kouskoura, A. B. Kowalewska, R. Kowalewski, T. Z. Kowalski, C. Kozakai, W. Kozanecki, A. S. Kozhin, V. A. Kramarenko, G. Kramberger, D. Krasnopevtsev, M. W. Krasny, A. Krasznahorkay, A. Kravchenko, M. Kretz, J. Kretzschmar, K. Kreutzfeldt, P. Krieger, K. Krizka, K. Kroeninger, H. Kroha, J. Kroll, J. Kroseberg, J. Krstic, U. Kruchonak, H. Krüger, N. Krumnack, M. C. Kruse, M. Kruskal, T. Kubota, H. Kucuk, S. Kuday, J. T. Kuechler, S. Kuehn, A. Kugel, F. Kuger, T. Kuhl, V. Kukhtin, R. Kukla, Y. Kulchitsky, S. Kuleshov, M. Kuna, T. Kunigo, A. Kupco, O. Kuprash, H. Kurashige, L. L. Kurchaninov, Y. A. Kurochkin, M. G. Kurth, V. Kus, E. S. Kuwertz, M. Kuze, J. Kvita, T. Kwan, D. Kyriazopoulos, A. La Rosa, J. L. La Rosa Navarro, L. La Rotonda, C. Lacasta, F. Lacava, J. Lacey, H. Lacker, D. Lacour, E. Ladygin, R. Lafaye, B. Laforge, T. Lagouri, S. Lai, S. Lammers, W. Lampl, E. Lançon, U. Landgraf, M. P. J. Landon, M. C. Lanfermann, V. S. Lang, J. C. Lange, A. J. Lankford, F. Lanni, K. Lantzsch, A. Lanza, A. Lapertosa, S. Laplace, C. Lapoire, J. F. Laporte, T. Lari, F. Lasagni Manghi, M. Lassnig, P. Laurelli, W. Lavrijsen, A. T. Law, P. Laycock, T. Lazovich, M. Lazzaroni, B. Le, O. Le Dortz, E. Le Guirriec, E. P. Le Quilleuc, M. LeBlanc, T. LeCompte, F. Ledroit-Guillon, C. A. Lee, S. C. Lee, L. Lee, B. Lefebvre, G. Lefebvre, M. Lefebvre, F. Legger, C. Leggett, A. Lehan, G. Lehmann Miotto, X. Lei, W. A. Leight, A. G. Leister, M. A. L. Leite, R. Leitner, D. Lellouch, B. Lemmer, K. J. C. Leney, T. Lenz, B. Lenzi, R. Leone, S. Leone, C. Leonidopoulos, S. Leontsinis, G. Lerner, C. Leroy, A. A. J. Lesage, C. G. Lester, M. Levchenko, J. Levêque, D. Levin, L. J. Levinson, M. Levy, D. Lewis, M. Leyton, B. Li, C. Li, H. Li, L. Li, L. Li, Q. Li, S. Li, X. Li, Y. Li, Z. Liang, B. Liberti, A. Liblong, P. Lichard, K. Lie, J. Liebal, W. Liebig, A. Limosani, S. C. Lin, T. H. Lin, B. E. Lindquist, A. E. Lionti, E. Lipeles, A. Lipniacka, M. Lisovyi, T. M. Liss, A. Lister, A. M. Litke, B. Liu, H. Liu, H. Liu, J. Liu, J. B. Liu, K. Liu, L. Liu, M. Liu, Y. L. Liu, Y. Liu, M. Livan, A. Lleres, J. Llorente Merino, S. L. Lloyd, F. Lo Sterzo, E. M. Lobodzinska, P. Loch, F. K. Loebinger, K. M. Loew, A. Loginov, T. Lohse, K. Lohwasser, M. Lokajicek, B. A. Long, J. D. Long, R. E. Long, L. Longo, K. A. Looper, J. A. Lopez, D. Lopez Mateos, B. Lopez Paredes, I. Lopez Paz, A. Lopez Solis, J. Lorenz, N. Lorenzo Martinez, M. Losada, P. J. Lösel, X. Lou, A. Lounis, J. Love, P. A. Love, H. Lu, N. Lu, H. J. Lubatti, C. Luci, A. Lucotte, C. Luedtke, F. Luehring, W. Lukas, L. Luminari, O. Lundberg, B. Lund-Jensen, P. M. Luzi, D. Lynn, R. Lysak, E. Lytken, V. Lyubushkin, H. Ma, L. L. Ma, Y. Ma, G. Maccarrone, A. Macchiolo, C. M. Macdonald, B. Maček, J. Machado Miguens, D. Madaffari, R. Madar, H. J. Maddocks, W. F. Mader, A. Madsen, J. Maeda, S. Maeland, T. Maeno, A. Maevskiy, E. Magradze, J. Mahlstedt, C. Maiani, C. Maidantchik, A. A. Maier, T. Maier, A. Maio, S. Majewski, Y. Makida, N. Makovec, B. Malaescu, Pa. Malecki, V. P. Maleev, F. Malek, U. Mallik, D. Malon, C. Malone, S. Maltezos, S. Malyukov, J. Mamuzic, G. Mancini, L. Mandelli, I. Mandić, J. Maneira, L. Manhaes de Andrade Filho, J. Manjarres Ramos, A. Mann, A. Manousos, B. Mansoulie, J. D. Mansour, R. Mantifel, M. Mantoani, S. Manzoni, L. Mapelli, G. Marceca, L. March, G. Marchiori, M. Marcisovsky, M. Marjanovic, D. E. Marley, F. Marroquim, S. P. Marsden, Z. Marshall, S. Marti-Garcia, T. A. Martin, V. J. Martin, B. Martin dit Latour, M. Martinez, V. I. Martinez Outschoorn, S. Martin-Haugh, V. S. Martoiu, A. C. Martyniuk, A. Marzin, L. Masetti, T. Mashimo, R. Mashinistov, J. Masik, A. L. Maslennikov, L. Massa, P. Mastrandrea, A. Mastroberardino, T. Masubuchi, P. Mättig, J. Mattmann, J. Maurer, S. J. Maxfield, D. A. Maximov, R. Mazini, I. Maznas, S. M. Mazza, N. C. Mc Fadden, G. Mc Goldrick, S. P. Mc Kee, A. McCarn, R. L. McCarthy, T. G. McCarthy, L. I. McClymont, E. F. McDonald, J. A. Mcfayden, G. Mchedlidze, S. J. McMahon, P. C. McNamara, R. A. McPherson, M. Medinnis, S. Meehan, S. Mehlhase, A. Mehta, K. Meier, C. Meineck, B. Meirose, D. Melini, B. R. Mellado Garcia, M. Melo, F. Meloni, S. B. Menary, L. Meng, X. T. Meng, A. Mengarelli, S. Menke, E. Meoni, S. Mergelmeyer, P. Mermod, L. Merola, C. Meroni, F. S. Merritt, A. Messina, J. Metcalfe, A. S. Mete, C. Meyer, J.-P. Meyer, J. Meyer, H. Meyer Zu Theenhausen, F. Miano, R. P. Middleton, S. Miglioranzi, L. Mijović, G. Mikenberg, M. Mikestikova, M. Mikuž, M. Milesi, A. Milic, D. W. Miller, C. Mills, A. Milov, D. A. Milstead, A. A. Minaenko, Y. Minami, I. A. Minashvili, A. I. Mincer, B. Mindur, M. Mineev, Y. Minegishi, Y. Ming, L. M. Mir, K. P. Mistry, T. Mitani, J. Mitrevski, V. A. Mitsou, A. Miucci, P. S. Miyagawa, A. Mizukami, J. U. Mjörnmark, M. Mlynarikova, T. Moa, K. Mochizuki, P. Mogg, S. Mohapatra, S. Molander, R. Moles-Valls, R. Monden, M. C. Mondragon, K. Mönig, J. Monk, E. Monnier, A. Montalbano, J. Montejo Berlingen, F. Monticelli, S. Monzani, R. W. Moore, N. Morange, D. Moreno, M. Moreno Llácer, P. Morettini, S. Morgenstern, D. Mori, T. Mori, M. Morii, M. Morinaga, V. Morisbak, S. Moritz, A. K. Morley, G. Mornacchi, J. D. Morris, S. S. Mortensen, L. Morvaj, P. Moschovakos, M. Mosidze, H. J. Moss, J. Moss, K. Motohashi, R. Mount, E. Mountricha, E. J. W. Moyse, S. Muanza, R. D. Mudd, F. Mueller, J. Mueller, R. S. P. Mueller, T. Mueller, D. Muenstermann, P. Mullen, G. A. Mullier, F. J. Munoz Sanchez, J. A. Murillo Quijada, W. J. Murray, H. Musheghyan, M. Muškinja, A. G. Myagkov, M. Myska, B. P. Nachman, O. Nackenhorst, K. Nagai, R. Nagai, K. Nagano, Y. Nagasaka, K. Nagata, M. Nagel, E. Nagy, A. M. Nairz, Y. Nakahama, K. Nakamura, T. Nakamura, I. Nakano, R. F. Naranjo Garcia, R. Narayan, D. I. Narrias Villar, I. Naryshkin, T. Naumann, G. Navarro, R. Nayyar, H. A. Neal, P. Yu. Nechaeva, T. J. Neep, A. Negri, M. Negrini, S. Nektarijevic, C. Nellist, A. Nelson, S. Nemecek, P. Nemethy, A. A. Nepomuceno, M. Nessi, M. S. Neubauer, M. Neumann, R. M. Neves, P. Nevski, P. R. Newman, T. Nguyen Manh, R. B. Nickerson, R. Nicolaidou, J. Nielsen, V. Nikolaenko, I. Nikolic-Audit, K. Nikolopoulos, J. K. Nilsen, P. Nilsson, Y. Ninomiya, A. Nisati, R. Nisius, T. Nobe, Y. Noguchi, M. Nomachi, I. Nomidis, T. Nooney, S. Norberg, M. Nordberg, N. Norjoharuddeen, O. Novgorodova, S. Nowak, M. Nozaki, L. Nozka, K. Ntekas, E. Nurse, F. Nuti, D. C. O’Neil, A. A. O’Rourke, V. O’Shea, F. G. Oakham, H. Oberlack, T. Obermann, J. Ocariz, A. Ochi, I. Ochoa, J. P. Ochoa-Ricoux, S. Oda, S. Odaka, H. Ogren, A. Oh, S. H. Oh, C. C. Ohm, H. Ohman, H. Oide, H. Okawa, Y. Okumura, T. Okuyama, A. Olariu, L. F. Oleiro Seabra, S. A. Olivares Pino, D. Oliveira Damazio, A. Olszewski, J. Olszowska, A. Onofre, K. Onogi, P. U. E. Onyisi, M. J. Oreglia, Y. Oren, D. Orestano, N. Orlando, R. S. Orr, B. Osculati, R. Ospanov, G. Otero y Garzon, H. Otono, M. Ouchrif, F. Ould-Saada, A. Ouraou, K. P. Oussoren, Q. Ouyang, M. Owen, R. E. Owen, V. E. Ozcan, N. Ozturk, K. Pachal, A. Pacheco Pages, L. Pacheco Rodriguez, C. Padilla Aranda, S. Pagan Griso, M. Paganini, F. Paige, P. Pais, K. Pajchel, G. Palacino, S. Palazzo, S. Palestini, M. Palka, D. Pallin, E. St. Panagiotopoulou, I. Panagoulias, C. E. Pandini, J. G. Panduro Vazquez, P. Pani, S. Panitkin, D. Pantea, L. Paolozzi, Th. D. Papadopoulou, K. Papageorgiou, A. Paramonov, D. Paredes Hernandez, A. J. Parker, M. A. Parker, K. A. Parker, F. Parodi, J. A. Parsons, U. Parzefall, V. R. Pascuzzi, E. Pasqualucci, S. Passaggio, Fr. Pastore, S. Pataraia, J. R. Pater, T. Pauly, J. Pearce, B. Pearson, L. E. Pedersen, S. Pedraza Lopez, R. Pedro, S. V. Peleganchuk, O. Penc, C. Peng, H. Peng, J. Penwell, B. S. Peralva, M. M. Perego, D. V. Perepelitsa, E. Perez Codina, L. Perini, H. Pernegger, S. Perrella, R. Peschke, V. D. Peshekhonov, K. Peters, R. F. Y. Peters, B. A. Petersen, T. C. Petersen, E. Petit, A. Petridis, C. Petridou, P. Petroff, E. Petrolo, M. Petrov, F. Petrucci, N. E. Pettersson, A. Peyaud, R. Pezoa, P. W. Phillips, G. Piacquadio, E. Pianori, A. Picazio, E. Piccaro, M. Piccinini, M. A. Pickering, R. Piegaia, J. E. Pilcher, A. D. Pilkington, A. W. J. Pin, M. Pinamonti, J. L. Pinfold, S. Pires, H. Pirumov, M. Pitt, L. Plazak, M. -A. Pleier, V. Pleskot, E. Plotnikova, D. Pluth, R. Poettgen, L. Poggioli, D. Pohl, G. Polesello, A. Poley, A. Policicchio, R. Polifka, A. Polini, C. S. Pollard, V. Polychronakos, K. Pommès, L. Pontecorvo, B. G. Pope, G. A. Popeneciu, A. Poppleton, S. Pospisil, K. Potamianos, I. N. Potrap, C. J. Potter, C. T. Potter, G. Poulard, J. Poveda, V. Pozdnyakov, M. E. Pozo Astigarraga, P. Pralavorio, A. Pranko, S. Prell, D. Price, L. E. Price, M. Primavera, S. Prince, K. Prokofiev, F. Prokoshin, S. Protopopescu, J. Proudfoot, M. Przybycien, D. Puddu, M. Purohit, P. Puzo, J. Qian, G. Qin, Y. Qin, A. Quadt, W. B. Quayle, M. Queitsch-Maitland, D. Quilty, S. Raddum, V. Radeka, V. Radescu, S. K. Radhakrishnan, P. Radloff, P. Rados, F. Ragusa, G. Rahal, J. A. Raine, S. Rajagopalan, M. Rammensee, C. Rangel-Smith, M. G. Ratti, D. M. Rauch, F. Rauscher, S. Rave, T. Ravenscroft, I. Ravinovich, M. Raymond, A. L. Read, N. P. Readioff, M. Reale, D. M. Rebuzzi, A. Redelbach, G. Redlinger, R. Reece, R. G. Reed, K. Reeves, L. Rehnisch, J. Reichert, A. Reiss, C. Rembser, H. Ren, M. Rescigno, S. Resconi, E. D. Resseguie, O. L. Rezanova, P. Reznicek, R. Rezvani, R. Richter, S. Richter, E. Richter-Was, O. Ricken, M. Ridel, P. Rieck, C. J. Riegel, J. Rieger, O. Rifki, M. Rijssenbeek, A. Rimoldi, M. Rimoldi, L. Rinaldi, G. Ripellino, B. Ristić, E. Ritsch, I. Riu, F. Rizatdinova, E. Rizvi, C. Rizzi, R. T. Roberts, S. H. Robertson, A. Robichaud-Veronneau, D. Robinson, J. E. M. Robinson, A. Robson, C. Roda, Y. Rodina, A. Rodriguez Perez, D. Rodriguez Rodriguez, S. Roe, C. S. Rogan, O. Røhne, J. Roloff, A. Romaniouk, M. Romano, S. M. Romano Saez, E. Romero Adam, N. Rompotis, M. Ronzani, L. Roos, E. Ros, S. Rosati, K. Rosbach, P. Rose, N. -A. Rosien, V. Rossetti, E. Rossi, L. P. Rossi, J. H. N. Rosten, R. Rosten, M. Rotaru, I. Roth, J. Rothberg, D. Rousseau, A. Rozanov, Y. Rozen, X. Ruan, F. Rubbo, M. S. Rudolph, F. Rühr, A. Ruiz-Martinez, Z. Rurikova, N. A. Rusakovich, A. Ruschke, H. L. Russell, J. P. Rutherfoord, N. Ruthmann, Y. F. Ryabov, M. Rybar, G. Rybkin, S. Ryu, A. Ryzhov, G. F. Rzehorz, A. F. Saavedra, G. Sabato, S. Sacerdoti, H. F-W. Sadrozinski, R. Sadykov, F. Safai Tehrani, P. Saha, M. Sahinsoy, M. Saimpert, T. Saito, H. Sakamoto, Y. Sakurai, G. Salamanna, J. E. Salazar Loyola, D. Salek, P. H. Sales De Bruin, D. Salihagic, A. Salnikov, J. Salt, D. Salvatore, F. Salvatore, A. Salvucci, A. Salzburger, D. Sammel, D. Sampsonidis, J. Sánchez, V. Sanchez Martinez, A. Sanchez Pineda, H. Sandaker, R. L. Sandbach, M. Sandhoff, C. Sandoval, D. P. C. Sankey, M. Sannino, A. Sansoni, C. Santoni, R. Santonico, H. Santos, I. Santoyo Castillo, K. Sapp, A. Sapronov, J. G. Saraiva, B. Sarrazin, O. Sasaki, K. Sato, E. Sauvan, G. Savage, P. Savard, N. Savic, C. Sawyer, L. Sawyer, J. Saxon, C. Sbarra, A. Sbrizzi, T. Scanlon, D. A. Scannicchio, M. Scarcella, V. Scarfone, J. Schaarschmidt, P. Schacht, B. M. Schachtner, D. Schaefer, L. Schaefer, R. Schaefer, J. Schaeffer, S. Schaepe, S. Schaetzel, U. Schäfer, A. C. Schaffer, D. Schaile, R. D. Schamberger, V. Scharf, V. A. Schegelsky, D. Scheirich, M. Schernau, C. Schiavi, S. Schier, C. Schillo, M. Schioppa, S. Schlenker, K. R. Schmidt-Sommerfeld, K. Schmieden, C. Schmitt, S. Schmitt, S. Schmitz, B. Schneider, U. Schnoor, L. Schoeffel, A. Schoening, B. D. Schoenrock, E. Schopf, M. Schott, J. F. P. Schouwenberg, J. Schovancova, S. Schramm, M. Schreyer, N. Schuh, A. Schulte, M. J. Schultens, H. -C. Schultz-Coulon, H. Schulz, M. Schumacher, B. A. Schumm, Ph. Schune, A. Schwartzman, T. A. Schwarz, H. Schweiger, Ph. Schwemling, R. Schwienhorst, J. Schwindling, T. Schwindt, G. Sciolla, F. Scuri, F. Scutti, J. Searcy, P. Seema, S. C. Seidel, A. Seiden, F. Seifert, J. M. Seixas, G. Sekhniaidze, K. Sekhon, S. J. Sekula, N. Semprini-Cesari, C. Serfon, L. Serin, L. Serkin, M. Sessa, R. Seuster, H. Severini, T. Sfiligoj, F. Sforza, A. Sfyrla, E. Shabalina, N. W. Shaikh, L. Y. Shan, R. Shang, J. T. Shank, M. Shapiro, P. B. Shatalov, K. Shaw, S. M. Shaw, A. Shcherbakova, C. Y. Shehu, Y. Shen, P. Sherwood, L. Shi, S. Shimizu, C. O. Shimmin, M. Shimojima, S. Shirabe, M. Shiyakova, J. Shlomi, A. Shmeleva, D. Shoaleh Saadi, M. J. Shochet, S. Shojaii, D. R. Shope, S. Shrestha, E. Shulga, M. A. Shupe, P. Sicho, A. M. Sickles, P. E. Sidebo, E. Sideras Haddad, O. Sidiropoulou, D. Sidorov, A. Sidoti, F. Siegert, Dj. Sijacki, J. Silva, S. B. Silverstein, V. Simak, Lj. Simic, S. Simion, E. Simioni, B. Simmons, M. Simon, P. Sinervo, N. B. Sinev, M. Sioli, G. Siragusa, I. Siral, S. Yu. Sivoklokov, J. Sjölin, M. B. Skinner, P. Skubic, M. Slater, T. Slavicek, M. Slawinska, K. Sliwa, R. Slovak, V. Smakhtin, B. H. Smart, L. Smestad, J. Smiesko, S. Yu. Smirnov, Y. Smirnov, L. N. Smirnova, O. Smirnova, J. W. Smith, M. N. K. Smith, R. W. Smith, M. Smizanska, K. Smolek, A. A. Snesarev, I. M. Snyder, S. Snyder, R. Sobie, F. Socher, A. Soffer, D. A. Soh, G. Sokhrannyi, C. A. Solans Sanchez, M. Solar, E. Yu. Soldatov, U. Soldevila, A. A. Solodkov, A. Soloshenko, O. V. Solovyanov, V. Solovyev, P. Sommer, H. Son, H. Y. Song, A. Sood, A. Sopczak, V. Sopko, V. Sorin, D. Sosa, C. L. Sotiropoulou, R. Soualah, A. M. Soukharev, D. South, B. C. Sowden, S. Spagnolo, M. Spalla, M. Spangenberg, F. Spanò, D. Sperlich, F. Spettel, T. M. Spieker, R. Spighi, G. Spigo, L. A. Spiller, M. Spousta, R. D. St. Denis, A. Stabile, R. Stamen, S. Stamm, E. Stanecka, R. W. Stanek, C. Stanescu, M. Stanescu-Bellu, M. M. Stanitzki, S. Stapnes, E. A. Starchenko, G. H. Stark, J. Stark, P. Staroba, P. Starovoitov, S. Stärz, R. Staszewski, P. Steinberg, B. Stelzer, H. J. Stelzer, O. Stelzer-Chilton, H. Stenzel, G. A. Stewart, J. A. Stillings, M. C. Stockton, M. Stoebe, G. Stoicea, P. Stolte, S. Stonjek, A. R. Stradling, A. Straessner, M. E. Stramaglia, J. Strandberg, S. Strandberg, A. Strandlie, M. Strauss, P. Strizenec, R. Ströhmer, D. M. Strom, R. Stroynowski, A. Strubig, S. A. Stucci, B. Stugu, N. A. Styles, D. Su, J. Su, S. Suchek, Y. Sugaya, M. Suk, V. V. Sulin, S. Sultansoy, T. Sumida, S. Sun, X. Sun, K. Suruliz, C. J. E. Suster, M. R. Sutton, S. Suzuki, M. Svatos, M. Swiatlowski, S. P. Swift, I. Sykora, T. Sykora, D. Ta, K. Tackmann, J. Taenzer, A. Taffard, R. Tafirout, N. Taiblum, H. Takai, R. Takashima, T. Takeshita, Y. Takubo, M. Talby, A. A. Talyshev, J. Tanaka, M. Tanaka, R. Tanaka, S. Tanaka, R. Tanioka, B. B. Tannenwald, S. Tapia Araya, S. Tapprogge, S. Tarem, G. F. Tartarelli, P. Tas, M. Tasevsky, T. Tashiro, E. Tassi, A. Tavares Delgado, Y. Tayalati, A. C. Taylor, G. N. Taylor, P. T. E. Taylor, W. Taylor, F. A. Teischinger, P. Teixeira-Dias, K. K. Temming, D. Temple, H. Ten Kate, P. K. Teng, J. J. Teoh, F. Tepel, S. Terada, K. Terashi, J. Terron, S. Terzo, M. Testa, R. J. Teuscher, T. Theveneaux-Pelzer, J. P. Thomas, J. Thomas-Wilsker, P. D. Thompson, A. S. Thompson, L. A. Thomsen, E. Thomson, M. J. Tibbetts, R. E. Ticse Torres, V. O. Tikhomirov, Yu. A. Tikhonov, S. Timoshenko, P. Tipton, S. Tisserant, K. Todome, S. Todorova-Nova, J. Tojo, S. Tokár, K. Tokushuku, E. Tolley, L. Tomlinson, M. Tomoto, L. Tompkins, K. Toms, B. Tong, P. Tornambe, E. Torrence, H. Torres, E. Torró Pastor, J. Toth, F. Touchard, D. R. Tovey, T. Trefzger, A. Tricoli, I. M. Trigger, S. Trincaz-Duvoid, M. F. Tripiana, W. Trischuk, B. Trocmé, A. Trofymov, C. Troncon, M. Trottier-McDonald, M. Trovatelli, L. Truong, M. Trzebinski, A. Trzupek, J. C-L. Tseng, P. V. Tsiareshka, G. Tsipolitis, N. Tsirintanis, S. Tsiskaridze, V. Tsiskaridze, E. G. Tskhadadze, K. M. Tsui, I. I. Tsukerman, V. Tsulaia, S. Tsuno, D. Tsybychev, Y. Tu, A. Tudorache, V. Tudorache, T. T. Tulbure, A. N. Tuna, S. A. Tupputi, S. Turchikhin, D. Turgeman, I. Turk Cakir, R. Turra, P. M. Tuts, G. Ucchielli, I. Ueda, M. Ughetto, F. Ukegawa, G. Unal, A. Undrus, G. Unel, F. C. Ungaro, Y. Unno, C. Unverdorben, J. Urban, P. Urquijo, P. Urrejola, G. Usai, J. Usui, L. Vacavant, V. Vacek, B. Vachon, C. Valderanis, E. Valdes Santurio, N. Valencic, S. Valentinetti, A. Valero, L. Valery, S. Valkar, J. A. Valls Ferrer, W. Van Den Wollenberg, P. C. Van Der Deijl, H. van der Graaf, N. van Eldik, P. van Gemmeren, J. Van Nieuwkoop, I. van Vulpen, M. C. van Woerden, M. Vanadia, W. Vandelli, R. Vanguri, A. Vaniachine, P. Vankov, G. Vardanyan, R. Vari, E. W. Varnes, T. Varol, D. Varouchas, A. Vartapetian, K. E. Varvell, J. G. Vasquez, G. A. Vasquez, F. Vazeille, T. Vazquez Schroeder, J. Veatch, V. Veeraraghavan, L. M. Veloce, F. Veloso, S. Veneziano, A. Ventura, M. Venturi, N. Venturi, A. Venturini, V. Vercesi, M. Verducci, W. Verkerke, J. C. Vermeulen, M. C. Vetterli, O. Viazlo, I. Vichou, T. Vickey, O. E. Vickey Boeriu, G. H. A. Viehhauser, S. Viel, L. Vigani, M. Villa, M. Villaplana Perez, E. Vilucchi, M. G. Vincter, V. B. Vinogradov, A. Vishwakarma, C. Vittori, I. Vivarelli, S. Vlachos, M. Vlasak, M. Vogel, P. Vokac, G. Volpi, M. Volpi, H. von der Schmitt, E. von Toerne, V. Vorobel, K. Vorobev, M. Vos, R. Voss, J. H. Vossebeld, N. Vranjes, M. Vranjes Milosavljevic, V. Vrba, M. Vreeswijk, R. Vuillermet, I. Vukotic, P. Wagner, W. Wagner, H. Wahlberg, S. Wahrmund, J. Wakabayashi, J. Walder, R. Walker, W. Walkowiak, V. Wallangen, C. Wang, C. Wang, F. Wang, H. Wang, H. Wang, J. Wang, J. Wang, K. Wang, Q. Wang, R. Wang, S. M. Wang, T. Wang, W. Wang, C. Wanotayaroj, A. Warburton, C. P. Ward, D. R. Wardrope, A. Washbrook, P. M. Watkins, A. T. Watson, M. F. Watson, G. Watts, S. Watts, B. M. Waugh, S. Webb, M. S. Weber, S. W. Weber, S. A. Weber, J. S. Webster, A. R. Weidberg, B. Weinert, J. Weingarten, C. Weiser, H. Weits, P. S. Wells, T. Wenaus, T. Wengler, S. Wenig, N. Wermes, M. D. Werner, P. Werner, M. Wessels, J. Wetter, K. Whalen, N. L. Whallon, A. M. Wharton, A. White, M. J. White, R. White, D. Whiteson, F. J. Wickens, W. Wiedenmann, M. Wielers, C. Wiglesworth, L. A. M. Wiik-Fuchs, A. Wildauer, F. Wilk, H. G. Wilkens, H. H. Williams, S. Williams, C. Willis, S. Willocq, J. A. Wilson, I. Wingerter-Seez, F. Winklmeier, O. J. Winston, B. T. Winter, M. Wittgen, M. Wobisch, T. M. H. Wolf, R. Wolff, M. W. Wolter, H. Wolters, S. D. Worm, B. K. Wosiek, J. Wotschack, M. J. Woudstra, K. W. Wozniak, M. Wu, M. Wu, S. L. Wu, X. Wu, Y. Wu, T. R. Wyatt, B. M. Wynne, S. Xella, Z. Xi, D. Xu, L. Xu, B. Yabsley, S. Yacoob, D. Yamaguchi, Y. Yamaguchi, A. Yamamoto, S. Yamamoto, T. Yamanaka, K. Yamauchi, Y. Yamazaki, Z. Yan, H. Yang, H. Yang, Y. Yang, Z. Yang, W-M. Yao, Y. C. Yap, Y. Yasu, E. Yatsenko, K. H. Yau Wong, J. Ye, S. Ye, I. Yeletskikh, E. Yildirim, K. Yorita, R. Yoshida, K. Yoshihara, C. Young, C. J. S. Young, S. Youssef, D. R. Yu, J. Yu, J. Yu, L. Yuan, S. P. Y. Yuen, I. Yusuff, B. Zabinski, G. Zacharis, R. Zaidan, A. M. Zaitsev, N. Zakharchuk, J. Zalieckas, A. Zaman, S. Zambito, D. Zanzi, C. Zeitnitz, M. Zeman, A. Zemla, J. C. Zeng, Q. Zeng, O. Zenin, T. Ženiš, D. Zerwas, D. Zhang, F. Zhang, G. Zhang, H. Zhang, J. Zhang, L. Zhang, L. Zhang, M. Zhang, R. Zhang, R. Zhang, X. Zhang, Y. Zhang, Z. Zhang, X. Zhao, Y. Zhao, Z. Zhao, A. Zhemchugov, J. Zhong, B. Zhou, C. Zhou, L. Zhou, M. Zhou, M. Zhou, N. Zhou, C. G. Zhu, H. Zhu, J. Zhu, Y. Zhu, X. Zhuang, K. Zhukov, A. Zibell, D. Zieminska, N. I. Zimine, C. Zimmermann, S. Zimmermann, Z. Zinonos, M. Zinser, M. Ziolkowski, L. Živković, G. Zobernig, A. Zoccoli, M. zur Nedden, L. Zwalinski, ATLAS Collaboration

**Affiliations:** 1 Department of Physics, University of Adelaide, Adelaide, SA Australia; 2 Physics Department, SUNY Albany, Albany, NY USA; 3 Department of Physics, University of Alberta, Edmonton, AB Canada; 4 Department of Physics, Ankara University, Ankara, Turkey; 5 Istanbul Aydin University, Istanbul, Turkey; 6 Division of Physics, TOBB University of Economics and Technology, Ankara, Turkey; 7 LAPP, CNRS/IN2P3 and Université Savoie Mont Blanc, Annecy-le-Vieux, France; 8 High Energy Physics Division, Argonne National Laboratory, Argonne, IL USA; 9 Department of Physics, University of Arizona, Tucson, AZ USA; 10 Department of Physics, The University of Texas at Arlington, Arlington, TX USA; 11 Physics Department, National and Kapodistrian University of Athens, Athens, Greece; 12 Physics Department, National Technical University of Athens, Zografou, Greece; 13 Department of Physics, The University of Texas at Austin, Austin, TX USA; 14 Institute of Physics, Azerbaijan Academy of Sciences, Baku, Azerbaijan; 15 Institut de Física d’Altes Energies (IFAE), The Barcelona Institute of Science and Technology, Barcelona, Spain; 16 Institute of Physics, University of Belgrade, Belgrade, Serbia; 17 Department for Physics and Technology, University of Bergen, Bergen, Norway; 18 Physics Division, Lawrence Berkeley National Laboratory and University of California, Berkeley, CA USA; 19 Department of Physics, Humboldt University, Berlin, Germany; 20 Albert Einstein Center for Fundamental Physics and Laboratory for High Energy Physics, University of Bern, Bern, Switzerland; 21 School of Physics and Astronomy, University of Birmingham, Birmingham, UK; 22 Department of Physics, Bogazici University, Istanbul, Turkey; 23 Department of Physics Engineering, Gaziantep University, Gaziantep, Turkey; 24 Istanbul Bilgi University, Faculty of Engineering and Natural Sciences, Istanbul, Turkey; 25 Bahcesehir University, Faculty of Engineering and Natural Sciences, Istanbul, Turkey; 26 Centro de Investigaciones, Universidad Antonio Narino, Bogota, Colombia; 27 INFN Sezione di Bologna, Bologna, Italy; 28 Dipartimento di Fisica e Astronomia, Università di Bologna, Bologna, Italy; 29 Physikalisches Institut, University of Bonn, Bonn, Germany; 30 Department of Physics, Boston University, Boston, MA USA; 31 Department of Physics, Brandeis University, Waltham, MA USA; 32 Universidade Federal do Rio De Janeiro COPPE/EE/IF, Rio de Janeiro, Brazil; 33 Electrical Circuits Department, Federal University of Juiz de Fora (UFJF), Juiz de Fora, Brazil; 34 Federal University of Sao Joao del Rei (UFSJ), Sao Joao del Rei, Brazil; 35 Instituto de Fisica, Universidade de Sao Paulo, Sao Paulo, Brazil; 36 Physics Department, Brookhaven National Laboratory, Upton, NY USA; 37 Transilvania University of Brasov, Brasov, Romania; 38 Horia Hulubei National Institute of Physics and Nuclear Engineering, Bucharest, Romania; 39 Physics Department, National Institute for Research and Development of Isotopic and Molecular Technologies, Cluj Napoca, Romania; 41 West University in Timisoara, Timisoara, Romania; 42 Departamento de Física, Universidad de Buenos Aires, Buenos Aires, Argentina; 43 Cavendish Laboratory, University of Cambridge, Cambridge, UK; 44 Department of Physics, Carleton University, Ottawa, ON Canada; 45 CERN, Geneva, Switzerland; 46 Enrico Fermi Institute, University of Chicago, Chicago, IL USA; 47 Departamento de Física, Pontificia Universidad Católica de Chile, Santiago, Chile; 48 Departamento de Física, Universidad Técnica Federico Santa María, Valparaiso, Chile; 49 Institute of High Energy Physics, Chinese Academy of Sciences, Beijing, China; 50 Department of Physics, Nanjing University, Jiangsu, China; 51 Physics Department, Tsinghua University, Beijing, 100084 China; 52 Department of Modern Physics, University of Science and Technology of China, Anhui, China; 53 School of Physics, Shandong University, Shandong, China; 54 Department of Physics and Astronomy, Shanghai Key Laboratory for Particle Physics and Cosmology, Shanghai Jiao Tong University (also affiliated with PKU-CHEP), Shanghai, China; 55 Laboratoire de Physique Corpusculaire, Université Clermont Auvergne niversité Blaise Pascal, CNRS/IN2P3, Clermont-Ferrand, France; 56 Nevis Laboratory, Columbia University, Irvington, NY USA; 57 Niels Bohr Institute, University of Copenhagen, Copenhagen, Denmark; 58 INFN Gruppo Collegato di Cosenza, Laboratori Nazionali di Frascati, Frascati, Italy; 59 Dipartimento di Fisica, Università della Calabria, Rende, Italy; 60 Faculty of Physics and Applied Computer Science, AGH University of Science and Technology, Krakow, Poland; 61 Marian Smoluchowski Institute of Physics, Jagiellonian University, Kraków, Poland; 62 Institute of Nuclear Physics, Polish Academy of Sciences, Kraków, Poland; 63 Physics Department, Southern Methodist University, Dallas, TX USA; 64 Physics Department, University of Texas at Dallas, Richardson, TX USA; 65 DESY, Hamburg and Zeuthen, Germany; 66 Lehrstuhl für Experimentelle Physik IV, Technische Universität Dortmund, Dortmund, Germany; 67 Institut für Kern-und Teilchenphysik, Technische Universität Dresden, Dresden, Germany; 68 Department of Physics, Duke University, Durham, NC USA; 69 SUPA-School of Physics and Astronomy, University of Edinburgh, Edinburgh, UK; 70 INFN Laboratori Nazionali di Frascati, Frascati, Italy; 71 Fakultät für Mathematik und Physik, Albert-Ludwigs-Universität, Freiburg, Germany; 72 Departement de Physique Nucleaire et Corpusculaire, Université de Genève, Geneva, Switzerland; 73 INFN Sezione di Genova, Genoa, Italy; 74 Dipartimento di Fisica, Università di Genova, Genoa, Italy; 75 E. Andronikashvili Institute of Physics, Iv. Javakhishvili Tbilisi State University, Tbilisi, Georgia; 76 High Energy Physics Institute, Tbilisi State University, Tbilisi, Georgia; 77 II Physikalisches Institut, Justus-Liebig-Universität Giessen, Giessen, Germany; 78 SUPA-School of Physics and Astronomy, University of Glasgow, Glasgow, UK; 79 II Physikalisches Institut, Georg-August-Universität, Göttingen, Germany; 80 Laboratoire de Physique Subatomique et de Cosmologie, Université Grenoble-Alpes, CNRS/IN2P3, Grenoble, France; 81 Laboratory for Particle Physics and Cosmology, Harvard University, Cambridge, MA USA; 82 Kirchhoff-Institut für Physik, Ruprecht-Karls-Universität Heidelberg, Heidelberg, Germany; 83 Physikalisches Institut, Ruprecht-Karls-Universität Heidelberg, Heidelberg, Germany; 84 ZITI Institut für technische Informatik, Ruprecht-Karls-Universität Heidelberg, Mannheim, Germany; 85 Faculty of Applied Information Science, Hiroshima Institute of Technology, Hiroshima, Japan; 86 Department of Physics, The Chinese University of Hong Kong, Shatin, NT Hong Kong; 87 Department of Physics, The University of Hong Kong, Hong Kong, China; 88 Department of Physics and Institute for Advanced Study, The Hong Kong University of Science and Technology, Clear Water Bay, Kowloon, Hong Kong, China; 89 Department of Physics, National Tsing Hua University, Hsinchu, Taiwan Taiwan; 90 Department of Physics, Indiana University, Bloomington, IN USA; 91 Institut für Astro- und Teilchenphysik, Leopold-Franzens-Universität, Innsbruck, Austria; 92 University of Iowa, Iowa City, IA USA; 93 Department of Physics and Astronomy, Iowa State University, Ames, IA USA; 94 Joint Institute for Nuclear Research, JINR Dubna, Dubna, Russia; 95 KEK, High Energy Accelerator Research Organization, Tsukuba, Japan; 96 Graduate School of Science, Kobe University, Kobe, Japan; 97 Faculty of Science, Kyoto University, Kyoto, Japan; 98 Kyoto University of Education, Kyoto, Japan; 99 Department of Physics, Kyushu University, Fukuoka, Japan; 100 Instituto de Física La Plata, Universidad Nacional de La Plata and CONICET, La Plata, Argentina; 101 Physics Department, Lancaster University, Lancaster, UK; 102 INFN Sezione di Lecce, Lecce, Italy; 103 Dipartimento di Matematica e Fisica, Università del Salento, Lecce, Italy; 104 Oliver Lodge Laboratory, University of Liverpool, Liverpool, UK; 105 Department of Experimental Particle Physics, Jožef Stefan Institute and Department of Physics, University of Ljubljana, Ljubljana, Slovenia; 106 School of Physics and Astronomy, Queen Mary University of London, London, UK; 107 Department of Physics, Royal Holloway University of London, Surrey, UK; 108 Department of Physics and Astronomy, University College London, London, UK; 109 Louisiana Tech University, Ruston, LA USA; 110 Laboratoire de Physique Nucléaire et de Hautes Energies, UPMC and Université Paris-Diderot and CNRS/IN2P3, Paris, France; 111 Fysiska institutionen, Lunds universitet, Lund, Sweden; 112 Departamento de Fisica Teorica C-15, Universidad Autonoma de Madrid, Madrid, Spain; 113 Institut für Physik, Universität Mainz, Mainz, Germany; 114 School of Physics and Astronomy, University of Manchester, Manchester, UK; 115 CPPM, Aix-Marseille Université and CNRS/IN2P3, Marseille, France; 116 Department of Physics, University of Massachusetts, Amherst, MA USA; 117 Department of Physics, McGill University, Montreal, QC Canada; 118 School of Physics, University of Melbourne, Melbourne, VIC Australia; 119 Department of Physics, The University of Michigan, Ann Arbor, MI USA; 120 Department of Physics and Astronomy, Michigan State University, East Lansing, MI USA; 121 INFN Sezione di Milano, Milan, Italy; 122 Dipartimento di Fisica, Università di Milano, Milan, Italy; 123 B.I. Stepanov Institute of Physics, National Academy of Sciences of Belarus, Minsk, Republic of Belarus; 124 Research Institute for Nuclear Problems of Byelorussian State University, Minsk, Republic of Belarus; 125 Group of Particle Physics, University of Montreal, Montreal, QC Canada; 126 P.N. Lebedev Physical Institute of the Russian Academy of Sciences, Moscow, Russia; 127 Institute for Theoretical and Experimental Physics (ITEP), Moscow, Russia; 128 National Research Nuclear University MEPhI, Moscow, Russia; 129 D.V. Skobeltsyn Institute of Nuclear Physics, M.V. Lomonosov Moscow State University, Moscow, Russia; 130 Fakultät für Physik, Ludwig-Maximilians-Universität München, Munich, Germany; 131 Max-Planck-Institut für Physik (Werner-Heisenberg-Institut), Munich, Germany; 132 Nagasaki Institute of Applied Science, Nagasaki, Japan; 133 Graduate School of Science and Kobayashi-Maskawa Institute, Nagoya University, Nagoya, Japan; 134 INFN Sezione di Napoli, Naples, Italy; 135 Dipartimento di Fisica, Università di Napoli, Naples, Italy; 136 Department of Physics and Astronomy, University of New Mexico, Albuquerque, NM USA; 137 Institute for Mathematics, Astrophysics and Particle Physics, Radboud University Nijmegen/Nikhef, Nijmegen, The Netherlands; 138 Nikhef National Institute for Subatomic Physics and University of Amsterdam, Amsterdam, The Netherlands; 139 Department of Physics, Northern Illinois University, DeKalb, IL USA; 140 Budker Institute of Nuclear Physics, SB RAS, Novosibirsk, Russia; 141 Department of Physics, New York University, New York, NY USA; 142 Ohio State University, Columbus, OH USA; 143 Faculty of Science, Okayama University, Okayama, Japan; 144 Homer L. Dodge Department of Physics and Astronomy, University of Oklahoma, Norman, OK USA; 145 Department of Physics, Oklahoma State University, Stillwater, OK USA; 146 Palacký University, RCPTM, Olomouc, Czech Republic; 147 Center for High Energy Physics, University of Oregon, Eugene, OR USA; 148 LAL, Univ. Paris-Sud, CNRS/IN2P3, Université Paris-Saclay, Orsay, France; 149 Graduate School of Science, Osaka University, Osaka, Japan; 150 Department of Physics, University of Oslo, Oslo, Norway; 151 Department of Physics, Oxford University, Oxford, UK; 152 INFN Sezione di Pavia, Pavia, Italy; 153 Dipartimento di Fisica, Università di Pavia, Pavia, Italy; 154 Department of Physics, University of Pennsylvania, Philadelphia, PA USA; 155 National Research Centre “Kurchatov Institute” B.P.Konstantinov Petersburg Nuclear Physics Institute, St. Petersburg, Russia; 156 INFN Sezione di Pisa, Pisa, Italy; 157 Dipartimento di Fisica E. Fermi, Università di Pisa, Pisa, Italy; 158 Department of Physics and Astronomy, University of Pittsburgh, Pittsburgh, PA USA; 159 Laboratório de Instrumentação e Física Experimental de Partículas-LIP, Lisbon, Portugal; 160 Faculdade de Ciências, Universidade de Lisboa, Lisbon, Portugal; 161 Department of Physics, University of Coimbra, Coimbra, Portugal; 162 Centro de Física Nuclear da Universidade de Lisboa, Lisbon, Portugal; 163 Departamento de Fisica, Universidade do Minho, Braga, Portugal; 164 Departamento de Fisica Teorica y del Cosmos and CAFPE, Universidad de Granada, Granada, Spain; 166 Institute of Physics, Academy of Sciences of the Czech Republic, Prague, Czech Republic; 167 Czech Technical University in Prague, Prague, Czech Republic; 168 Faculty of Mathematics and Physics, Charles University in Prague, Prague, Czech Republic; 169 State Research Center Institute for High Energy Physics (Protvino), NRC KI, Russia; 170 Particle Physics Department, Rutherford Appleton Laboratory, Didcot, UK; 171 INFN Sezione di Roma, Rome, Italy; 172 Dipartimento di Fisica, Sapienza Università di Roma, Rome, Italy; 173 INFN Sezione di Roma Tor Vergata, Rome, Italy; 174 Dipartimento di Fisica, Università di Roma Tor Vergata, Rome, Italy; 175 INFN Sezione di Roma Tre, Rome, Italy; 176 Dipartimento di Matematica e Fisica, Università Roma Tre, Rome, Italy; 177 Faculté des Sciences Ain Chock, Réseau Universitaire de Physique des Hautes Energies-Université Hassan II, Casablanca, Morocco; 179 Faculté des Sciences Semlalia, Université Cadi Ayyad, LPHEA-Marrakech, Marrakech, Morocco; 180 Faculté des Sciences, Université Mohamed Premier and LPTPM, Oujda, Morocco; 181 Faculté des Sciences, Université Mohammed V, Rabat, Morocco; 182 DSM/IRFU (Institut de Recherches sur les Lois Fondamentales de l’Univers), CEA Saclay (Commissariat à l’Energie Atomique et aux Energies Alternatives), Gif-sur-Yvette, France; 183 Santa Cruz Institute for Particle Physics, University of California Santa Cruz, Santa Cruz, CA USA; 184 Department of Physics, University of Washington, Seattle, WA USA; 185 Department of Physics and Astronomy, University of Sheffield, Sheffield, UK; 186 Department of Physics, Shinshu University, Nagano, Japan; 187 Fachbereich Physik, Universität Siegen, Siegen, Germany; 188 Department of Physics, Simon Fraser University, Burnaby, BC Canada; 189 SLAC National Accelerator Laboratory, Stanford, CA USA; 190 Faculty of Mathematics, Physics and Informatics, Comenius University, Bratislava, Slovak Republic; 191 Department of Subnuclear Physics, Institute of Experimental Physics of the Slovak Academy of Sciences, Kosice, Slovak Republic; 192 Department of Physics, University of Cape Town, Cape Town, South Africa; 193 Department of Physics, University of Johannesburg, Johannesburg, South Africa; 194 School of Physics, University of the Witwatersrand, Johannesburg, South Africa; 195 Department of Physics, Stockholm University, Stockholm, Sweden; 196 The Oskar Klein Centre, Stockholm, Sweden; 197 Physics Department, Royal Institute of Technology, Stockholm, Sweden; 198 Departments of Physics and Astronomy and Chemistry, Stony Brook University, Stony Brook, NY USA; 199 Department of Physics and Astronomy, University of Sussex, Brighton, UK; 200 School of Physics, University of Sydney, Sydney, NSW Australia; 201 Institute of Physics, Academia Sinica, Taipei, Taiwan; 202 Department of Physics, Technion: Israel Institute of Technology, Haifa, Israel; 203 Raymond and Beverly Sackler School of Physics and Astronomy, Tel Aviv University, Tel Aviv, Israel; 204 Department of Physics, Aristotle University of Thessaloniki, Thessaloniki, Greece; 205 International Center for Elementary Particle Physics and Department of Physics, The University of Tokyo, Tokyo, Japan; 206 Graduate School of Science and Technology, Tokyo Metropolitan University, Tokyo, Japan; 207 Department of Physics, Tokyo Institute of Technology, Tokyo, Japan; 208 Tomsk State University, Tomsk, Russia; 209 Department of Physics, University of Toronto, Toronto, ON Canada; 210 INFN-TIFPA, Povo, Italy; 211 University of Trento, Trento, Italy; 212 TRIUMF, Vancouver, BC Canada; 213 Department of Physics and Astronomy, York University, Toronto, ON Canada; 214 Faculty of Pure and Applied Sciences, and Center for Integrated Research in Fundamental Science and Engineering, University of Tsukuba, Tsukuba, Japan; 215 Department of Physics and Astronomy, Tufts University, Medford, MA USA; 216 Department of Physics and Astronomy, University of California Irvine, Irvine, CA USA; 217 INFN Gruppo Collegato di Udine, Sezione di Trieste, Udine, Italy; 218 ICTP, Trieste, Italy; 219 Dipartimento di Chimica Fisica e Ambiente, Università di Udine, Udine, Italy; 220 Department of Physics and Astronomy, University of Uppsala, Uppsala, Sweden; 221 Department of Physics, University of Illinois, Urbana, IL USA; 222 Instituto de Fisica Corpuscular (IFIC) and Departamento de Fisica Atomica, Molecular y Nuclear and Departamento de Ingeniería Electrónica and Instituto de Microelectrónica de Barcelona (IMB-CNM), University of Valencia and CSIC, Valencia, Spain; 223 Department of Physics, University of British Columbia, Vancouver, BC Canada; 224 Department of Physics and Astronomy, University of Victoria, Victoria, BC Canada; 225 Department of Physics, University of Warwick, Coventry, UK; 226 Waseda University, Tokyo, Japan; 227 Department of Particle Physics, The Weizmann Institute of Science, Rehovot, Israel; 228 Department of Physics, University of Wisconsin, Madison, WI USA; 229 Fakultät für Physik und Astronomie, Julius-Maximilians-Universität, Würzburg, Germany; 230 Fakultät für Mathematik und Naturwissenschaften, Fachgruppe Physik, Bergische Universität Wuppertal, Wuppertal, Germany; 231 Department of Physics, Yale University, New Haven, CT USA; 232 Yerevan Physics Institute, Yerevan, Armenia; 233 Centre de Calcul de l’Institut National de Physique Nucléaire et de Physique des Particules (IN2P3), Villeurbanne, France; 234 CERN, 1211 Geneva 23, Switzerland

## Abstract

Two searches for new phenomena in final states containing a same-flavour opposite-sign lepton (electron or muon) pair, jets, and large missing transverse momentum are presented. These searches make use of proton–proton collision data, collected during 2015 and 2016 at a centre-of-mass energy $\sqrt{s}=13$ $\text {TeV}$ by the ATLAS detector at the large hadron collider, which correspond to an integrated luminosity of $14.7~\mathrm {fb}^{-1}$. Both searches target the pair production of supersymmetric particles, squarks or gluinos, which decay to final states containing a same-flavour opposite-sign lepton pair via one of two mechanisms: a leptonically decaying *Z* boson in the final state, leading to a peak in the dilepton invariant-mass distribution around the *Z* boson mass; and decays of neutralinos (e.g. $\tilde{\chi }_2^0 \rightarrow \ell ^+\ell ^- \tilde{\chi }_1^0$), yielding a kinematic endpoint in the dilepton invariant-mass spectrum. The data are found to be consistent with the Standard Model expectation. Results are interpreted in simplified models of gluino-pair (squark-pair) production, and provide sensitivity to gluinos (squarks) with masses as large as 1.70 $\text {TeV}$ (980 $\text {GeV}$).

## Introduction

Supersymmetry (SUSY) [1–7] is an extension of the Standard Model (SM) that introduces partner particles (called *sparticles*) that differ by half a unit of spin from their SM counterparts. The squarks ($\tilde{q}$) and sleptons ($\tilde{\ell }$) are the scalar partners of the quarks and leptons, respectively, and the gluinos ($\tilde{g}$) are the fermionic partners of the gluons. The charginos (${\tilde{\chi }}_{i}^{\pm }$) and neutralinos (${\tilde{\chi }}_{i}^{0}$) are the mass eigenstates (where the index *i* is ordered from the lightest to the heaviest) formed from the linear superpositions of the SUSY partners of the Higgs bosons (higgsinos) and electroweak gauge bosons.

If the masses of the gluino, higgsinos, and top squarks are close to the $\text {TeV}$ scale, SUSY may offer a solution to the SM hierarchy problem [8–11]. In this case, strongly interacting sparticles should be produced at a high enough rate to be detected by the experiments at the large hadron collider (LHC). For models with R-parity conservation [12], such sparticles would be pair-produced and are expected to decay into jets, perhaps leptons, and the lightest stable SUSY particle (LSP). The LSP is assumed to be only weakly interacting and therefore escapes the detector, resulting in events with potentially large missing transverse momentum (${\varvec{p}}_{\mathrm {T}}^\mathrm {miss}$, with magnitude $E_{\text {T}}^{\text {miss}}$). In such a scenario the LSP could be a dark-matter candidate [13, 14].

Final states containing pairs of leptons may arise from the cascade decays of squarks and gluinos via several mechanisms. In this paper, two search channels are considered that target scenarios with same-flavour (SF) opposite-sign (OS) lepton (electron or muon) pairs. The first channel requires a lepton pair with an invariant mass $m_{\ell \ell }$ that is consistent with the *Z* boson mass $m_Z$ (“on-shell *Z*” channel), while the second channel considers all SFOS lepton pairs (“edge” channel). The presence of two leptons in the final state suppresses large SM backgrounds from, e.g., QCD multijet and $W+\mathrm {jets}$ production, providing a clean environment in which to search for new physics. As discussed further below, in such events the distribution of dilepton mass $m_{\ell \ell }$ may be used to characterise the nature of the SUSY particle decay and constrain mass differences between SUSY particles.

The SFOS lepton pairs may be produced in the decay $\tilde{\chi }_{2}^{0} \rightarrow \ell ^{+}\ell ^{-} \tilde{\chi }_{1}^{0}$ (or, in models of generalised gauge mediation with a gravitino LSP [15–17], via $\tilde{\chi }_{1}^{0} \rightarrow \ell ^{+}\ell ^{-} \tilde{G}$). The properties of the $\tilde{\chi }_2^0$ decay depend on the mass difference $\Delta m_\chi \equiv m_{\tilde{\chi }_{2}^{0}} - m_{\tilde{\chi }_{1}^{0}}$, the mixing of the charginos and neutralinos, and on whether there are additional sparticles with masses less than $m_{\tilde{\chi }_{2}^{0}}$ that may be produced in the decay of the $\tilde{\chi }_2^0$ particle. For $\Delta m_\chi >m_Z$, SFOS lepton pairs may be produced in the decay $\tilde{\chi }_{2}^{0} \rightarrow Z \tilde{\chi }_{1}^{0} \rightarrow \ell ^{+}\ell ^{-} \tilde{\chi }_{1}^{0}$, leading to a peak in the invariant-mass distribution near $m_{\ell \ell } \approx m_Z$. Such models are the target of the on-shell *Z* search. For $\Delta m_\chi < m_Z$, the decay $\tilde{\chi }_{2}^{0} \rightarrow Z^* \tilde{\chi }_{1}^{0} \rightarrow \ell ^{+}\ell ^{-} \tilde{\chi }_{1}^{0}$ leads to a rising $m_{\ell \ell }$ distribution that is truncated at a kinematic endpoint, whose position is given by $m_{\ell \ell }^{\text {max}}=\Delta m_\chi < m_Z$, below the *Z* boson mass peak. If there are sleptons with masses less than $m_{\tilde{\chi }_{2}^{0}}$, the $\tilde{\chi }_2^0$ particle may decay as $\tilde{\chi }_{2}^{0} \rightarrow \tilde{\ell }^{\pm }\ell ^{\mp } \rightarrow \ell ^{+}\ell ^{-} \tilde{\chi }_{1}^{0}$, also leading to a kinematic endpoint but with a different shape and $m_{\ell \ell }$ endpoint position, given by $m_{\ell \ell }^{\mathrm {max}} = \sqrt{ (m^2_{\tilde{\chi }_2^0}-m^2_{\tilde{\ell }})(m^2_{\tilde{\ell }}-m^2_{\tilde{\chi }_1^0}) / m^2_{\tilde{\ell }}}$, which may occur below, on, or above the *Z* boson mass peak. The latter two scenarios are targeted by the “edge” search channel, which considers the full $m_{\ell \ell }$ range.

This paper reports on a search for SUSY in the same-flavour dilepton final state with $14.7~\mathrm {fb}^{-1}$ of *pp* collision data at $\sqrt{s}=13$ $\text {TeV}$ recorded in 2015 and 2016 by the ATLAS detector at the LHC. Searches for SUSY in the $Z+\mathrm {jets}+E_{\text {T}}^{\text {miss}}$ final state have previously been performed at $\sqrt{s}=8$ $\text {TeV}$ by the CMS [18, 19] and ATLAS [20] collaborations using Run-1 LHC data. In the ATLAS analysis performed with 20.3 fb$^{-1}$ of $\sqrt{s}=8$ $\text {TeV}$ data reported in Ref. [20], an excess of events above the SM background with a significance of 3.0 standard deviations was observed. The event selection criteria for the on-shell *Z* search in this paper are almost identical, differing only in the details of the analysis object definitions and missing transverse momentum. CMS performed a search with $\sqrt{s}=13$ $\text {TeV}$ data in a similar kinematic region but did not observe evidence to corroborate this excess [21].

Searches for an edge in the $m_{\ell \ell }$ distribution in events with $2\ell +\mathrm {jets}+E_{\text {T}}^{\text {miss}}$ have been performed by the CMS [19, 22] and ATLAS [20] collaborations. In Ref. [19], CMS reported an excess above the SM prediction with a significance of 2.6 standard deviations. In a similar search region, however, the Run-1 ATLAS analysis [20] and Run-2 CMS analysis [21] observed results consistent with the SM prediction.

## ATLAS detector

The ATLAS detector [23] is a general-purpose detector with almost $4\pi$ coverage in solid angle.^1^ The detector comprises an inner tracking detector, a system of calorimeters, and a muon spectrometer.

The inner tracking detector (ID) is immersed in a 2 T magnetic field provided by a superconducting solenoid and allows charged-particle tracking out to $|\eta |=2.5$. It includes silicon-pixel and silicon-strip tracking detectors inside a straw-tube tracking detector. In 2015 the detector received a new innermost layer of silicon pixels, which improves the track impact parameter resolution by almost a factor of two in both the transverse and longitudinal directions [24].

High-granularity electromagnetic and hadronic calorimeters cover the region $|\eta |<4.9$. All the electromagnetic calorimeters, as well as the endcap and forward hadronic calorimeters, are sampling calorimeters with liquid argon as the active medium and lead, copper, or tungsten as the absorber. The central hadronic calorimeter is a sampling calorimeter with scintillator tiles as the active medium and steel as the absorber.

The muon spectrometer uses several detector technologies to provide precision tracking out to $|\eta |=2.7$ and triggering in $|\eta |<2.4$, making use of a system of three toroidal magnets.

The ATLAS detector incorporates a two-level trigger system, with the first level implemented in custom hardware and the second level implemented in software. This trigger system selects events of interest at an output rate of about 1 kHz.

## SUSY signal models

**Fig. 1:**
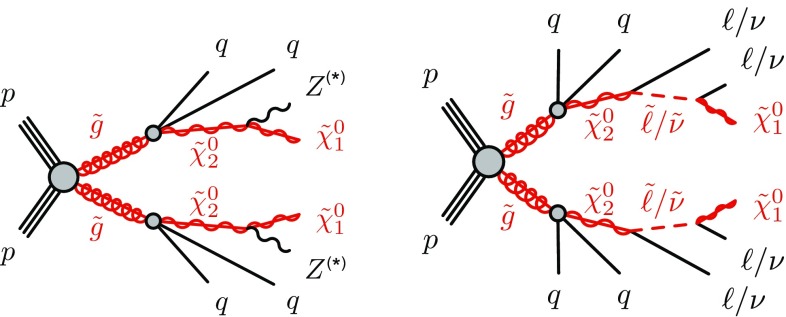
Example decay topologies for two of the simplified models considered, involving gluino-pair production, with the gluinos following an effective three-body decay for $\tilde{g}\rightarrow q \bar{q} \tilde{\chi }_2^0$, with $\tilde{\chi }_2^0\rightarrow Z^{(*)} \tilde{\chi }_1^0$ (*left*) and $\tilde{\chi }_{2}^{0} \rightarrow \tilde{\ell }^{\mp }\ell ^{\pm } / \tilde{\nu }\nu$ (*right*). For simplicity, no distinction is made between particles and antiparticles

SUSY-inspired simplified models are considered as signal scenarios for these analyses. In all of these models, squarks or gluinos are directly pair-produced, decaying via an intermediate neutralino, $\tilde{\chi }_2^0$, into the LSP ($\tilde{\chi }_1^0$). All sparticles not directly involved in the decay chains considered are effectively decoupled. Two example decay topologies are shown in Fig. 1. For all models with gluino-pair production, a three-body decay for $\tilde{g}\rightarrow q \bar{q} \tilde{\chi }_2^0$ is used. Signal models are generated in a grid over a two-dimensional space, varying the gluino or squark mass and the mass of either the $\tilde{\chi }_2^0$ or the $\tilde{\chi }_1^0$.

Three models, one with squark-pair production and two with gluino-pair production, which result exclusively in events with two on-shell *Z* bosons in the final state are considered for the on-shell search. For two of these models, signal mass points are generated across the $\tilde{g}$–$\tilde{\chi }_2^0$ (or $\tilde{q}$–$\tilde{\chi }_2^0$) plane. These models are produced following the decays $\tilde{g}\rightarrow q \bar{q} \tilde{\chi }_2^0$ or $\tilde{q}\rightarrow q \tilde{\chi }_2^0$, with the $\tilde{\chi }_1^0$ (LSP) mass set to 1 $\text {GeV}$, inspired by SUSY scenarios with a low-mass LSP (e.g. generalised gauge mediation). These two models are referred to here as the $\tilde{g}$–$\tilde{\chi }_2^0$ on-shell and $\tilde{q}$–$\tilde{\chi }_2^0$ on-shell grids, respectively, and are summarised in Table 1. The third model is based on MSSM-inspired topologies [25–27] with potentially higher mass LSPs. Signal points are generated across the $\tilde{g}$–$\tilde{\chi }_1^0$ plane, and this model is thus referred to as the $\tilde{g}$–$\tilde{\chi }_1^0$ on-shell grid. In this case the $\tilde{\chi }_2^0$ mass is set to be 100 $\text {GeV}$ above the $\tilde{\chi }_1^0$ mass, which in many models maximises the branching fraction of the $\tilde{\chi }_2^0$ decay to *Z* bosons. For the two models with gluino-pair production, since the gluino coupling to $q\tilde{q}$ is flavour independent and the corresponding flavours of squarks are assumed to be mass degenerate, the branching fractions for $q=u,d,c,s$ are each 25%. Other ATLAS searches are dedicated to final states with two leptons and heavy flavour jets [28, 29]. For the model involving squark-pair production, the superpartners of the *u*, *d*, *c* and *s* quarks have the same mass, with the superpartners of the *b* and *t* quarks being decoupled.

**Table 1:** Summary of the simplified signal model topologies used in this paper. Here *x* and *y* denote the *x*–*y* plane across which the signal model masses are varied to construct the signal grid. For the slepton model, the masses of the superpartners of the left-handed leptons are given by $[m(\tilde{\chi }_2^0)+m(\tilde{\chi }_1^0)]/2$, while the superpartners of the right-handed leptons are decoupled

Model	Production mode	Quark flavours	$m(\tilde{g})/m(\tilde{q})$	$m(\tilde{\chi }^{0}_{2})$	$m(\tilde{\chi }^{0}_{1})$
$\tilde{g}$–$\tilde{\chi }_2^0$ on-shell	$\tilde{g}\tilde{g}$	*u*, *d*, *c*, *s*	*x*	*y*	1 $\text {GeV}$
$\tilde{g}$–$\tilde{\chi }_1^0$ on-shell	$\tilde{g}\tilde{g}$	*u*, *d*, *c*, *s*	*x*	$m(\tilde{\chi }^0_1)+100$ $\text {GeV}$	*y*
$\tilde{q}$–$\tilde{\chi }_2^0$ on-shell	$\tilde{q}\tilde{q}$	*u*, *d*, *c*, *s*	*x*	*y*	1 $\text {GeV}$
$Z^{(*)}$	$\tilde{g}\tilde{g}$	*u*, *d*, *c*, *s*, *b*	*x*	$[m(\tilde{g})+m(\tilde{\chi }_1^0)]/2$	*y*
slepton	$\tilde{g}\tilde{g}$	*u*, *d*, *c*, *s*, *b*	*x*	$[m(\tilde{g})+m(\tilde{\chi }_1^0)]/2$	*y*

The edge search considers two scenarios, both of which involve the direct pair production of gluinos and differ by the decay mode of the $\tilde{\chi }_{2}^{0}$. These signal models are also summarised in Table 1. In the $Z^{(*)}$ model the $\tilde{\chi }_{2}^{0}$ decays as $\tilde{\chi }_{2}^{0} \rightarrow Z^{(*)}\tilde{\chi }_{1}^{0}$. For $\Delta m_\chi = m(\tilde{\chi }_2^0) - m(\tilde{\chi }_1^0) > m_Z$, the *Z* boson is on-shell, leading to a peak in the $m_{\ell \ell }$ distribution at $m_Z$, while for $\Delta m_\chi < m_Z$, the *Z* boson is off-shell, leading to an edge in the dilepton mass distribution with a position below $m_Z$. The slepton model assumes that the sleptons are lighter than the $\tilde{\chi }_{2}^{0}$, which decays as $\tilde{\chi }_{2}^{0} \rightarrow \tilde{\ell }^{\mp }\ell ^{\pm }$ with $\tilde{\ell } \rightarrow \ell \tilde{\chi }_{1}^{0}$ or as $\tilde{\chi }_{2}^{0} \rightarrow \tilde{\nu }\nu$ with $\tilde{\nu } \rightarrow \nu \tilde{\chi }_{1}^{0}$, each with a branching fraction of 50%, where $\tilde{\ell }=\tilde{e},\tilde{\mu },\tilde{\tau }$ and $\tilde{\nu }=\tilde{\nu _e},\tilde{\nu _\mu },\tilde{\nu _\tau }$. The endpoint position can occur at any mass, highlighting the need to search over the full dilepton mass distribution. The gluino decays as $\tilde{g}\rightarrow q \bar{q} \tilde{\chi }_2^0$, and both models have equal branching fractions for $q=u,d,c,s,b$. The $\tilde{\chi }_2^0$ mass is set to the average of the gluino and $\tilde{\chi }_1^0$ masses. For the slepton model, the masses of the superpartners of the left-handed leptons are set as the average of the $\tilde{\chi }_2^0$ and $\tilde{\chi }_1^0$ masses, while the superpartners of the right-handed leptons are decoupled. The three slepton flavours are mass-degenerate. In both these models the $\tilde{g}$ and $\tilde{\chi }_1^0$ masses are free parameters that are varied to produce the two-dimensional signal grid. The mass splittings are chosen to maximise the differences between these simplified models and other models with only one intermediate particle between the gluino and the LSP [30].

## Data and Monte Carlo samples

The data used in this analysis were collected by ATLAS during 2015 and 2016, with a mean number of additional *pp* interactions per bunch crossing (*pile-up*) of approximately 14 in 2015 and 21 in 2016, and a centre-of-mass collision energy of 13 $\text {TeV}$. Following requirements based on beam and detector conditions and data quality, the data set corresponds to an integrated luminosity of $14.7~\mathrm {fb}^{-1}$. The uncertainty in the combined 2015 and 2016 integrated luminosity is $\pm 2.9$%. It is derived, following a methodology similar to that detailed in Refs. [31] and [32], from a preliminary calibration of the luminosity scale using *x*–*y* beam-separation scans performed in August 2015 and May 2016.

Data events are collected using a combination of single-lepton and dilepton triggers [33], in order to maximise the signal acceptance. The dielectron, dimuon, and electron–muon triggers have leading-lepton $p_{\text {T}}$ thresholds in the range 12–24 $\text {GeV}$. Additional single-electron (single-muon) triggers are also used, with trigger $p_{\text {T}}$ thresholds of 60 (50) $\text {GeV}$, to increase the trigger efficiency for models with high-$p_{\text {T}}$ leptons. Events are required to contain at least two selected leptons with $p_{\text {T}} >25$ $\text {GeV}$, making the selection fully efficient with respect to the trigger $p_{\text {T}}$ thresholds.

An additional control sample of events containing photons is collected using a set of single-photon triggers with $p_{\text {T}}$ thresholds in the range 20–140 $\text {GeV}$. All triggers except for the one with threshold $p_{\text {T}} =120$ $\text {GeV}$ in 2015, or the one with $p_{\text {T}} =140$ $\text {GeV}$ in 2016, are prescaled. Events are required to contain a selected photon with $p_{\text {T}} >37$ $\text {GeV}$, so that they are selected efficiently by the lowest available $p_{\text {T}}$ trigger in 2015, which had a threshold of $p_{\text {T}} ^{\gamma }=35$ $\text {GeV}$.

Simulated event samples are used to aid in the estimation of SM backgrounds, validate the analysis techniques, optimise the event selection, and provide predictions for SUSY signal processes. All SM background samples used are listed in Table 2, along with the parton distribution function (PDF) set, the configuration of underlying-event and hadronisation parameters (underlying-event tune) and the cross-section calculation order in $\alpha _{\text {S}}$ used to normalise the event yields for these samples.

Samples simulated using MG5_aMC@NLO v2.2.2 [34], interfaced with Pythia 8.186 [35] with the A14 underlying-event tune [36] to simulate the parton shower and hadronisation, are generated at leading order in $\alpha _{\text {S}}$ (LO) with the NNPDF23LO PDF set [37]. For samples generated using Powheg Box V2 [38–40], Pythia 6.428 [41] is used to simulate the parton shower, hadronisation, and the underlying event. The CTEQ6L1 PDF set is used with the corresponding Perugia2012 [42] tune. In the case of both the MG5_aMC@NLO and Powheg samples, the EvtGen v1.2.0 program [43] is used for properties of the bottom and charm hadron decays. Sherpa 2.1.1 [44] simulated samples use the CT10 PDF set with Sherpa’s own internal parton shower [45] and hadronisation methods, as well as the Sherpa default underlying-event tune. Diboson processes with four charged leptons, three charged leptons and a neutrino or two charged leptons and two neutrinos are simulated using the Sherpa 2.1.1 generator. Matrix elements contain all diagrams with four electroweak vertices. They are calculated for up to one ($4\ell$, $2\ell +2\nu$) or zero ($3\ell +1\nu$) partons at next-to-leading order in $\alpha _{\text {S}}$ (NLO) and up to three partons at LO using the Comix [46] and OpenLoops [47] matrix element generators and merged with the Sherpa parton shower using the ME+PS@NLO prescription [48]. For the $Z/\gamma ^{*}+\text {jets}$ background, Sherpa 2.1.1 is used to generate a sample with up to two additional partons at NLO and up to four at LO. For Monte Carlo (MC) closure studies, $\gamma +\text {jets}$ events are generated at LO with up to four additional partons using Sherpa 2.1.1. Additional MC simulation samples of events with a leptonically decaying vector boson and photon ($V\gamma$, where $V=W,Z$) are generated at LO using Sherpa 2.1.1. Matrix elements including all diagrams with three electroweak couplings are calculated with up to three partons. These samples are used to estimate backgrounds with real $E_{\text {T}}^{\text {miss}}$ in $\gamma +\text {jets}$ event samples.

The SUSY signal samples are produced at LO using MG5_aMC@NLO with the NNPDF2.3LO PDF set, interfaced with Pythia 8.186. The scale parameter for CKKW-L matching [49, 50] is set at a quarter of the mass of the gluino. Up to one additional parton is included in the matrix element calculation. The underlying event is modelled using the A14 tune for all signal samples, and EvtGen is adopted to describe the properties of bottom and charm hadron decays. Signal cross sections are calculated at NLO in $\alpha _{\text {S}}$. This includes the resummation of soft gluon emission at next-to-leading-logarithm accuracy (NLO+NLL) [51–55].

All of the SM background MC samples are subject to a full ATLAS detector simulation [56] using GEANT4 [57]. A fast simulation [56], which uses a combination of a parameterisation of the response of the ATLAS electromagnetic and hadronic calorimeters and GEANT4, is used in the case of signal MC samples. This fast simulation is validated by comparing a few chosen signal samples to some fully simulated points. Minimum-bias interactions are generated and overlaid on the hard-scattering process to simulate the effect of multiple *pp* interactions occurring during the same (in-time) or a nearby (out-of-time) bunch-crossing (pile-up). These are produced using Pythia 8.186 with the A2 tune [58] and MSTW 2008 PDF set [59]. The pile-up distribution in MC samples is simulated to match that in data during 2015 and 2016 *pp* data-taking.

**Table 2:** Simulated background event samples used in this analysis with the corresponding matrix element and parton shower generators, cross-section order in $\alpha _{\text {S}}$ used to normalise the event yield, underlying-event tune and PDF set

Physics process	Generator	Parton shower	Cross section	Tune	PDF set
$t\bar{t}+W$ and $t\bar{t}+Z$ [60, 61]	MG5_aMC@NLO	Pythia 8.186	NLO [62, 63]	A14	NNPDF23LO
$t\bar{t}+WW$ [60]	MG5_aMC@NLO	Pythia 8.186	LO [34]	A14	NNPDF23LO
$t\bar{t}$ [64]	Powheg Box v2 r3026	Pythia 6.428	NNLO+NNLL [65, 66]	Perugia2012	NLO CT10
Single-top (*Wt*) [64]	Powheg Box v2 r2856	Pythia 6.428	Approx. NNLO [67]	Perugia2012	NLO CT10
*WW*, *WZ* and *ZZ* [68]	Sherpa 2.1.1	Sherpa 2.1.1	NLO [69, 70]	Sherpa default	NLO CT10
$Z/\gamma ^{*}(\rightarrow \ell \ell )$ + jets [71]	Sherpa 2.1.1	Sherpa 2.1.1	NNLO [72, 73]	Sherpa default	NLO CT10
$\gamma +\text {jets}$	Sherpa 2.1.1	Sherpa 2.1.1	LO [44]	Sherpa default	NLO CT10
$V(=W,Z)\gamma$	Sherpa 2.1.1	Sherpa 2.1.1	LO [44]	Sherpa default	NLO CT10

## Analysis object identification and selection

All analysis objects are categorised as either “baseline” or “signal” based on various quality and kinematic requirements. *Baseline* objects are used in the calculation of missing transverse momentum and to disambiguate between the analysis objects in the event, while the jets and leptons entering the final analysis selection must pass more stringent *signal* requirements. The selection criteria for both the baseline and signal objects differ from the requirements used in the Run-1 ATLAS $Z+\mathrm {jets}+E_{\text {T}}^{\text {miss}}$ search reported in Ref. [20], owing to the new silicon-pixel tracking layer and significant changes to the reconstruction software since 2012 data-taking. In particular, improvements in the lepton identification criteria have reduced the background due to hadrons misidentified as electrons. The primary vertex in each event is defined as the reconstructed vertex [74] with the highest $\sum p_{\text {T}}^2$, where the summation includes all particle tracks with $p_{\text {T}} >400$ $\text {MeV}$ associated with the vertex.

Electron candidates are reconstructed from energy clusters in the electromagnetic calorimeter matched to ID tracks. Baseline electrons are required to have transverse energy $E_{\text {T}}>10$ $\text {GeV}$, satisfy the “loose likelihood” criteria described in Ref. [75] and reside within the region $|\eta |<2.47$. Signal electrons are further required to have $p_{\text {T}} >25$ $\text {GeV}$, satisfy the “medium likelihood” criteria of Ref. [75], and be consistent with originating from the primary vertex. The signal electrons must originate from within $|z_0\sin \theta | = 0.5$ mm of the primary vertex along the direction of the beamline.^2^ The transverse-plane distance of closest approach of the electron to the beamline, divided by the corresponding uncertainty, must be $|d_0/\sigma _{d_0}|<5$. These electrons must also be isolated with respect to other objects in the event, according to a $p_{\text {T}}$-dependent isolation requirement. The isolation uses calorimeter- and track-based information to obtain 95% efficiency at $p_{\text {T}} =25$ $\text {GeV}$, rising to 99% efficiency at $p_{\text {T}} =60$ $\text {GeV}$.

Baseline muons are reconstructed from either ID tracks matched to muon segments (collections of hits in a single muon spectrometer layer) or combined tracks formed from the ID and muon spectrometer [76]. They must satisfy the “medium” selection criteria described in Ref. [76], and to satisfy $p_{\text {T}}>10$ $\text {GeV}$ and $|\eta |<2.5$. Signal muon candidates are further required to have $p_{\text {T}} >25$ $\text {GeV}$, be isolated, and have $|z_0\sin \theta | < 0.5$ mm and $|d_0/\sigma _{d_0}|<3$. Calorimeter- and track-based isolation criteria are used to obtain 95% efficiency at $p_{\text {T}} =25$ $\text {GeV}$, rising to 99% efficiency at $p_{\text {T}} =80$ $\text {GeV}$ [76]. Further, the relative uncertainties in the *q* / *p* of each of the ID track alone and muon spectrometer track alone are required to be less than 80% of the uncertainty in the *q* / *p* of the combined track. This reduces the already low rate of grossly mismeasured muons. The combined isolation and identification efficiency for single leptons, after the trigger requirements, is about 70% (80%) for electrons (muons) with $p_{\text {T}} \sim 25$ $\text {GeV}$, rising to about 90% for $p_{\text {T}} >200$ $\text {GeV}$.

Jets are reconstructed from topological clusters of energy [77] in the calorimeter using the anti-$k_{t}$ algorithm [78, 79] with a radius parameter of 0.4. Calibration corrections are applied to the jets based on a comparison to jets made of stable particles (those with lifetimes $\tau > 0.3 \times 10^{-10}$ s) in the MC simulation. A residual correction is applied to jets in data, based on studies of $p_{\text {T}}$ balance between jets and well-calibrated objects in the MC simulation and data [80, 81]. Baseline jet candidates are required to have $p_{\text {T}} >20$ $\text {GeV}$ and reside within the region $|\eta |<4.5$. Signal jets are further required to satisfy $p_{\text {T}} >30$ $\text {GeV}$ and reside within the region $|\eta |<2.5$. Jets with $p_{\text {T}} <60$ $\text {GeV}$ and $|\eta |<2.4$ must meet additional criteria designed to select jets from the hard-scatter interaction and reject those originating from pile-up. This is enforced by using the jet vertex tagger described in Ref. [82]. Finally, events containing a jet that does not pass specific jet quality requirements are vetoed from the analysis selection in order to remove events impacted by detector noise and non-collision backgrounds [83, 84]. The MV2c10 boosted decision tree algorithm [85, 86] identifies jets with $|\eta |<2.5$ containing *b*-hadrons (*b*-jets) based on quantities such as the impact parameters of associated tracks and any reconstructed secondary vertices. A selection that provides 77% efficiency for tagging *b*-jets in simulated $t\bar{t}$ events is used. These tagged jets are called *b*-tagged jets.

Photon candidates must satisfy “tight” selection criteria described in Ref. [87], have $p_{\text {T}} >25$ $\text {GeV}$ and reside within the region $|\eta |<2.37$, excluding the transition region $1.37<|\eta |<1.6$ where there is a discontinuity in the calorimeter. Signal photons are further required to have $p_{\text {T}} >37$ $\text {GeV}$ and to be isolated from other objects in the event, using $p_{\text {T}}$-dependent requirements on both track- and calorimeter-based isolation.

To avoid the duplication of analysis objects in more than one baseline selection, an overlap removal procedure is applied. Any baseline jet within $\Delta R=0.2$ of a baseline electron is removed, unless the jet is *b*-tagged, in which case the electron is identified as originating from a heavy-flavour decay and is removed instead. Remaining electrons residing within $\Delta R=0.4$ of a baseline jet are then removed from the event. Subsequently, any baseline muon residing within $\Delta R=0.2$ of a remaining baseline *b*-tagged jet is discarded. If such a jet is not *b*-tagged then the jet is removed instead. Any remaining muon found within $\mathrm { min } (0.04+(10~\text {GeV})/p_{\text {T}},0.4)$ of a jet is also discarded. This stage of the overlap removal procedure differs from that used in Ref. [20]. It was improved to retain muons near jet candidates mostly containing calorimeter energy from final-state radiation from muons, while still rejecting muons from heavy-flavour decays. Finally, to remove electron candidates originating from muon bremsstrahlung, any baseline electron within $\Delta R=0.01$ of any remaining baseline muon is removed from the event. Photons are removed if they reside within $\Delta R=0.4$ of a baseline electron, and any jet within $\Delta R=0.4$ of any remaining photon is discarded.

The $E_{\text {T}}^{\text {miss}}$ is defined as the magnitude of the negative vector sum, ${\varvec{p}}_{\mathrm {T}}^\mathrm {miss}$, of the transverse momenta of all baseline electrons, muons, jets, and photons [88, 89]. Low-momentum contributions from particle tracks from the primary vertex that are not associated with reconstructed analysis objects are included in the calculation of $E_{\text {T}}^{\text {miss}}$. This contribution to the $E_{\text {T}}^{\text {miss}}$ is referred to as the “soft term”.

Models with large hadronic activity are targeted by placing additional requirements on the quantity $H_\text {T}$, defined as the scalar sum of the $p_{\text {T}}$ values of all signal jets, or on $H_{\mathrm {T}}^{\mathrm {incl}}$, the scalar sum of the $p_{\text {T}}$ values of all signal jets and the two leptons with largest $p_{\text {T}}$.

All MC samples have correction factors applied to take into account small differences between data and MC simulation in identification, reconstruction and trigger efficiencies for leptons. The $p_{\text {T}}$ values of leptons in MC samples are additionally smeared to match the momentum resolution in data.

## Event selection

For each search channel, signal regions (SRs) are designed to target events from specific SUSY signal models. Control regions (CRs) are defined to be depleted in SUSY signal events and enriched in specific SM backgrounds, and they are used to assist in estimating these backgrounds in the SRs. To validate the background estimation procedures, various validation regions (VRs) are defined to be analogous to the CRs and SRs, but with less stringent requirements than the SRs on $E_{\text {T}}^{\text {miss}}$, $H_{\mathrm {T}}^{\mathrm {incl}}$ or $H_{\text {T}}$. Other VRs with additional requirements on the number of leptons are used to validate the modelling of backgrounds in which more than two leptons are expected.

Events in SRs are required to contain at least two signal leptons (electrons or muons). If more than two signal leptons are present in a given event, the selection process continues based on the two leptons with the highest $p_{\text {T}}$ values in the event.

The selected events must pass at least one of the leptonic triggers. If an event is selected by a dilepton trigger, the two leading, highest $p_{\text {T}}$, leptons must be matched to one of the objects that triggered the event. These leptons must also have $p_{\text {T}}$ higher than the threshold of the trigger in question. For events selected by a single-lepton trigger, at least one of the two leading leptons must be matched to the trigger object in the same way. The leading two leptons in the event must have $p_{\text {T}} >25$ $\text {GeV}$, and form an SFOS pair.

As at least two jets are expected in all signal models studied, selected events are further required to contain at least two signal jets. Furthermore, events in which the azimuthal opening angle between either of the leading two jets and the $E_{\text {T}}^{\text {miss}}$ satisfies $\Delta \phi (\text {jet}_{12},{\varvec{p}}_{\mathrm {T}}^\mathrm {miss})<0.4$ are rejected so as to remove events with $E_{\text {T}}^{\text {miss}}$ from jet mismeasurements. This requirement also suppresses $t\bar{t}$ events in which the top quark, the anti-top quark, or the entire $t\bar{t}$ system has a large Lorentz boost.

**Fig. 2:**
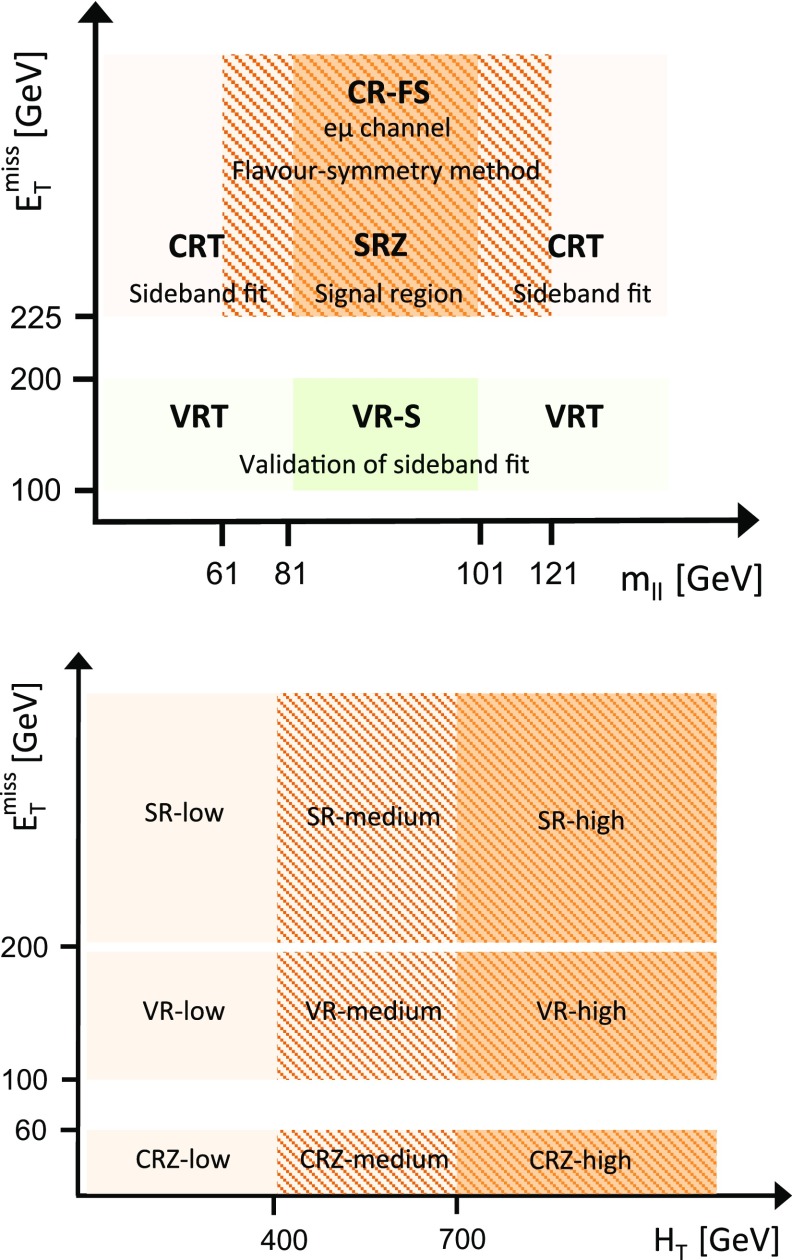
Schematic diagrams of the control (CR), validation (VR) and signal regions (SR) for the on-shell *Z* (*top*) and edge (*bottom*) searches. For the on-shell *Z* search the various regions are shown in the $m_{\ell \ell }$–$E_{\text {T}}^{\text {miss}}$ plane, whereas in the case of the edge search the signal and validation regions are depicted in the $H_{\text {T}}$–$E_{\text {T}}^{\text {miss}}$ plane. The flavour-symmetry and sideband-fit background estimation methods are described further in Sect. 7.1

The various methods used predict the background in the SRs are discussed in Sect. 7. The selection criteria for the CRs, VRs, and SRs are summarised in Tables 3 and 4. The most important of these regions are shown graphically in Fig. 2.

**Table 3:** Overview of all signal (SR), control (CR) and validation regions (VR) used in the on-shell *Z* search. The flavour combination of the dilepton pair is denoted as either “SF” for same-flavour or “DF” for different-flavour. All regions require at least two leptons, unless otherwise indicated. In the case of CR$\gamma$, VR-WZ, VR-ZZ, and VR-3L the number of leptons, rather than a specific flavour configuration, is indicated. More details are given in the text. The main requirements that distinguish the control and validation regions from the signal region are indicated in bold. The kinematic quantities used to define these regions are discussed in the text. The quantity $m_{\text {T}}(\ell _{3},E_{\text {T}}^{\text {miss}})$ indicates the transverse mass formed by the $E_{\text {T}}^{\text {miss}}$ and the lepton which is not assigned to either of the *Z*-decay leptons

On-shell *Z* regions	${{E}_\mathrm{T}^\mathrm{miss}} \mathrm{(GeV)}$	${H}_\mathrm{T}^\mathrm{incl} \mathrm{(GeV)}$	${n}_\mathrm{jets}$	${m_{\ell \ell }} \mathrm{(GeV)}$	SF/DF	${{\Delta {\phi }}}({\mathrm{jet}}_{\mathbf{12}},{ p}_{\mathrm{T}}^\mathrm{miss})$	${{m}_{\mathrm{T}}({\ell }_{{3}},{{E}_{\mathrm{T}}^{\mathrm{miss}}})}$ (GeV)	${{n}_{{b}\mathrm{-jets}}}$
Signal region								
SRZ	>225	>600	$\ge$2	$81< m_{\ell \ell } < 101$	SF	>0.4	–	–
Control regions								
CRZ	$\varvec{<}$ **60**	>600	$\ge$2	$81< m_{\ell \ell } < 101$	SF	>0.4	–	–
CR-FS	>225	>600	$\ge$2	$\varvec{61< m_{\ell \ell } < 121}$	**DF**	>0.4	–	–
CRT	>225	>600	$\ge$2	$\varvec{>}$ **45**, $\varvec{m_{\ell \ell } \notin [81,101]}$	SF	>0.4	–	–
CR$\gamma$	–	>600	$\ge$2	–	${\varvec{0\ell }, \varvec{1\gamma }}$	–	–	–
Validation regions								
VRZ	$\varvec{<}$ **225**	>600	$\ge$2	$81< m_{\ell \ell } < 101$	SF	>0.4	–	–
VRT	**100–200**	>600	$\ge$2	$\varvec{>}$ **45**, $\varvec{m_{\ell \ell } \notin [81,101]}$	SF	>0.4	–	–
VR-S	**100–200**	>600	$\ge$2	$81< m_{\ell \ell } < 101$	SF	>0.4	–	–
VR-FS	**100–200**	>600	$\ge$2	$\varvec{61< m_{\ell \ell } < 121}$	**DF**	>0.4	–	–
VR-WZ	**100–200**	–	–	–	$\varvec{3\ell }$	–	<100	0
VR-ZZ	$\varvec{<}$ **100**	–	–	–	$\varvec{4\ell }$	–	–	0
VR-3L	**60–100**	$\varvec{>}$ **200**	$\ge$2	$81< m_{\ell \ell } < 101$	$\varvec{3\ell }$	>0.4	–	–

**Table 4:** Overview of all signal (SR), control (CR) and validation regions (VR) used in the edge search. The flavour combination of the dilepton pair is denoted as either “SF” for same-flavour or “DF” for different-flavour. The charge combination of the leading lepton pairs are given as “SS” for same-sign or “OS” for opposite-sign. All regions require *at least* two leptons, with the exception of CR-real, which requires *exactly* two leptons, and the three $\gamma$ CRs, which require no leptons and one photon. More details are given in the text. The main requirements that distinguish the control and validation regions from the signal regions are indicated in bold. The kinematic quantities used to define these regions are discussed in the text

Edge regions	${{E}_{\mathrm{T}}^{\mathrm{miss}}}$ (GeV)	${H}_{\mathrm{T}}$ (GeV)	${{n}_{\mathrm{jets}}}$	${m_{\ell \ell }}$ (GeV)	SF/DF	OS/SS	${{\Delta {\phi }}}({\mathrm{jet}}_\mathbf{12},{p}_{\mathrm{T}}^\mathrm{miss})$	${m_{\ell \ell }}\,\,\mathrm{ranges}$
Signal regions								
SR-low	>200	–	$\ge$2	>12	SF	OS	>0.4	9
SR-medium	>200	>400	$\ge$2	>12	SF	OS	>0.4	8
SR-high	>200	>700	$\ge$2	>12	SF	OS	>0.4	7
Control regions								
CRZ-low	$\varvec{<}$ **60**	–	$\ge$2	>12	SF	OS	>0.4	–
CRZ-medium	$\varvec{<}$ **60**	>400	$\ge$2	>12	SF	OS	>0.4	–
CRZ-high	$\varvec{<}$ **60**	>700	$\ge$2	>12	SF	OS	>0.4	–
CR-FS-low	>200	–	$\ge$2	>12	**DF**	OS	>0.4	–
CR-FS-medium	>200	>400	$\ge$2	>12	**DF**	OS	>0.4	–
CR-FS-high	>200	>700	$\ge$2	>12	**DF**	OS	>0.4	–
CR$\gamma$-low	–	–	$\ge$2	–	${\varvec{0\ell , 1\gamma }}$	–	–	–
CR$\gamma$-medium	–	>400	$\ge$2	–	${\varvec{0\ell ,1\gamma }}$	–	–	–
CR$\gamma$-high	–	>700	$\ge$2	–	${\varvec{0\ell , 1\gamma }}$	–	–	–
CR-real	–	$\varvec{>}$ **200**	$\ge$2	**81–101**	$2\ell$ SF	OS	–	–
CR-fake	$\varvec{<}$ **125**	–	–	${\varvec{\in }[\mathbf{12},\varvec{\infty }]}$ ${\varvec{\notin }{} \mathbf{[81,101](SF)}}$	$\varvec{2\ell }$ **SF/DF**	**SS**	–	–
Validation regions								
VR-low	**100–200**	–	$\ge 2$	$>12$	SF	OS	>0.4	–
VR-medium	**100–200**	>400	$\ge$2	>12	SF	OS	>0.4	–
VR-high	**100–200**	>700	$\ge$2	$>12$	SF	OS	>0.4	–
VR-fake	$\varvec{>}$ **50**	–	$\ge$2	${\varvec{\in }[\mathbf{12},\varvec{\infty }],}$ ${\varvec{\notin }{} \mathbf{[81,101](SF)}}$	**SF/DF**	**SS**	–	–

For the on-shell *Z* search, the leading-lepton $p_{\text {T}}$ threshold is raised to 50 $\text {GeV}$ to increase the sensitivity to signal models with final-state *Z* bosons. This is an increased leading-lepton $p_{\text {T}}$ threshold relative to Ref. [20] and is found to better reject fake-lepton candidates from misidentified jets, photon conversions and *b*-hadron decays, while retaining high efficiency for signal events, which tend to produce boosted *Z* bosons. To select events containing a leptonically decaying *Z* boson, the invariant mass of the dilepton system is required to be $81<m_{\ell \ell }<101$ $\text {GeV}$. In the CRs and VRs that use the *Z* mass sidebands, only events with $m_{\ell \ell }>45$ $\text {GeV}$ are used to reject the lower $m_{\ell \ell }$ region dominated by Drell–Yan (DY) production. In Ref. [20] an “on-*Z*” SR, denoted SRZ, is defined requiring $E_{\text {T}}^{\text {miss}} >225$ $\text {GeV}$ and $H_{\mathrm {T}}^{\mathrm {incl}}> 600$ $\text {GeV}$. The region is motivated by SUSY signals with high gluino or squark mass and high jet activity. Since *b*-jets are not always expected in the simplified models considered here, no requirement is placed on *b*-tagged jet multiplicity ($n_{b\mathrm {-jets}}$) so as to be as inclusive as possible and to be consistent with Ref. [20]. Dedicated CRs are defined, with selection criteria similar to those of SRZ, to estimate the contribution from the dominant SM backgrounds in SRZ. These CRs are discussed in more detail in Sect. 7.

The edge selection requires at least two leptons with $p_{\text {T}} >25$ $\text {GeV}$. The search is performed across the full $m_{\ell \ell }$ spectrum, with the exception of the region with $m_{\ell \ell }<12$ GeV, which is vetoed to reject low-mass DY events and the $J/\psi$ and $\Upsilon$ resonances. Three regions are defined to target signal models with low, medium and high values of $\Delta m_{\tilde{g}} = m(\tilde{g}) - m(\tilde{\chi }^0_1)$, denoted SR-low, SR-medium, and SR-high, respectively. All these regions require $E_{\text {T}}^{\text {miss}} >200$ $\text {GeV}$. SR-medium and SR-high also include the requirements $H_{\text {T}} >400$ $\text {GeV}$ and $H_{\text {T}} >700$ $\text {GeV}$, respectively, to further isolate high-$\Delta m_{\tilde{g}}$ events. Here the leptons are not included in the $H_{\text {T}}$ definition to avoid introducing any bias in the $m_{\ell \ell }$ distribution. Events selected in SR-low, SR-medium and SR-high are further grouped into non-orthogonal $m_{\ell \ell }$ windows, which represent the search regions used in the edge analysis. The dilepton mass ranges of these are chosen to maximise sensitivity to the targeted signal models, with the window boundaries being motivated by the dilepton mass endpoints of generated signal points. In total, 24 $m_{\ell \ell }$ windows are defined by selecting ranges with the best expected sensitivity to signal models. Of these windows, nine are in SR-low, eight are in SR-medium and seven are in SR-high. Details of the $m_{\ell \ell }$ definitions in these regions are given along with the results in Sect. 9. Models without light sleptons are targeted by windows with $m_{\ell \ell }<60$ $\text {GeV}$ or $m_{\ell \ell }<80$ $\text {GeV}$ for $\Delta m_\chi < m_Z$ leading to off-shell *Z* bosons, and by the window with $81<m_{\ell \ell }<101$ $\text {GeV}$ for $\Delta m_\chi > m_Z$ leading to on-shell *Z* bosons. Models with light sleptons are targeted by the remaining $m_{\ell \ell }$ windows, which cover the full $m_{\ell \ell }$ range. The edge selection and on-shell Z selection are not orthogonal. In particular, SR-medium in the range $81<m_{\ell \ell }<101$ $\text {GeV}$ overlaps significantly with SRZ.

For the combined $ee+\mu \mu$ channels, the typical signal acceptance times efficiency values for the signal models considered in SRZ are 2–8%. They are 8–40%, 3–35%, and 1–35%, inclusively in $m_{\ell \ell }$, for SR-low, SR-medium and SR-high, respectively. The on-shell *Z* and edge analyses are each optimised for different signal models. There are models in which signal contamination in CRs or VRs can become significant. For example, CRT in Table 3 is used to normalise the $t\bar{t}$ MC sample to data as a cross-check in the on-shell *Z* search, but it is a region where the signal contamination from signal models targeted by the edge search can be up to 80% relative to the expected background. In addition, the contamination from on-shell *Z* signals in the region used to validate the $Z/\gamma ^{*}+\text {jets}$ and flavour-symmetric estimates, VR-S, is up to 60% for models with $m(\tilde{g})<1$ $\text {TeV}$. The signal contamination from the slepton models in the DF regions used to estimate the flavour-symmetric backgrounds in the edge search, CR-FS-low/medium/high in Table 4, is less than 20% for models with $m(\tilde{g})>600$ $\text {GeV}$. It is only the contamination in these $e\mu$ CRs that is relevant in terms of the model-dependent interpretation of the results, and its impact is further discussed in Sect. 10. In general, for models giving substantial contamination in the CRs, the signal-to-background ratio in the SRs is found to be large enough for this contamination to have negligible impact on the sensitivity of the search.

## Background estimation

The dominant background processes in the SRs are “flavour-symmetric” (FS) backgrounds, where the ratio of *ee*, $\mu \mu$ and $e\mu$ dileptonic branching fractions is 1:1:2 because the two leptons originate from independent $W\rightarrow \ell \nu$ decays. This background is dominated by $t\bar{t}$ (50–70%) and also includes *WW*, *Wt*, and $Z\rightarrow \tau \tau$ processes. The FS background constitutes 60–90% of the expected background in the SRs, and is estimated using control samples of $e\mu$ events.

As all the SRs have a high-$E_{\text {T}}^{\text {miss}}$ requirement, $Z/\gamma ^{*}+\text {jets}$ events only enter the SRs when there is large $E_{\text {T}}^{\text {miss}}$ originating from instrumental effects or from neutrinos in jet fragments. This background is generally small, but it is difficult to model with MC simulation and can mimic signal, particularly for the on-shell *Z* search. This background is estimated using a control sample of $\gamma +\text {jets}$ events in data, which are kinematically similar to $Z/\gamma ^{*}+\text {jets}$ and have similar sources of $E_{\text {T}}^{\text {miss}}$.

The production of *WZ* / *ZZ* dibosons contributes approximately 30% of the SM background in SRZ and up to 20% of the background in the edge SR $m_{\ell \ell }$ windows. These backgrounds are estimated from MC simulation, after validation in dedicated $3\ell$ (*WZ*) and $4\ell$ (*ZZ*) VRs. Rare top backgrounds, which include $t\bar{t}W$, $t\bar{t}Z$ and $t\bar{t}WW$ processes, constitute <5% of the expected SM background in all SRs, and are estimated from MC simulation. The contribution from events with fake or misidentified leptons is at most 15% (in one of the edge $m_{\ell \ell }$ ranges in SR-low), but is generally <5% of the expected SM background in most SRs.

### Flavour-symmetric backgrounds

The flavour-symmetric background is dominant in all SRs. To estimate the contribution of this background to each SR, the so-called “flavour-symmetry” method, detailed in Ref. [20], is used. In this method, data events from a DF control sample, which is defined with the same kinematic requirements as the SR, are used to determine the expected event yields in the SF channels. In the on-shell *Z* analysis, the method is used to predict the background yield in the *Z* mass window, defined as $81<m_{\ell \ell }<101$ $\text {GeV}$. In the edge analysis, the method is extended to predict the full dilepton mass shape, such that a prediction can be extracted in any of the predefined $m_{\ell \ell }$ windows.

For the edge search, the flavour-symmetric contribution to each $m_{\ell \ell }$ bin of the signal regions is predicted using data from the corresponding bin in an $e\mu$ control region. All edge CR-FS regions (definitions can be seen in Table 4) are 88–97% pure in flavour-symmetric processes (this purity is calculated from MC simulation).

For the on-shell search, this method is complicated slightly by a widening of the $m_{\ell \ell }$ window used in CR-FS, the $e\mu$ control region (defined in Table 3). The window is enlarged to $61<m_{\ell \ell }<121$ $\text {GeV}$ to approximately triple the amount of data in the control region and thus increase the statistical precision of the method. This results in a region that is $\sim$95% pure in flavour-symmetric processes (the expected composition of this 95% is $\sim$80% $t\bar{t}$, $\sim$10% *Wt*, $\sim$10% *WW* and <1% $Z \rightarrow \tau \tau$).

Apart from the $m_{\ell \ell }$ widening in CR-FS, the method used is identical for the on-shell and edge regions. Events in the control regions are subject to lepton $p_{\text {T}}$- and $\eta$-dependent correction factors measured in data and MC simulation. Because the triggers used are not identical in 2015 and 2016, these factors are measured separately for each year and account for the different identification and reconstruction efficiencies for electrons and muons, as well as the different trigger efficiencies for the dielectron, dimuon and electron–muon selections. The estimated numbers of events in the SF channels, $N^\text {est}_{ee/\mu \mu }$, are given by:

1 $$\begin{aligned} N_{ee}^\text {est}= & {} \frac{1}{2} \cdot f_{\mathrm {FS}} \cdot f_{Z \mathrm {\text {-}mass}} \cdot \sum ^{N_{e\mu }^\text {data}}_{i} k_{e}(p_{\text {T}} ^{i,\mu }, \eta ^{i,\mu })\cdot \alpha (p_{\text {T}} ^{i,\mu }, \eta ^{i,\mu }) ,\end{aligned}$$

2 $$\begin{aligned} N_{\mu \mu }^\text {est}= & {} \frac{1}{2} \cdot f_{\mathrm {FS}} \cdot f_{Z \mathrm {\text {-}mass}} \cdot \sum ^{N_{e\mu }^\text {data}}_{i} k_{\mu }(p_{\text {T}} ^{i,e}, \eta ^{i,e})\cdot \alpha (p_{\text {T}} ^{i,e}, \eta ^{i,e}) , \end{aligned}$$

where $N_{e\mu }^\text {data}$ is the number of data events observed in a given control region, $\alpha (p_{\text {T}} ^i, \eta ^i)$ accounts for the different trigger efficiencies for SF and DF events, and $k_{e}(p_{\text {T}} ^{i,\mu }, \eta ^{i,\mu })$ and $k_{\mu }(p_{\text {T}} ^{i,e}, \eta ^{i,e})$ are electron and muon selection efficiency factors for the kinematics of the lepton being replaced, in event *i*. The trigger and selection efficiency correction factors are derived from the events in an inclusive on-*Z* selection ($81<m_{\ell \ell }<101$ $\text {GeV}$, $\ge 2$ jets), according to:

3 $$\begin{aligned} k_{e}(p_{\text {T}}, \eta )= & {} \sqrt{\frac{N_{ee}^{\text {meas}(p_{\text {T}}, \eta )}}{N_{\mu \mu }^{\text {meas}(p_{\text {T}}, \eta )}}} \end{aligned}$$

4 $$\begin{aligned} k_{\mu }(p_{\text {T}}, \eta )= & {} \sqrt{\frac{N_{\mu \mu }^{\text {meas}(p_{\text {T}}, \eta )}}{N_{ee}^{\text {meas}(p_{\text {T}}, \eta )}}} \end{aligned}$$

5 $$\begin{aligned} \alpha (p_{\text {T}}, \eta )= & {} \frac{\sqrt{\epsilon ^\text {trig}_{ee}(p_{\text {T}} ^{\ell _1},\eta ^{\ell _1}) \times \epsilon ^\text {trig}_{\mu \mu }(p_{\text {T}} ^{\ell _1},\eta ^{\ell _1})}}{\epsilon ^\text {trig}_{e\mu }(p_{\text {T}} ^{\ell _1},\eta ^{\ell _1})} \end{aligned}$$

where $$\epsilon ^\text {trig}_{ee/\mu \mu }$$ is the trigger efficiency and $$N_{ee/\mu \mu }^{\text {meas}}$$ is the number of $$ee/\mu \mu $$ events in the inclusive on-*Z* region outlined above. Here $$k_{e}(p_{\text {T}}, \eta )$$ and $$k_{\mu }(p_{\text {T}}, \eta )$$ are calculated separately for leading and sub-leading leptons, while $$\alpha $$ is calculated for the leading lepton, $$\ell _1$$. The correction factors are typically within 10% of unity, except in the region $$|\eta |<0.1$$ where, because of the lack of coverage by the muon spectrometer, they are up to 50% from unity. For all background estimates based on the flavour-symmetry method, results are computed separately for *ee* and $$\mu \mu $$ and then summed to obtain the combined predictions. The resulting estimates from the DF channels are scaled according to the fraction of flavour-symmetric backgrounds in each $$e\mu $$ control sample, $$f_{\mathrm {FS}}$$ ($$95\%$$ in CR-FS), which is determined by subtracting non-flavour-symmetric backgrounds taken from MC simulation from the data observed in the corresponding $$e\mu $$ region. In the on-shell case, the result is also scaled by the fraction of events in CR-FS expected to be contained within $$81<m_{\ell \ell }<101$$ $$\text {GeV}$$, $$f_{Z \text {-mass}}$$ ($$38\%$$), which is otherwise set to 100% for the edge regions. The validity of extrapolating in $$m_{\ell \ell }$$ between CR-FS and SRZ was checked by comparing the $$m_{\ell \ell }$$ shapes in data and MC simulation in a region similar to VR-S, but with the $$m_{\ell \ell }$$ requirement relaxed and $$H_{\mathrm {T}}^{\mathrm {incl}}$$ $$ > 300$$ $$\text {GeV}$$ to obtain a sample with a large number of events. The resulting on-*Z* fractions in MC simulation were found to agree with data within statistical uncertainties, which are summed in quadrature to assign a systematic uncertainty. In the case of the edge search the full $$m_{\ell \ell }$$ distribution is validated by applying a flavour-symmetry method to $$t\bar{t}$$ MC evnets in VR-low, VR-medium and VR-high. This procedure results in good closure, which is further discussed in Sect. 7.5. The difference between the prediction and the observed distribution is used to assign an MC non-closure uncertainty to the estimate.

The flavour-symmetry method in SRZ is further cross-checked by performing a profile likelihood fit [90] of MC yields to data in the *Z*-mass sidebands ($m_{\ell \ell }\notin [81, 101]$ $\text {GeV}$), the region denoted CRT in Table 3, which is dominated by $t\bar{t}$ (with a purity of >75%) and contains 273 events in data. The other flavour-symmetric processes in this region contribute $\sim$12% (*Wt*), 10% (*WW*) and <1% ($Z\rightarrow \tau \tau$). All SM background processes are taken directly from MC simulation in this cross-check, including backgrounds also estimated using the flavour-symmetry method. The normalisation of the dominant $t\bar{t}$ background is a free parameter and is the only parameter affected by the fit. For this cross-check, the contamination from Beyond Standard Model processes in the *Z*-mass sidebands is assumed to be negligible. The fit results in a scale factor of 0.64 for the $t\bar{t}$ yield predicted by simulation. This result is extrapolated from the *Z*-mass sidebands to SRZ and gives a prediction of $29 \pm 7$ events, which is consistent with the nominal flavour-symmetry background estimate of $33 \pm 4$ in this region.

The sideband fit is repeated at lower $E_{\text {T}}^{\text {miss}}$ in VRT, with the results being propagated to VR-S, so as to test the $m_{\ell \ell }$ extrapolation used in the sideband fit method. The normalisation to data in this region, which is at lower $E_{\text {T}}^{\text {miss}}$ relative to CRT, results in a scale factor of 0.80 for the $t\bar{t}$ yield predicted by simulation. The number of FS events predicted in VR-S using the sideband fit in VRT is compatible with the number estimated by applying the FS method to data in VR-FS. The results of the background estimate in both VR-S and SRZ obtained from the flavour-symmetry method are compared with the values obtained by the sideband fit cross-check in Table 5. The methods result in consistent estimates in both regions. Further results in the edge VRs are discussed in Sect. 7.5.

**Table 5:** Comparison of the predicted yields for the flavour-symmetric backgrounds in SRZ and VR-S as obtained from the nominal data-driven method using CR-FS and the *Z*-mass sideband method. The quoted uncertainties include statistical and systematic contributions

Region	Flavour-symmetry	Sideband fit
SRZ	$33 \pm 4$	$29 \pm 7$
VR-S	$99\pm 8$	$92 \pm {25}$

A potential cause of the low scale factors obtained from the sideband fit at large $H_{\text {T}}$ and $E_{\text {T}}^{\text {miss}}$ is mismodelling of the top-quark $p_{\text {T}}$ distribution, where measurements of $t\bar{t}$ differential cross sections by the ATLAS and CMS experiments indicate that the top-quark $p_{\text {T}}$ distribution predicted by most generators is harder than that observed in data [91, 92]. Corrections to the MC predictions according to NNLO calculations provided in Ref. [93] indicate an improvement in the top-quark pair modelling at high $H_{\text {T}}$, which should lead to scale factors closer to unity. With the data-driven method used to estimate $t\bar{t}$ contributions in this analysis, the results do not depend on these corrections. They are therefore not applied to the $t\bar{t}$ MC sample for the sideband-fit cross-check.

### $Z/\gamma ^{*}+\text {jets}$ background

The $Z/\gamma ^{*}+\text {jets}$ background estimate is based on a data-driven method that uses $\gamma +\text {jets}$ events in data to model the $E_{\text {T}}^{\text {miss}}$ distribution of $Z/\gamma ^{*}+\text {jets}$. The $\gamma +\text {jets}$ and $Z/\gamma ^{*}+\text {jets}$ processes have similar event topologies, with a well-measured object recoiling against a hadronic system, and both tend to have $E_{\text {T}}^{\text {miss}}$ that stems from jet mismeasurements and neutrinos in hadronic decays. In this method, which has been used by CMS in a search in this final state [18], a sample of data events containing at least one photon and no leptons is constructed using the same kinematic selection as each of the SRs, without the $E_{\text {T}}^{\text {miss}}$ and $\Delta \phi (\text {jet}_{12},{\varvec{p}}_{\mathrm {T}}^\mathrm {miss})$ requirements (the CR$\gamma$ regions defined in Tables 3, 4).

The requirement $\Delta \phi (\text {jet}_{12},{\varvec{p}}_{\mathrm {T}}^\mathrm {miss}) > 0.4$ applied in the SRs suppresses $E_{\text {T}}^{\text {miss}}$ from jet mismeasurements and increases the relative contributions to $E_{\text {T}}^{\text {miss}}$ from the photon, electrons, and muons. The difference in resolution between photons, electrons, and muons can be significant at high $p_{\text {T}}$. Therefore, before the $\Delta \phi (\text {jet}_{12},{\varvec{p}}_{\mathrm {T}}^\mathrm {miss}) > 0.4$ requirement is applied, the photon $p_{\text {T}}$ is smeared according to a $Z\rightarrow ee$ or $Z\rightarrow \mu \mu$ resolution function. The smearing function is derived by comparing the $E_{\text {T}}^{\text {miss}}$-projection along the boson momentum in $Z/\gamma ^{*}+\text {jets}$ and $\gamma +\text {jets}$ MC events in a 1-jet control region with no other event-level kinematic requirements. A deconvolution is applied to avoid including the photon resolution in the *Z* resolution. For each event, a photon $p_{\text {T}}$ smearing $\Delta p_{\text {T}}$ is obtained by sampling the smearing function. The photon $p_{\text {T}}$ is shifted by $\Delta p_{\text {T}}$, with the parallel component of the $E_{\text {T}}^{\text {miss}}$ being correspondingly adjusted by $-\Delta p_{\text {T}}$.

The smeared $\gamma +\text {jets}$ events are then reweighted to match the boson $p_{\text {T}}$ distribution of the $Z/\gamma ^{*}+\text {jets}$ events. This reweighting is applied separately in each region and accounts for small differences between the $\gamma +\text {jets}$ events and $Z/\gamma ^{*}+\text {jets}$ events, which arise mainly from the mass of the *Z* boson. The reweighting is done using $Z/\gamma ^{*}+\text {jets}$ events in data, and is checked using $Z/\gamma ^{*}+\text {jets}$ MC simulation in an MC closure test, as described further below. Following this smearing and reweighting procedure, the $E_{\text {T}}^{\text {miss}}$ of each $\gamma +\text {jets}$ event is recalculated, and the final $E_{\text {T}}^{\text {miss}}$ distribution is obtained after applying the $\Delta \phi (\text {jet}_{12},{\varvec{p}}_{\mathrm {T}}^\mathrm {miss} ) > 0.4$ requirement. For each SR, the resulting $E_{\text {T}}^{\text {miss}}$ distribution is normalised to data in a CRZ with the same requirements except that the SR $E_{\text {T}}^{\text {miss}}$ requirement is replaced by $E_{\text {T}}^{\text {miss}} <60$ $\text {GeV}$.

The shape of the $Z/\gamma ^{*}+\text {jets}$ $m_{\ell \ell }$ distribution is extracted from MC simulation and validated by comparing to data in events with lower $E_{\text {T}}^{\text {miss}}$ requirements and a veto on *b*-tagged jets, to suppress the background from $t\bar{t}$. The $m_{\ell \ell }$ distribution is modelled by parameterising the $m_{\ell \ell }$ in $Z/\gamma ^{*}+\text {jets}$ events as a function of the difference between reconstructed and true *Z* boson $p_{\text {T}}$ in MC simulation. This parameterization ensures that the correlation between lepton momentum mismeasurement and observed $m_{\ell \ell }$ values far from the *Z* boson mass is preserved. Each photon event is assigned an $m_{\ell \ell }$ via a random sampling of the corresponding distribution, equating photon $\Delta p_{\text {T}}$ and the difference between true and reconstructed *Z* boson $p_{\text {T}}$. The resulting $m_{\ell \ell }$ distribution in $\gamma +\text {jets}$ MC simulation is compared to that extracted from $Z/\gamma ^{*}+\text {jets}$ MC simulation and the difference is assessed as a systematic uncertainty in the background prediction for each $m_{\ell \ell }$ bin.

The full smearing, reweighting, and $m_{\ell \ell }$ assignment procedure is applied to the $V\gamma$ MC sample in parallel with the $\gamma +\text {jets}$ data sample. After applying all corrections to both samples, the $V\gamma$ contribution to the $\gamma +\text {jets}$ data sample is subtracted to remove contamination from backgrounds with real $E_{\text {T}}^{\text {miss}}$. Contamination by events with fake photons in these $\gamma +\text {jets}$ data samples is small, and this contribution is therefore neglected.

In the $H_{\text {T}}$-inclusive region corresponding to VR-low, there is a non-negligible contribution expected from $Z/\gamma ^{*}+\text {jets}$ events with $p_{\text {T}} ^{Z}<37$ $\text {GeV}$. Given the photon trigger strategy discussed in Sect. 4, no photons with $p_{\text {T}} <37$ $\text {GeV}$ are included in the event selection. To account for this photon $p_{\text {T}}$ threshold, a boson-$p_{\text {T}}$ correction of up to 50% is applied as a function of $E_{\text {T}}^{\text {miss}}$ in VR-low. This correction uses the fraction of $Z/\gamma ^{*}+\text {jets}$ events in a given $E_{\text {T}}^{\text {miss}}$ bin expected to have $p_{\text {T}} ^{Z}<37$ $\text {GeV}$, according to MC simulation. The $\gamma +\text {jets}$ data are then scaled according to this fraction, as a function of $E_{\text {T}}^{\text {miss}}$, to correct for the missing $p_{\text {T}} ^{Z}<37$ $\text {GeV}$ contribution. The correction is found to be negligible in all signal regions.

**Fig. 3:**
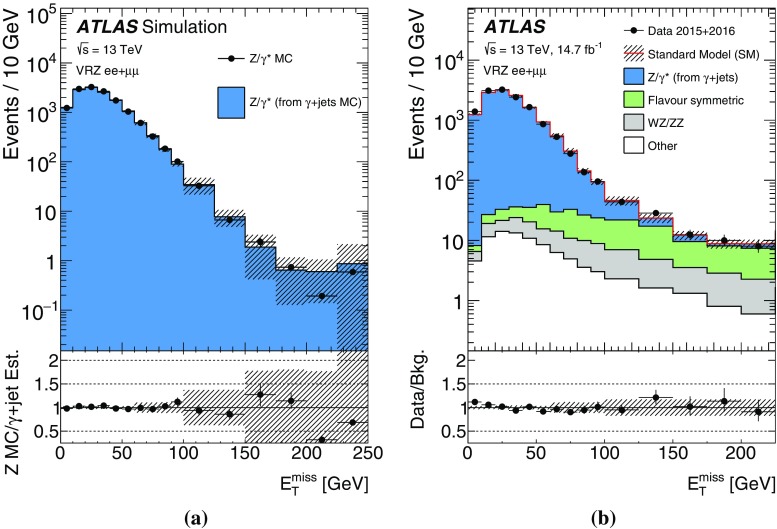
*Left* the $E_{\text {T}}^{\text {miss}}$ spectrum in Sherpa $Z/\gamma ^{*}+\text {jets}$ MC simulation compared to that of the $\gamma +\text {jets}$ background estimation technique applied to Sherpa $\gamma +\text {jets}$ MC simulation in VRZ. The *error bars* on the points indicate the statistical uncertainty of the $Z/\gamma ^{*}+\text {jets}$ MC simulation, and the *hashed uncertainty bands* indicate the statistical and reweighting systematic uncertainties of the $\gamma +$jet background method. For this MC comparison the upper $E_{\text {T}}^{\text {miss}}$ cut has been removed from VRZ and the overflow is included in the rightmost bin. *Right* the $E_{\text {T}}^{\text {miss}}$ spectrum when the method is applied to data in VRZ. Here the flavour-symmetric background is estimated using the data-driven flavour-symmetry method, and the fake-lepton background is estimated using the data-driven method explained in Sect. 7.3. Rare top and diboson backgrounds are taken from MC simulation. The rare top and data-driven fake-lepton backgrounds are grouped under “other” backgrounds. The *hashed bands* indicate the systematic uncertainty of only the $\gamma +\text {jets}$ and flavour-symmetric backgrounds below 100 $\text {GeV}$ and the full uncertainty of the VR-S prediction above 100 $\text {GeV}$. The *bottom panel* of each figure shows the ratio of the observation (*left* in MC simulation; *right* in data) to the prediction

The distribution of $E_{\text {T}}^{\text {miss}}$ obtained in Sherpa $Z/\gamma ^{*}+\text {jets}$ MC simulation is compared to that obtained by applying this background estimation technique to Sherpa $\gamma +\text {jets}$ MC samples. In this check the $\gamma +\text {jets}$ MC simulation is reweighted according to the $p_{\text {T}}$ distribution given by the $Z/\gamma ^{*}+\text {jets}$ MC simulation. The result of this MC closure check is shown in Fig. 3a for events in VRZ (without an upper $E_{\text {T}}^{\text {miss}}$ cut), where good agreement between $Z/\gamma ^{*}+\text {jets}$ and corrected $\gamma +\text {jets}$ MC simulation can be seen across the entire $E_{\text {T}}^{\text {miss}}$ spectrum. A comparison between the full $E_{\text {T}}^{\text {miss}}$ spectrum in data and the $Z/\gamma ^{*}+\text {jets}$ background estimated via the $\gamma +\text {jets}$ method is also shown in Fig. 3b for events in VRZ. The systematic uncertainties associated with this method are described in Sect. 8.

### Fake-lepton background

Semileptonic $t\bar{t}$, $W\rightarrow \ell \nu$ and single top (*s*- and *t*-channel) events enter the dilepton channels via “fake” leptons. These can include misidentified hadrons, converted photons or non-prompt leptons from *b*-hadron decays. The extent of this background is estimated using the matrix method, detailed in Ref. [94]. Its contribution in regions with high lepton $p_{\text {T}}$ and dilepton invariant mass is negligible, but in the edge search, where lower-$p_{\text {T}}$ leptons are selected and events can have low $m_{\ell \ell }$, the fake-lepton background can make up to 15% of the total background. In this method a control sample is constructed using baseline leptons, thereby enhancing the probability of selecting a fake lepton due to the looser lepton selection and identification criteria relative to the signal lepton selection. For each relevant CR, VR or SR, the region-specific kinematic requirements are placed upon this sample of baseline leptons. The number of events in this sample in which the selected leptons subsequently pass ($N_{\text {pass}}$) or fail ($N_{\text {fail}}$) the signal lepton requirements in Sect. 5 are then counted. In the case of a one-lepton selection, the number of fake-lepton events in a given region is then estimated according to:

6 $$\begin{aligned} N_{\text {pass}}^{\text {fake}} = \frac{N_{\text {fail}} - (1/\epsilon ^{\text {real}} - 1) \times N_{\text {pass}} }{1/\epsilon ^{\text {fake}} - 1/\epsilon ^{\text {real}}}. \end{aligned}$$

Here $\epsilon ^{\text {real}}$ is the relative identification efficiency (from baseline to signal) for genuine, prompt (“real”) leptons and $\epsilon ^{\text {fake}}$ is the relative identification efficiency (again from baseline to signal) with which non-prompt leptons or jets might be misidentified as prompt leptons. This principle is then expanded to a dilepton selection by using a four-by-four matrix to account for the various possible real–fake combinations for the two leading leptons in an event.

The real-lepton efficiency, $\epsilon ^{\text {real}}$, is measured in $Z\rightarrow \ell \ell$ data events using a tag-and-probe method in CR-real, defined in Table 4. In this region the $p_{\text {T}}$ of the leading lepton is required to be $>$40 $\text {GeV}$, and only events with exactly two SFOS leptons are selected. The fake-lepton efficiency, $\epsilon ^{\text {fake}}$, is measured in CR-fake, a region enriched with fake leptons by requiring same-sign lepton pairs. The lepton $p_{\text {T}}$ requirements are the same as those in CR-real, with the leading lepton being tagged as the “real” lepton and the fake efficiency being evaluated based on the sub-leading lepton in the event. An $E_{\text {T}}^{\text {miss}}$ requirement of <125 $\text {GeV}$ is used to reduce possible contamination from Beyond Standard Model processes. In this region the background due to prompt-lepton production, estimated from MC simulation, is subtracted from the total data contribution. Prompt-lepton production makes up 7% (11%) of the baseline electron (muon) sample and 10% (61%) of the signal electron (muon) sample in CR-fake. From the resulting data sample the fraction of events in which the baseline leptons pass a signal-like selection yields the fake efficiency. Both the real- and fake-lepton efficiencies are binned as a function of lepton $p_{\text {T}}$ and calculated separately for the 2015 and 2016 data sets.

This method is validated by checking the closure in MC simulation and data–background agreement in VR-fake.

### Diboson and rare top processes

The remaining SM background contribution in the SRs is due to *WZ* / *ZZ* diboson production and rare top processes ($t\bar{t} Z$, $t\bar{t} W$ and $t\bar{t} WW$). The rare top processes compose <5% of the expected SM background in the SRs and are taken directly from MC simulation.

Production of *WZ* / *ZZ* dibosons constitutes about 30% of the expected background in SRZ and up to 20% in some edge SR $m_{\ell \ell }$ windows. In SRZ, this background is composed of roughly 70% *WZ*, about 40% of which is $WZ\rightarrow \ell \ell \tau \nu$. This is the largest background contribution that is estimated from MC simulation, and must be carefully validated, especially because these backgrounds contain *Z* bosons and can thus mimic a signal by producing a peak at $m_{\ell \ell }\approx m_Z$. To validate the MC modelling of these backgrounds, VRs with three leptons (VR-WZ) and four leptons (VR-ZZ) are defined (selection shown in Table 3). In VR-WZ, from the three selected leptons in an event, the SFOS pair with $m_{\ell \ell }$ most consistent with the *Z* mass is indentified as the *Z* candidate. The transverse mass of the remaining lepton and the $E_{\text {T}}^{\text {miss}}$, $m_{\text {T}}(\ell _{3},E_{\text {T}}^{\text {miss}})$, is then required to be <100 $\text {GeV}$, forming the *W* candidate. In VR-ZZ an $E_{\text {T}}^{\text {miss}} <100$ $\text {GeV}$ requirement is used to suppress *WZ* and top processes. The yields and kinematic distributions observed in these regions are well-modelled by MC simulation. In particular, the $E_{\text {T}}^{\text {miss}}$, $H_\text {T}$, jet multiplicity, and boson $p_{\text {T}}$ distributions show good agreement. An additional three-lepton VR (VR-3L) is defined to provide validation of the diboson background in a region of phase space closer to the SR; good agreement is observed in this region as well.

### Results in validation regions

The expected background yields in VR-S are shown in Table 6 and compared with the observed data yield. Agreement between the data and the expected Standard Model background is observed. The expected background yields in the three diboson VRs are also shown in Table 6. The data are consistent with the expected background. Similar information for the edge VRs is provided in Table 7. Data and background estimates are in agreement within uncertainties.

**Fig. 4:**
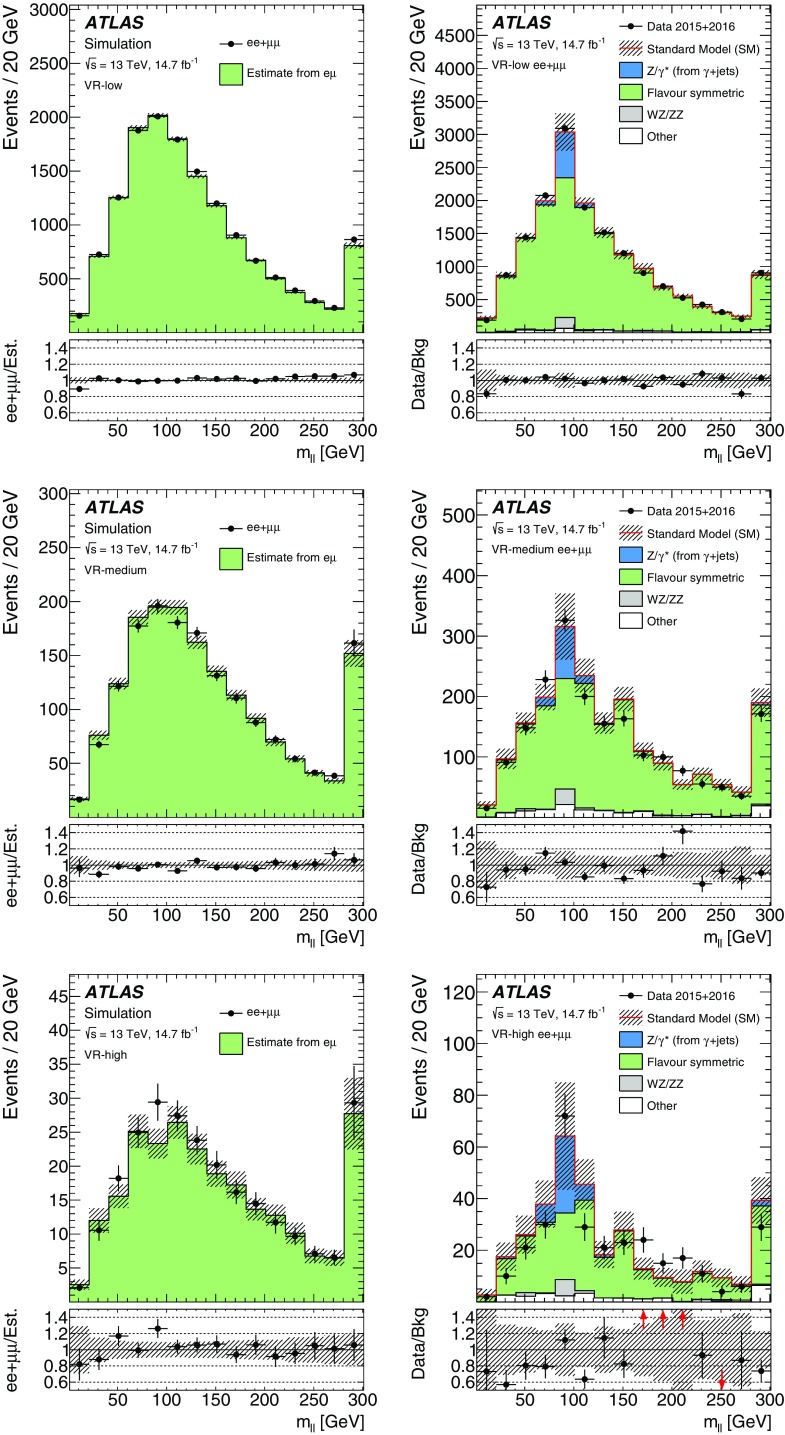
Validation of the flavour-symmetry method for the edge search using MC events (*left*) and data (*right*), in the VR-low (*top*), VR-medium (*middle*), and VR-high (*bottom*) regions. In the MC plots the flavour-symmetry estimate from $e\mu$ $t\bar{t}$ MC samples is compared with the observed SF distribution from these MC samples, with the MC statistical uncertainty indicated by the *hashed bands*. In the data plots, all uncertainties in the background prediction are included in the *hashed band*. The rare top and data-driven fake-lepton backgrounds are grouped under “other” backgrounds. The *bottom panel* of each figure shows the ratio of the observation (*left* in MC simulation; *right* in data) to the prediction. In cases where the data point is not accommodated by the scale of this *panel*, a *red arrow* indicates the direction in which the point is out of range. The last bin contains the overflow

Figure 4 shows the observed and expected $m_{\ell \ell }$ distributions in the same edge VRs. The same background estimation methods are applied to both MC simulation and data. In the MC studies, the flavour-symmetry method of Sect. 7.1 is applied to $t\bar{t}$ MC simulation, and the observed SF $m_{\ell \ell }$ distribution is compared to the prediction based on DF events. In the data studies, the observed SF $m_{\ell \ell }$ distribution is compared to the sum of FS backgrounds from the extended flavour-symmetry method, the $Z/\gamma ^{*}+\text {jets}$ background from the $\gamma +\text {jets}$ method, and the *WZ* / *ZZ* diboson, rare top, and fake-lepton backgrounds.

The observed MC closure is good in all validation regions. The data agree with the expected background in the validation regions as well. No significant discrepancies or trends are apparent.

**Table 6:** Expected and observed event yields in the four validation regions, VR-S, VR-WZ, VR-ZZ, and VR-3L. The flavour-symmetric, $Z/\gamma ^{*}+\text {jets}$, and fake-lepton contributions to VR-S are derived using the data-driven estimates described in Sect. 7. All remaining backgrounds, and all backgrounds in the diboson validation regions, are taken from MC simulation. The quoted uncertainties in VR-S include statistical and all systematic contributions. In VR-WZ, VR-ZZ, and VR-3L, the rare top and diboson uncertainties include statistical and all theoretical uncertainties described in Sect. 8. The fake-lepton contribution in these three regions is predominantly due to $Z/\gamma ^{*}+\text {jets}$, and in this case only the statistical uncertainty is given. The individual uncertainties can be correlated and do not necessarily add up in quadrature to the total systematic uncertainty

	VR-S	VR-WZ	VR-ZZ	VR-3L
Observed events	236	698	132	32
Total expected background events	$224 \pm 41$	$622\pm 66$	$139\pm 25$	$35\pm 10$
Flavour-symmetric ($t\bar{t}$, *Wt*, *WW*, $Z\rightarrow \tau \tau$)	$99\pm 8$	–	–	–
*WZ* / *ZZ* events	$27 \pm 13$	$573\pm 66$	$139\pm 25$	$25\pm 10$
Rare top events	$11\pm 3$	$14\pm 3$	$0.44 \pm 0.11$	$9.1\pm 2.3$
$Z/\gamma ^{*}+\text {jets}$ events	$84 \pm 37$	–	–	–
Fake-lepton events	${4} \pm {4}$	$35\pm 6$	–	$0.6\pm 0.3$

**Table 7:** Expected and observed event yields in the three validation regions, VR-low, VR-medium and VR-high. The quoted uncertainties include statistical and systematic contributions. The individual uncertainties can be correlated and do not necessarily add up in quadrature to the total systematic uncertainty

	VR-low	VR-medium	VR-high
Observed events	16,253	1917	314
Total expected background events	16,500 $\pm 700$	$1990\pm 150$	$340\pm 60$
Data-driven flavour-symmetry events	14,700 $\pm 600$	$1690\pm 120$	$250\pm 50$
*WZ* / *ZZ* events	$250 \pm 80$	${40} \pm {19}$	${9}\pm {6}$
Data-driven $Z/\gamma ^{*}+\text {jets}$ ($\gamma +\text {jets}$) events	${ 1100}\pm {400}$	$130\pm 70$	${ 50}\pm {29}$
Rare top events	${ 87} \pm {23}$	$27\pm 7$	${ 6.5}\pm {1.8}$
Data-driven fake-lepton events	${ 270}\pm {100}$	${ 98}\pm {35}$	${ 20}\pm {11}$

## Systematic uncertainties

The data-driven background estimates are subject to uncertainties associated with the methods employed and the limited number of events used in their estimation. The dominant uncertainty (10%) for the flavour-symmetry-based background estimate in SRZ is due to the limited number of events in CR-FS. Other systematic uncertainties assigned to this background estimate include those due to MC closure (3%), the measurement of the efficiency correction factors (3%) and the extrapolation in $m_{\ell \ell }$ (1%). In the case of the edge SRs the statistical uncertainty is also the dominant uncertainty in the flavour-symmetric background estimate in the case of SR-high, but for both SR-medium and SR-low the uncertainties from the MC non-closure and efficiency correction factors are comparable in size, or in some cases larger. These uncertainties can contribute up to 5% in SR-low and SR-medium and 10% in SR-high.

Several sources of systematic uncertainty are assessed for the $Z/\gamma ^{*}+\text {jets}$ background. The boson $p_{\text {T}}$ reweighting procedure is assigned an uncertainty based on a comparison of the nominal results with those obtained by reweighting using three other kinematic variables, namely $H_{\text {T}}$, *Z*-boson $E_{\text {T}}$ and jet multiplicity. For the smearing function, which is measured using MC events in a 1-jet control region, an uncertainty is derived by comparing the results obtained using the nominal smearing function with those obtained using a smearing function from a 2-jet sample of MC events, and also using a smearing function measured in a 1-jet data sample. An uncertainty of between 40–100% is assigned to account for different reweighting procedures and between 20–100% for the smearing procedure applied to $\gamma +\text {jets}$ events. The smearing uncertainty dominates in SR-high, while the reweighting uncertainty dominates in SR-low and SR-medium, with both being around 60% in SRZ. The full reweighting and smearing procedure is carried out using $\gamma +\text {jets}$ MC events such that an MC non-closure uncertainty can be derived by comparing the resulting $\gamma +\text {jets}$ MC $E_{\text {T}}^{\text {miss}}$ distribution to that in $Z/\gamma ^{*}+\text {jets}$ MC events. The resulting uncertainty of up to 35% is calculated in the VRs, so as to maximise the number of events that contribute. An uncertainty of 16% is assessed for the $V\gamma$ backgrounds, based on data-to-MC agreement in a $V\gamma$-enriched control region. This uncertainty is propagated to the final $Z/\gamma ^{*}+\text {jets}$ estimate following the subtraction of the $V\gamma$ background. In VR-low, a correction is applied to the $E_{\text {T}}^{\text {miss}}$ distribution in $\gamma +\text {jets}$ events to account for the fraction of $Z/\gamma ^{*}+\text {jets}$ events in this $H_{\text {T}}$-inclusive region expected to have boson $p_{\text {T}}$ less than 37 $\text {GeV}$. The full size of this correction (up to 50% for $E_{\text {T}}^{\text {miss}} =150$ $\text {GeV}$) is applied as a systematic uncertainty. The $m_{\ell \ell }$ distribution assigned to $\gamma +\text {jets}$ MC events is compared to that of $Z/\gamma ^{*}+\text {jets}$ MC events, and the relative difference in a given $m_{\ell \ell }$ bin is assigned as an uncertainty. Finally, the statistical precision of the estimate also enters as a systematic uncertainty of $\sim$10% in the final background estimate. After applying the correction procedure, differences in the number of *b*-tagged jets between $Z/\gamma ^{*}+\text {jets}$ and $\gamma +\text {jets}$ are found to be negligible, indicating good agreement in heavy-flavour content.

The uncertainties in the fake-lepton background stem from the number of events in the regions used to measure the real- and fake-lepton efficiencies, the limited size of the inclusive loose-lepton sample, and from varying the region used to measure the fake-lepton efficiency. The nominal fake-lepton efficiency is compared with those measured in a region with *b*-tagged jets and a region with a *b*-jet, as well as a region with the prompt-lepton subtraction varied by $20\%$. Varying the sample composition via *b*-jet tagging gives the largest uncertainty. The uncertainty for the edge SRs from the statistical component of the lepton efficiencies is 30–45%, and from varying the region for the fake-lepton efficiency it is 50–75%. The uncertainties in SRZ are generally larger due to the small number of events contributing to the estimate in this region.

Theoretical and experimental uncertainties are taken into account for the signal models, as well as background processes that rely on MC simulation. The estimated uncertainty in the luminosity measurement is $2.9\%$ [31, 32]. The jet energy scale is subject to uncertainties associated with the jet flavour composition, the pile-up and the jet and event kinematics [81]. Uncertainties in the jet energy resolution are included to account for differences between data and MC simulation [81]. An uncertainty in the $E_{\text {T}}^{\text {miss}}$ soft-term resolution and scale is taken into account [88], and uncertainties due to the lepton energy scales and resolutions, as well as trigger, reconstruction, and identification efficiencies, are also considered.

The *WZ* / *ZZ* processes are assigned a cross-section uncertainty of $6\%$ and an additional uncertainty based on comparisons between Sherpa and Powheg MC samples, which is up to $50\%$ in the SRs. Uncertainties due to the choice of factorisation and renormalisation scales are calculated by varying the nominal values up and down by a factor of two and can be up to $23\%$. For rare top processes, a $13\%$ PDF and scale variation uncertainty is applied [34] in addition to a $22\%$ cross-section uncertainty [61–63].

For signal models, the nominal cross section and the uncertainty are taken from an envelope of cross-section predictions using different PDF sets and factorisation and renormalisation scales, as described in Refs. [95, 96]. These are calculated at next-to-leading-logarithm accuracy (NLO + NLL) [51–55], and the resulting uncertainties range from 16 to 30%.

A breakdown of the dominant uncertainties in the background prediction in the SRs is provided in Table 8 for the on-shell *Z* and edge searches. Here these uncertainties are quoted relative to the total background. In the case of the edge regions a range is quoted, taking into account the relative contribution of the given uncertainty in each of the $m_{\ell \ell }$ ranges in SR-low, SR-medium and SR-high. The largest uncertainties in the signal regions are due to the size of the $e\mu$ data sample in CR-FS, used to provide the flavour-symmetric background estimate, the combined systematic uncertainty in the same background, the systematic uncertainty in $\gamma +\text {jets}$, or, in the case of SRZ, the *WZ* / *ZZ* generator uncertainty. The statistical component of the uncertainty from the flavour-symmetry estimate is largest for the edge analysis in SR-medium and SR-high in the highest $m_{\ell \ell }$ regions. In the edge SRs the uncertainty in the *WZ* / *ZZ* background tends to be highest in the $m_{\ell \ell }$ ranges that include the *Z* window. The uncertainty in the fake-lepton background is largest in SR-high, where fake leptons can compose a larger fraction of the background. Experimental uncertainties have a far lower impact on the systematic uncertainty of the total background (<2%).

**Table 8:** Overview of the dominant sources of systematic uncertainty in the total background estimate in the signal regions. The values shown are relative to the total background estimate, shown in %. The systematic uncertainties for the edge search are quoted as a range across the $m_{\ell \ell }$ regions used for statistical interpretations

Source	Relative systematic uncertainty [%]			
	SRZ	SR-low	SR-medium	SR-high
Total systematic uncertainty	17	8–30	6–34	10–45
*WZ* / *ZZ* generator uncertainty	13	0–7	0–6	0–10
Flavour symmetry (statistical)	7	3–16	5–16	7–28
*WZ* / *ZZ* scale uncertainty	6	0–1	0–1	0–2
$Z/\gamma ^{*}+\text {jets}$ (systematic)	4	0–15	0–25	0–15
Flavour symmetry (systematic)	3	2–23	2–15	4–25
$Z/\gamma ^{*}+\text {jets}$ (statistical)	2	0–3	0–5	0–1
Fake leptons	1	0–17	2–18	2–20

## Results

### Results in SRZ

**Fig. 5:**
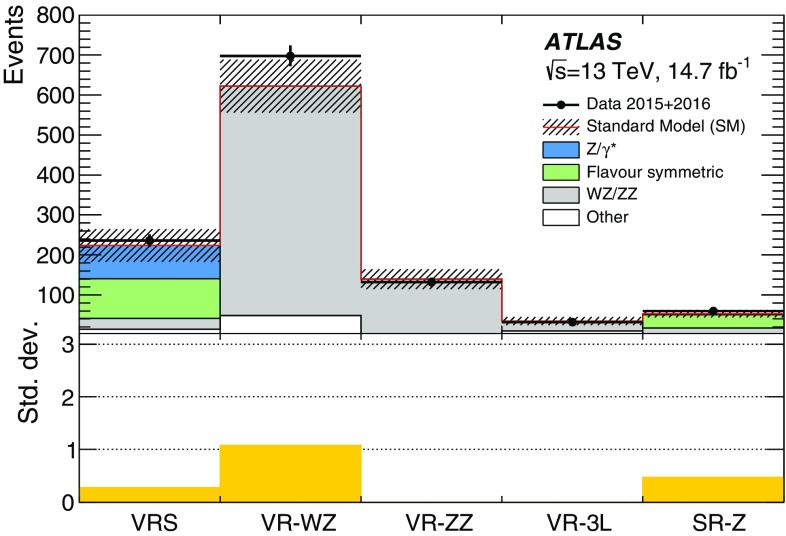
The expected and observed yields in the validation regions and signal region of the on-shell *Z* search. The rare top and data-driven fake-lepton backgrounds are grouped under “other” backgrounds. The significance of the difference between the data and the expected background (see *text* for details) is shown in the *bottom plot*; for regions in which the data yield is less than expected, the significance is set to zero. The *hashed uncertainty bands* include the statistical and systematic uncertainties in the background prediction

For the on-shell *Z* search, the expected background and observed yields in the SR are shown in Table 9. A total of 60 events are observed in data with a predicted background of $53.5 \pm 9.3$ events. There are 35 events observed in data in the *ee* channel, and 25 events observed in the $\mu \mu$ channel. The probability for the background to produce a fluctuation greater than or equal to that observed in the data, called the significance when expressed in terms of the number of standard deviations, corresponds to $0.47\sigma$ (details of the significance calculation are presented in Sect. 10). The level of agreement between the observed event yields in data and the background predictions in the VRs, shown previously in Table 6, is also displayed in Fig. 5, along with the results in SRZ.

**Table 9:** Expected and observed event yields in SRZ, inclusively, in the *ee* channel, and in the $\mu \mu$ channel, along with the discovery *p* value for zero signal strength ($p(s=0)$) [97], Gaussian significance, 95% confidence level (CL) observed and expected upper limits on the number of signal events ($S^{95}$), and the corresponding observed upper limit on the visible cross section ($\langle \epsilon \sigma \rangle ^{95}_\text {obs}$). For regions in which the data yield is less than expected, the discovery *p* value is truncated at 0.5 and the significance is set to zero. The flavour-symmetric, $Z/\gamma ^{*}+\text {jets}$ and fake-lepton components are all derived using data-driven estimates described in Sect. 7. All remaining backgrounds are taken from MC simulation. The quoted uncertainties include statistical and systematic contributions. The individual uncertainties can be correlated and do not necessarily add up in quadrature to the total systematic uncertainty

	SRZ	SRZ *ee*	SRZ $\mu \mu$
Observed events	60	35	25
Total expected background events	$53.5 \pm 9.3$	$27.1 \pm 5.1$	$26.8 \pm 4.4$
Flavour-symmetric ($t\bar{t}$, *Wt*, *WW* and $Z\rightarrow \tau \tau$) events	$33.2 \pm 3.9$	$16.5 \pm 2.1$	$16.7 \pm 2.0$
$Z/\gamma ^{*}+\text {jets}$ events	${3.1} \pm {2.8}$	$1.0_{-1.0}^{+1.3}$	${2.1} \pm {1.4}$
*WZ* / *ZZ* events	$14.2 \pm 7.7$	${7.8} \pm {4.3}$	${6.4} \pm {3.5}$
Rare top events	${2.9} \pm {0.8}$	${1.4} \pm {0.4}$	${1.5} \pm {0.4}$
Fake-lepton events	$$ {0.1}_{-0.1}^{+0.8}$$	$$0.5_{-0.5}^{+0.7}$$	$$ {0}^{+0.2}$$
$p(s=0)$	0.32	0.15	0.5
Significance $(\sigma )$	0.47	1.02	0
Observed (expected) $S^{95}$	28.2 ($24.5_{-6.7}^{+8.9}$)	22.0 ($15.8_{-4.5}^{+6.5}$)	12.9 ($14.0_{-3.9}^{+5.7}$)
$\langle \epsilon \sigma \rangle ^{95}_\text {obs}$ [fb]	1.9	1.5	0.88

**Fig. 6:**
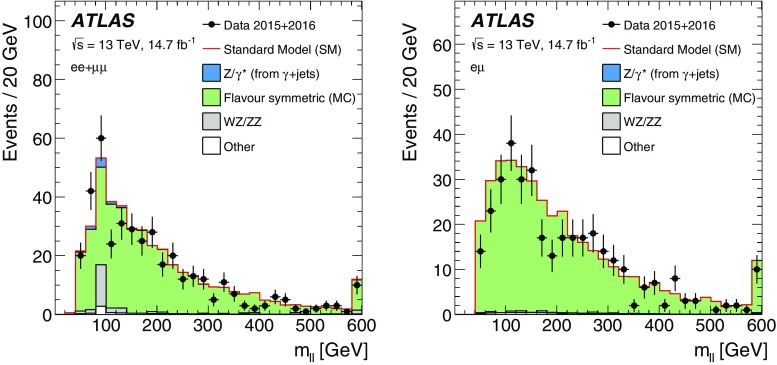
The dilepton invariant-mass distribution for an SRZ-like selection, but with the *Z* mass requirement removed, in the same-flavour (*left*) and different-flavour (*right*) channels. With the exception of the $Z/\gamma ^{*}+\text {jets}$ background, MC simulation is used to show the expected shapes of the $m_{\ell \ell }$ distributions, with the backgrounds being normalised according to their SRZ prediction. For the $Z/\gamma ^{*}+\text {jets}$ background, the $m_{\ell \ell }$ shape is taken from the $\gamma +\text {jets}$ method. The rare top and data-driven fake-lepton backgrounds are grouped under “other” backgrounds. The last bin includes the overflow

**Fig. 7:**
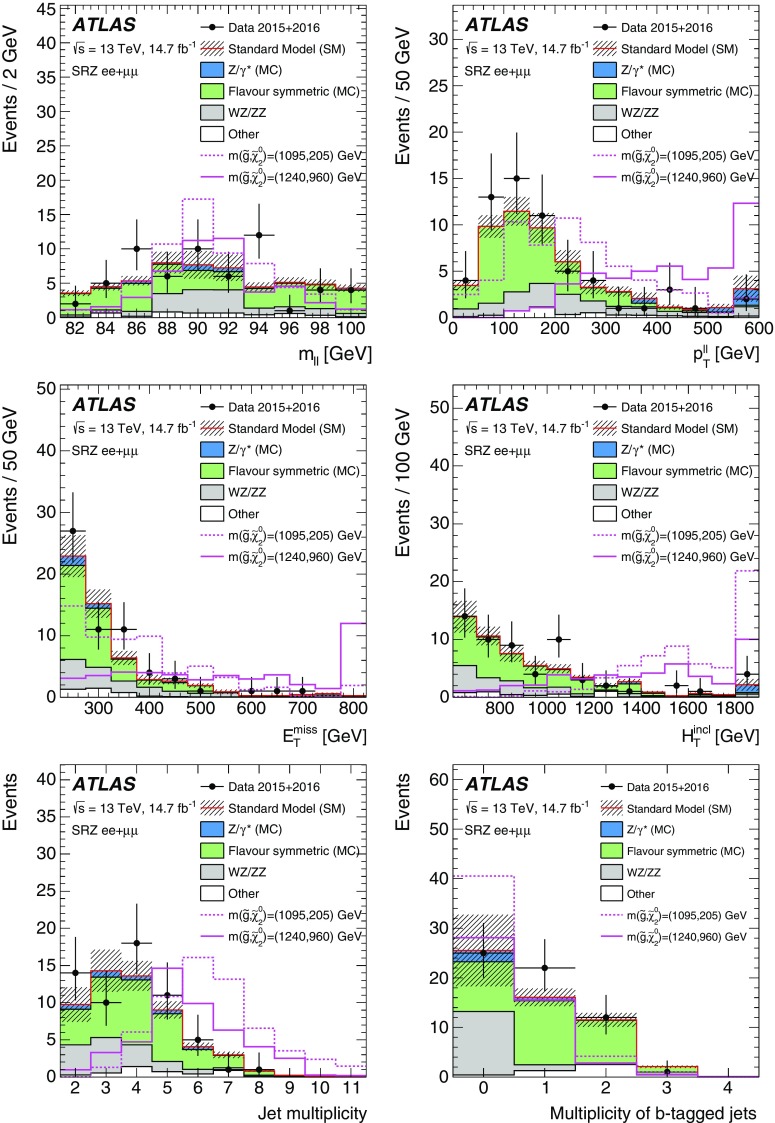
The $m_{\ell \ell }$ (*top left*), $p_{\text {T}} ^{\ell \ell }$ (*top right*), $E_{\text {T}}^{\text {miss}}$ (*middle left*), $H_{\mathrm {T}}^{\mathrm {incl}}$ (*middle right*), jet multiplicity (*bottom left*) and *b*-tagged jet multiplicity (*bottom right*) distributions in SRZ. Two examples of signal models from the $\tilde{g}$–$\tilde{\chi }_2^0$ on-shell grid, described in Sect. 4, with $(m(\tilde{g}),m(\tilde{\chi }^{0}_{2}))=(1095, 205)$ $\text {GeV}$ and $(m(\tilde{g}),m(\tilde{\chi }^{0}_{2}))=(1240, 960)$ $\text {GeV}$, are overlaid. In the case of the $E_{\text {T}}^{\text {miss}}$, $H_{\mathrm {T}}^{\mathrm {incl}}$ and $p_{\text {T}} ^{\ell \ell }$ distributions, the last bin contains the overflow. The flavour-symmetric and $Z/\gamma ^{*}+\text {jets}$ backgrounds are taken from MC simulation and scaled to match their SRZ data-driven predictions. The rare top and data-driven fake-lepton backgrounds are grouped under “other” backgrounds. The *hashed uncertainty bands* include the statistical and systematic uncertainties in the background prediction

**Fig. 8:**
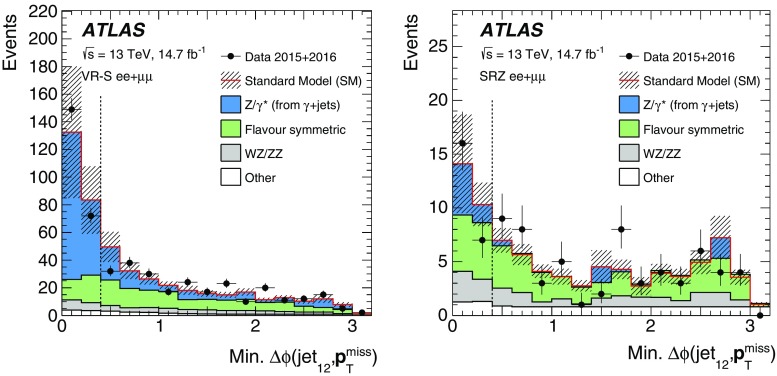
The min. $\Delta \phi (\text {jet}_{12},{\varvec{p}}_{\mathrm {T}}^\mathrm {miss})$ distribution in (*left*) VR-S and (*right*) SRZ, where the min. $\Delta \phi (\text {jet}_{12},{\varvec{p}}_{\mathrm {T}}^\mathrm {miss})>0.4$ requirement has been lifted. The *vertical dashed lines* indicate the requirement in each region. The flavour-symmetric and $Z/\gamma ^{*}+\text {jets}$ distributions are taken completely from the data-driven estimate. The rare top and data-driven fake-lepton backgrounds are grouped under “other” backgrounds. The *hashed uncertainty bands* include the statistical and systematic uncertainties in the background prediction

The dilepton invariant-mass distribution for the $ee+\mu \mu$ and $e\mu$ channels with the kinematic requirements of SRZ, but over the full $m_{\ell \ell }$ range, is shown in Fig. 6. Here the data are consistent with the expected background over the full $m_{\ell \ell }$ range. The dilepton invariant-mass, jet and *b*-tagged jet multiplicity, $E_{\text {T}}^{\text {miss}}$, $H_{\mathrm {T}}^{\mathrm {incl}}$ and $p_{\text {T}} ^{\ell \ell }$ distributions in SRZ are shown in Fig. 7. The shapes of the background distributions in these figures are obtained from MC simulation, where the MC simulation is normalised according to the data-driven estimates in the SR. Here two representative examples of $\tilde{g}$–$\tilde{\chi }_2^0$ on-shell signal models, with $(m(\tilde{g}),m(\tilde{\chi }^{0}_{2}))=(1095, 205)$ $\text {GeV}$ and $(m(\tilde{g}),m(\tilde{\chi }^{0}_{2}))=(1240, 960)$ $\text {GeV}$, are overlaid. To demonstrate the modelling of the $Z/\gamma ^{*}+\text {jets}$ background in VR-S and SRZ, Fig. 8 shows the minimum $\Delta \phi (\text {jet}_{12},{\varvec{p}}_{\mathrm {T}}^\mathrm {miss})$ distribution over the full range, where $\Delta \phi (\text {jet}_{12},{\varvec{p}}_{\mathrm {T}}^\mathrm {miss})>0.4$ is required in VR-S and SRZ. Here the $Z/\gamma ^{*}+\text {jets}$ distribution is modelled using the full data-driven prediction from $\gamma +\text {jets}$. Two of the events in the SR contain a third signal lepton.

### Results in the edge SRs

**Fig. 9:**
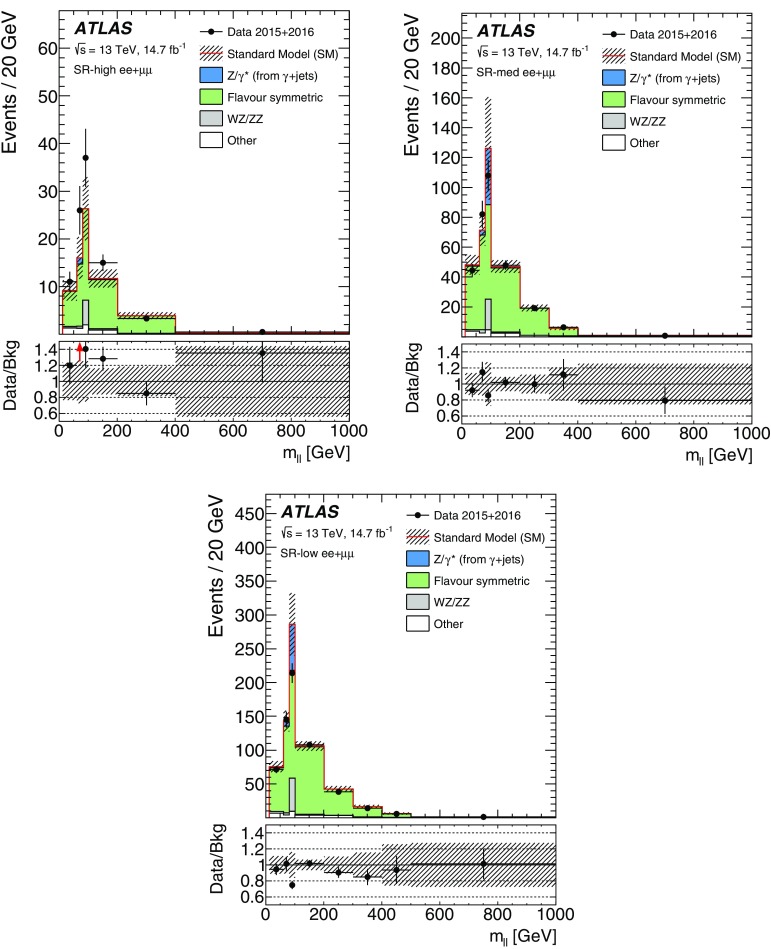
Expected and observed dilepton mass distributions, with the bin boundaries considered for the interpretation, in (*top left*) SR-low, (*top-right*) SR-medium, and (*bottom*) SR-high of the edge search. These bins, and sets of neighbouring bins, make up the mll windows used for the interpretation. The flavour-symmetric and $Z/\gamma ^{*}+\text {jets}$ distributions are taken completely from the data-driven estimate. The rare top and data-driven fake-lepton backgrounds are grouped under “other” backgrounds. All statistical and systematic uncertainties are included in the *hashed bands*. The ratio of data to predicted background is shown in the *bottom panels*. In cases where the data point is not accommodated by the scale of this *panel*, *a red arrow* indicates the direction in which the point is out of range

**Fig. 10:**
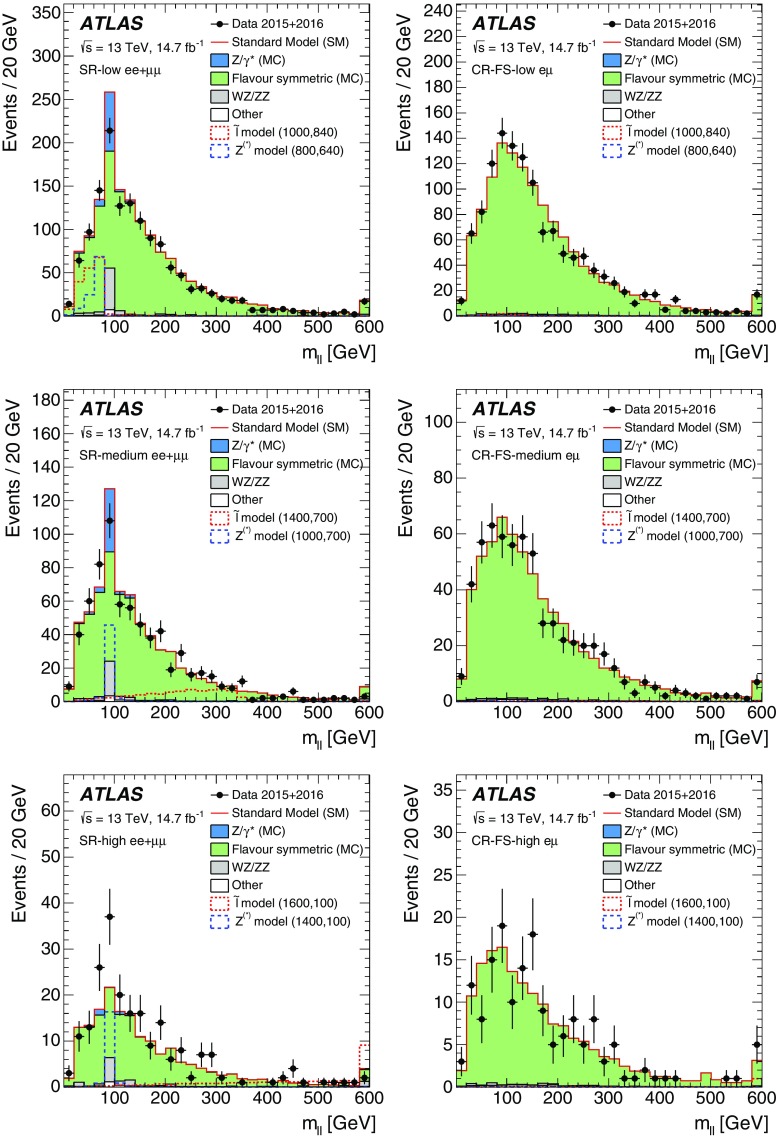
The dilepton mass distributions in the (*top*) SR-low (*left*) and CR-FS-low (*right*), (*middle*) SR-medium (*left*) and CR-FS-medium (*right*), and (*bottom*) SR-high (*left*) and CR-FS-high (*right*) regions of the edge search. The $t\bar{t}$ MC sample is normalised such that the total MC prediction matches data in the $e\mu$ channel for each region. The $m_{\ell \ell }$ shape and normalisation for the $Z/\gamma ^{*}+\text {jets}$ background is taken from the $\gamma +\text {jets}$ method. The rare top and data-driven fake-lepton backgrounds are grouped under “other” backgrounds. Example signal benchmarks from the slepton and $Z^{(*)}$ models are overlaid on the distributions. The first (second) number in *parentheses* is the gluino (LSP) mass. The overflow is included in the last bin

The integrated yields in the edge signal regions are compared to the expected background in Table 10. To allow for the visualisation of a potential edge, the full $m_{\ell \ell }$ distributions in the three search regions are compared to the expected background in Fig. 9. In addition, the observed $m_{\ell \ell }$ distributions are compared to the predictions from MC simulation in Fig. 10, in which the $t\bar{t}$ background is scaled such that the total MC expected yield matches the data in the $e\mu$ CR. The $t\bar{t}$ normalisation factors are $\mu _{t\bar{t}}=0.85\pm 0.03$, $0.75\pm 0.04$, and $0.57\pm 0.07$ in SR-low, SR-medium, and SR-high, respectively, where the uncertainty is the data statistical uncertainty. The data-driven flavour-symmetry prediction is used for the quantitative results of the analysis. This prediction does not rely on the $t\bar{t}$ normalisation scale factors discussed above. The MC-based cross-check method is used to examine the $m_{\ell \ell }$ distribution in finer bins than can be achieved with the flavour-symmetry method, due to the limited statistical precision of the $e\mu$ CR.

**Table 10:** Breakdown of the expected background and observed data yields for SR-low, SR-medium and SR-high, integrated over the $m_{\ell \ell }$ spectrum. The flavour-symmetric, $Z/\gamma ^{*}+\text {jets}$ and fake-lepton components are all derived using data-driven estimates described in Sect. 7. All remaining backgrounds are taken from MC simulation. The quoted uncertainties include statistical and systematic contributions

	SR-low	SR-medium	SR-high
Observed events	1394	689	212
Total expected background events	$1500 \pm 100$	$700 \pm 60$	$171 \pm 18$
Flavour-symmetric ($t\bar{t}$, *Wt*, *WW* and $Z\rightarrow \tau \tau$) events	$1270 \pm \text{[ }3.5ex]{70 }$	$584 \pm 32$	$148 \pm 14$
$Z/\gamma ^{*}+\text {jets}$ events	${90} \pm {50 }$	${50} \pm {40 }$	$3^{+7}_{-3}$
*WZ* / *ZZ* events	${68} \pm {31 }$	${26} \pm {11 }$	${7} \pm {4 }$
Rare top events	$19 \pm 5$	$11.3 \pm 3.2$	${4.2} \pm {1.4 }$
Fake-lepton events	${59} \pm {34 }$	${32} \pm {19 }$	$10 \pm 8$

**Fig. 11:**
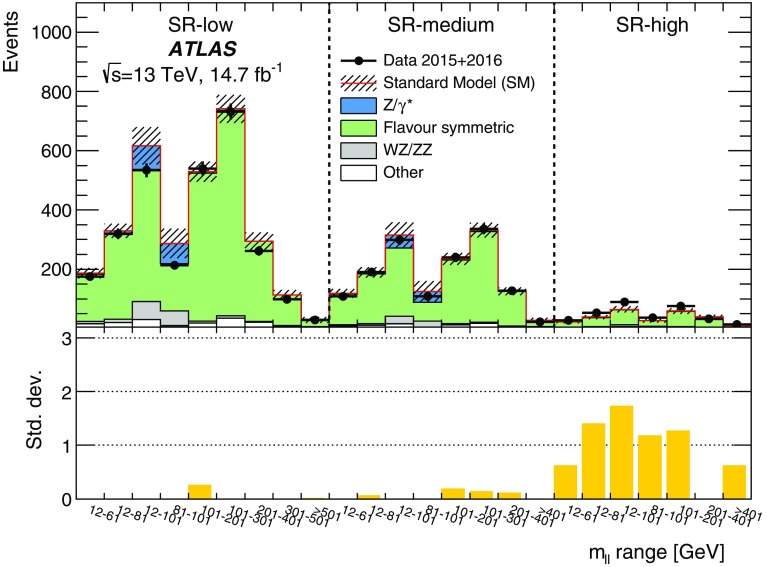
The expected and observed yields in the 24 (overlapping) $m_{\ell \ell }$ ranges of SR-low, SR-medium, and SR-high. The data are compared to the sum of the expected backgrounds. The rare top and data-driven fake-lepton backgrounds are grouped under “other” backgrounds. The significance of the difference between the data and the expected background (see *text* for details) is shown in the *bottom plots*; for regions in which the data yield is less than expected, the significance is set to zero. The *hashed uncertainty bands* include the statistical and systematic uncertainties in the background prediction

As signal models may produce kinematic endpoints at any value of $m_{\ell \ell }$, any excess must be searched for across the $m_{\ell \ell }$ distribution. To do this a “sliding window” approach is used. The binning in the SRs, shown in Fig. 9, defines many possible dilepton mass windows. The 24 $m_{\ell \ell }$ ranges (9 for SR-low, 8 for SR-medium, and 7 for SR-high) are chosen because they are the most sensitive for at least one grid point in the signal model parameter space. Some of the ranges overlap. The results in these regions are summarised in Fig. 11, and the expected and observed yields in the combined $ee+\mu \mu$ channel for all 24 $m_{\ell \ell }$ ranges are presented in Table 11. In SR-low and SR-medium, the data are consistent with the expected background across the full $m_{\ell \ell }$ range. In SR-high the data show a slight excess above the background at low $m_{\ell \ell }$. Of these 24 $m_{\ell \ell }$ ranges, the largest excess is observed in SR-high with $12<m_{\ell \ell }<101$ $\text {GeV}$. Here a total of 90 events are observed in data, compared to an expectation of $65\pm 10$ events, corresponding to a local significance of $1.7\sigma$.

**Table 11:** Breakdown of the expected background and observed data yields in the edge signal regions. The results are given for SR-low, SR-medium and SR-high in all 24 $m_{\ell \ell }$ ranges. The $m_{\ell \ell }$ range in units of $\text {GeV}$ is indicated in the leftmost column of the table. Left to right: the total expected background, with combined statistical and systematic uncertainties, observed data, 95% CL upper limits on the visible cross section ($\langle \epsilon \sigma \rangle _\mathrm{obs}^{95}$) and on the number of signal events ($S_\mathrm{obs}^{95}$). The sixth column ($S_\mathrm{exp}^{95}$) shows the expected 95% CL upper limit on the number of signal events, given the expected number (and $\pm 1\sigma$ excursions) of background events. The last two columns indicate the discovery *p* value ($p(s = 0)$) [97], and the Gaussian significance ($Z(s=0)$). For an observed number of events lower than expected, the discovery *p* value is truncated at 0.5 and the significance is set to zero

Signal region	Total Bkg.	Data	$\langle \epsilon \mathrm{\sigma }\rangle _\mathrm{obs}^{95}$[fb]	$S_\mathrm{obs}^{95}$	$S_\mathrm{exp}^{95}$	$p(s=0)$	$Z(s=0)$
SR-low							
12–61	$$187\pm 18 $$	175	2.68	39.4	$$ { 48 }^{ +23 }_{ -14 }$$	0.50	0.00
12–81	$$330\pm 24 $$	320	3.88	57.1	$$ { 64 }^{ +30 }_{ -19 }$$	0.50	0.00
12–101	$$617\pm 63 $$	534	4.64	68.2	$$ { 98 }^{ +36 }_{ -26 }$$	0.50	0.00
81–101	$$287\pm 50 $$	214	2.73	40.2	$$ { 62 }^{ +22 }_{ -16 }$$	0.50	0.00
101–201	$$529\pm 34 $$	540	6.80	99.9	$$ { 91 }^{ +52 }_{ -29 }$$	0.40	0.26
101–301	$$741\pm 48 $$	732	7.28	107	$$ { 113 }^{ +53 }_{ -33 }$$	0.50	0.00
201–401	$$295\pm 30 $$	262	3.43	50.5	$$ { 70 }^{ +37 }_{ -21 }$$	0.50	0.00
301–501	$$113\pm 17 $$	99	2.37	34.8	$$ { 46 }^{ +41 }_{ -16 }$$	0.50	0.00
>501	$$29\pm 10 $$	29	1.88	27.7	$$ { 27 }^{ +34 }_{ -10 }$$	0.50	0.01
SR-medium							
12–61	$$119\pm 15 $$	109	2.38	35.1	$$ { 43 }^{ +29 }_{ -14 }$$	0.50	0.00
12–81	$$190\pm 18 $$	191	3.57	52.5	$$ { 51 }^{ +31 }_{ -15 }$$	0.48	0.06
12–101	$$315\pm 43 $$	299	5.12	75.3	$$ { 81 }^{ +29 }_{ -20 }$$	0.50	0.00
81–101	$$125\pm 35 $$	108	3.18	46.7	$$ { 51 }^{ +17 }_{ -12 }$$	0.50	0.00
101–201	$$235\pm 20 $$	240	4.26	62.6	$$ { 58 }^{ +37 }_{ -19 }$$	0.42	0.19
101–301	$$332\pm 25 $$	336	4.92	72.3	$$ { 69 }^{ +39 }_{ -22 }$$	0.45	0.14
201–401	$$126\pm 13 $$	128	3.27	48.0	$$ { 46 }^{ +52 }_{ -16 }$$	0.46	0.11
>401	$$28\pm 8 $$	22	1.09	16.1	$$ { 21 }^{ +19 }_{ -7 }$$	0.50	0.00
SR-high							
12–61	$$23\pm 5 $$	27	1.84	27.0	$$ { 20 }^{ +31 }_{ -8 }$$	0.27	0.62
12–81	$$39\pm 7 $$	53	3.32	48.9	$$ { 26 }^{ +28 }_{ -10 }$$	0.08	1.40
12–101	$$ {65}\pm 10 $$	90	4.00	58.8	$$ { 31 }^{ +17 }_{ -10 }$$	0.04	1.73
81–101	$$26\pm 6 $$	37	2.17	31.9	$$ { 20 }^{ +13 }_{ -7 }$$	0.12	1.18
101–201	$$59\pm 9 $$	75	3.68	54.1	$$ { 31 }^{ +29 }_{ -11 }$$	0.10	1.27
201–401	$$39\pm 7 $$	33	1.82	26.7	$$ { 28 }^{ +14 }_{ -7 }$$	0.50	0.00
>401	$$10\pm 5 $$	14	2.04	30.0	$$ { 21 }^{ +79 }_{ -10 }$$	0.27	0.62

## Interpretation

In this section, exclusion limits are shown for the SUSY models detailed in Sect. 3. The asymptotic $CL_{\text {S}}$ prescription [90, 98], implemented in the HistFitter program [97], is used to determine cross-section upper limits at $95\%$ confidence level (CL) for the on-*Z* search. For the edge search, pseudo-experiments are used to evaluate the cross-section upper limits. A Gaussian model for nuisance parameters is used for all signal and background uncertainties. Exceptions are the statistical uncertainties of the flavour-symmetry method, $\gamma +\text {jets}$ method and MC-based backgrounds, all of which are treated as Poissonian nuisance parameters. The different experimental uncertainties are treated as correlated between signal and background events. The theoretical uncertainty of the signal cross section is not accounted for in the limit-setting procedure. Instead, following the initial limit determination, the impact of varying the signal cross section within its uncertainty is evaluated separately and indicated in the exclusion results. Limits are based on the combined $ee+\mu \mu$ results. Possible signal contamination in the CRs is neglected in the limit-setting procedure; the contamination is found to be negligible for signal points near the exclusion boundaries. Far from the exclusion boundary, although the signal contamination can be significant, the number of events appearing in the signal region is large enough that the points are still excluded, due to the relative branching fractions for the signal in the CR and SR. For example, for models with signal contamination of 50% in CR-FS the signal-to-background ratio in SRZ is $\sim$10.

**Fig. 12:**
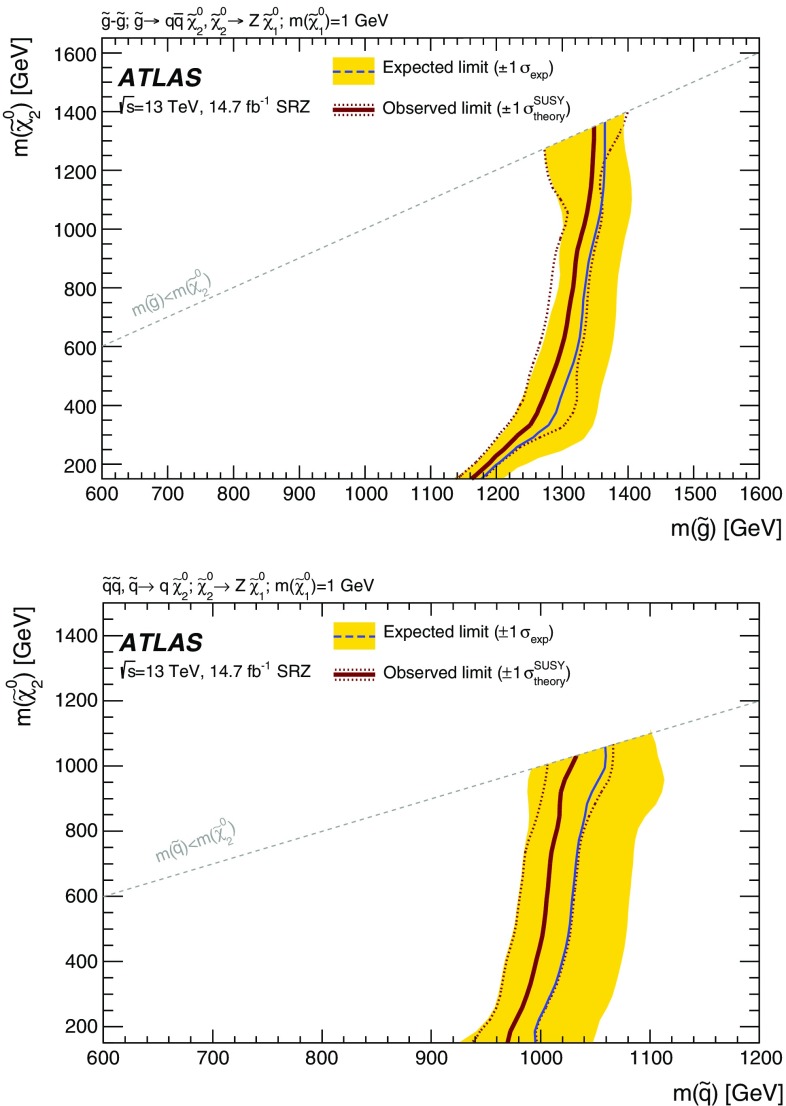
Expected and observed exclusion contours derived from the results in SRZ for the (*top*) $\tilde{g}$–$\tilde{\chi }_2^0$ on-shell grid and (*bottom*) $\tilde{q}$–$\tilde{\chi }_2^0$ on-shell grid. The *dashed blue line* indicates the expected limits at $95\%$ CL and the *yellow band* shows the $1\sigma$ variation of the expected limit as a consequence of the uncertainties in the background prediction and the experimental uncertainties in the signal ($\pm 1\sigma _\text {exp}$). The observed limits are shown by the *solid red line*, with the *dotted red lines* indicating the variation resulting from changing the signal cross section within its uncertainty ($\pm 1\sigma ^\text {SUSY}_\text {theory}$)

The results of the on-shell *Z* search are interpreted in a simplified model with gluino-pair production, where each gluino decays as $\tilde{g} \rightarrow q\bar{q} \tilde{\chi }^{0}_{2}, \tilde{\chi }^{0}_{2} \rightarrow Z \tilde{\chi }^{0}_{1}$ and the $\tilde{\chi }^{0}_{1}$ mass is set to 1 $\text {GeV}$. The expected and observed exclusion contours for this $\tilde{g}$–$\tilde{\chi }_2^0$ on-shell grid are shown in the $m(\tilde{g})$–$m(\tilde{\chi }^{0}_{2})$ plane in Fig. 12. The expected (observed) lower limit on the gluino mass is about 1.35 $\text {TeV}$ (1.30 $\text {TeV}$) for a $\tilde{\chi }_2^0$ with a mass of 1.1 $\text {TeV}$ in this model. The impact of the systematic uncertainties in the background and the experimental uncertainties in the signal, shown with a coloured band, is about 100 $\text {GeV}$ on the gluino mass limit. The systematic uncertainty of the signal cross section, shown as dotted lines around the observed contour, has an impact of about 40 $\text {GeV}$. Figure 12 also shows the expected and observed exclusion limits for the $\tilde{q}$–$\tilde{\chi }_2^0$ on-shell model. This is a simplified model with squark-pair production, where each squark decays to a quark and a neutralino, with the neutralino subsequently decaying to a *Z* boson and an LSP with a mass of 1 $\text {GeV}$. In this model, exclusion is expected (observed) for squarks with masses below 1040 $\text {GeV}$ (980 $\text {GeV}$) for a $\tilde{\chi }^{0}_{2}$ mass of 600 $\text {GeV}$.

**Fig. 13:**
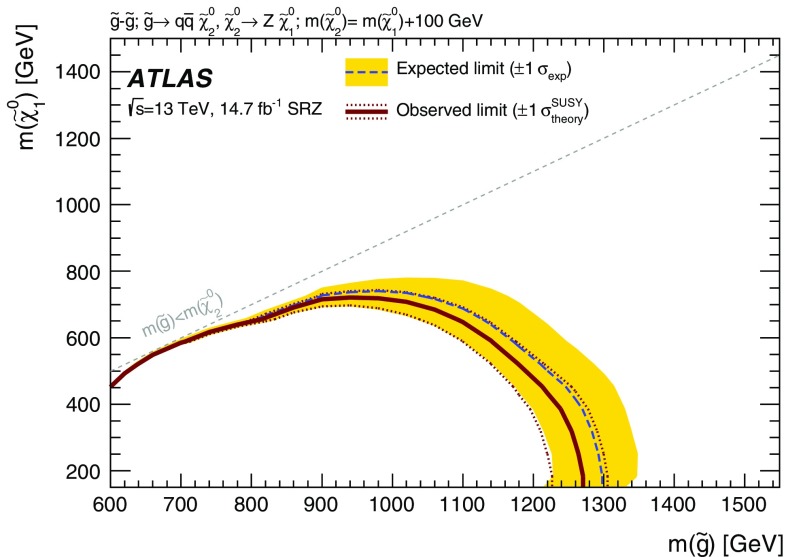
Expected and observed exclusion contours derived from the results in SRZ for the $\tilde{g}$–$\tilde{\chi }_1^0$ on-shell grid. The *dashed blue line* indicates the expected limits at $95\%$ CL and the *yellow band* shows the $1\sigma$ variation of the expected limit as a consequence of the uncertainties in the background prediction and the experimental uncertainties in the signal ($\pm 1\sigma _\text {exp}$). The observed limits are shown by the *solid red line*, with the *dotted red lines* indicating the variation resulting from changing the signal cross section within its uncertainty ($\pm 1\sigma ^\text {SUSY}_\text {theory}$)

Figure 13 shows the expected and observed exclusion contours for the $\tilde{g}$–$\tilde{\chi }_1^0$ on-shell model, in which the produced gluinos follow the same decay chain as in the model above. In this case the mass difference $\Delta m = m(\tilde{\chi }^{0}_{2})-m(\tilde{\chi }^{0}_{1})$ is set to 100 $\text {GeV}$.

The results of the edge search are interpreted in two simplified models with gluino-pair production, in which each gluino decays as $\tilde{g} \rightarrow q\bar{q} \tilde{\chi }^{0}_{2}$. For each point in the signal-model parameter space, limits on the signal strength are calculated using the $m_{\ell \ell }$ window with the best expected sensitivity. Details of the windows are described in Sect. 9.

**Fig. 14:**
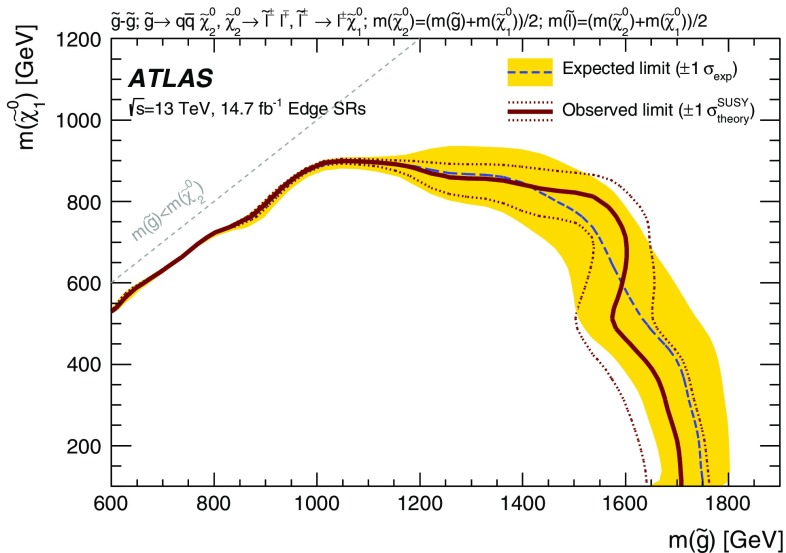
Expected and observed exclusion contours derived from the results in the edge search SRs for the slepton signal model. The *dashed blue line* indicates the expected limits at $95\%$ CL and the *yellow band* shows the $1\sigma$ variation of the expected limit as a consequence of the uncertainties in the background prediction and the experimental uncertainties in the signal ($\pm 1\sigma _\text {exp}$). The observed limits are shown by the *solid red lines*, with the *dotted red lines* indicating the variation resulting from changing the signal cross section within its uncertainty ($\pm 1\sigma ^\text {SUSY}_\text {theory}$)

The excluded regions in the $m(\tilde{g})$–$m(\tilde{\chi }^{0}_{1})$ plane are presented in Fig. 14 for the slepton model. In this model, pair-produced gluinos each decay as $\tilde{g}\rightarrow q \bar{q} \tilde{\chi }^{0}_2, \tilde{\chi }^{0}_2 \rightarrow \ell ^\pm \tilde{\ell }^\mp , \tilde{\ell }^\mp \rightarrow \ell ^\mp \tilde{\chi }^{0}_{1}$. Here, the results exclude gluinos with masses as large as 1.7 $\text {TeV}$, with an expected limit of 1.75 $\text {TeV}$ for small $m(\tilde{\chi }^{0}_{1})$. The results probe kinematic endpoints as small as $m_{\ell \ell }^{\text {max}} = m(\tilde{\chi }^{0}_{2})-m(\tilde{\chi }^{0}_{1}) = 1/2(m(\tilde{g})-m(\tilde{\chi }^{0}_{1}) ) = 50$ $\text {GeV}$.

**Fig. 15:**
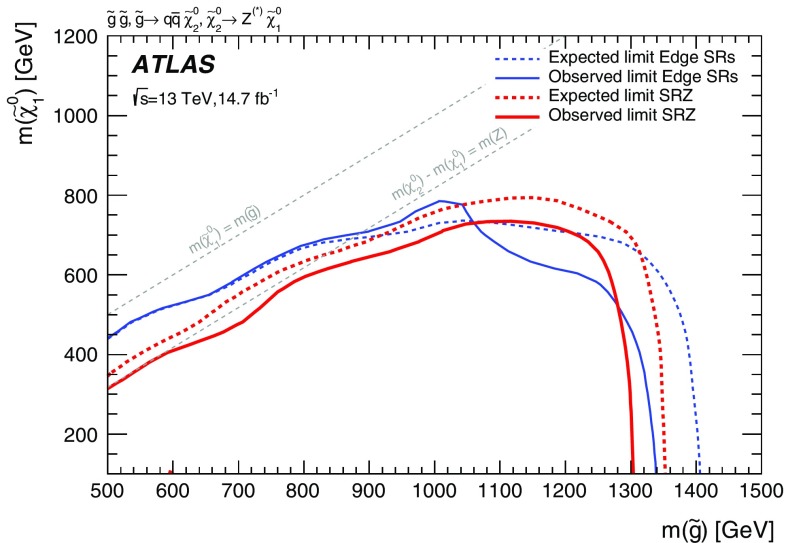
Expected and observed exclusion contours derived from the results in the edge search SRs and SRZ for the $Z^{(*)}$ model. The *dashed* and *solid blue lines* indicate the expected and observed limits at $95\%$ CL from the results in the edge SRs, while the *thick dashed* and *solid red lines* indicate the expected and observed limits at $95\%$ CL from the results in SRZ

The $Z^{(*)}$ exclusion limits from the results in the edge SRs are compared with the same limits derived using the results in SRZ in Fig. 15. In this model, pair-produced gluinos each decay as $\tilde{g} \rightarrow q\bar{q} \tilde{\chi }^{0}_{2}, \tilde{\chi }^{0}_{2} \rightarrow Z^{(*)} \tilde{\chi }^{0}_{1}$, and the mass splitting between the $\tilde{\chi }^{0}_{2}$ and the $\tilde{\chi }^{0}_{1}$ determines whether the *Z* boson is produced on-shell. Here the edge limits extend into the more compressed region, whereas the expected SRZ exclusion probes higher $\tilde{\chi }^{0}_{1}$ masses in the on-shell regime. At high gluino masses, the edge SRs provide stronger limits. For the $Z^{(*)}$ model, the expected and observed gluino mass limits are 1.4 $\text {TeV}$ and 1.34 $\text {TeV}$ (1.35 and 1.3 $\text {TeV}$ for the on-Z signal region), respectively, for $\tilde{\chi }_1^0$ masses below 400 $\text {GeV}$. The sensitivity in the $Z^{(*)}$ model is smaller than that of the slepton model because the leptonic branching fraction of the *Z* boson suppresses the signal production rate.

Model-independent upper limits at 95% CL on the number of events that could be attributed to non-SM sources ($S^{95}$) for SRZ are derived using the $CL_{\text {S}}$ prescription and neglecting possible signal contamination in the CRs. For these upper limits, pseudo-experiments are used rather than the asymptotic approximation. The expected and observed upper limits are given in Table 9. The same information is given for the 24 $m_{\ell \ell }$ ranges of the edge search in Table 11.

## Conclusion

This paper presents two searches for new phenomena in final states containing a same-flavour opposite-sign lepton (electron or muon) pair, jets, and large missing transverse momentum using $14.7~\mathrm {fb}^{-1}$ of ATLAS data collected during 2015 and 2016 at the LHC at $\sqrt{s}=13$ $\text {TeV}$. The first search (on-shell *Z* search) targets lepton pairs consistent with *Z* boson decay, while the second search (edge search) targets a kinematic endpoint feature in the dilepton mass distribution. For the edge search, a set of 24 mass ranges are considered, with different requirements on $E_{\text {T}}^{\text {miss}}$ and $H_{\text {T}}$, and different kinematic endpoint values in the dilepton invariant-mass distribution. The data in both searches are found to be consistent with the Standard Model prediction. The results are interpreted in simplified models of gluino-pair production and squark-pair production, and exclude gluinos (squarks) with masses as large as 1.7 $\text {TeV}$ (980 $\text {GeV}$).

## Footnotes

1 ATLAS uses a right-handed coordinate system with its origin at the nominal interaction point (IP) in the centre of the detector and the *z*-axis along the beam pipe. The *x*-axis points from the IP to the centre of the LHC ring, and the *y*-axis points upward. Cylindrical coordinates $(r,\phi )$ are used in the transverse plane, $\phi$ being the azimuthal angle around the *z*-axis. The pseudorapidity is defined in terms of the polar angle $\theta$ as $\eta =-\ln \tan (\theta /2)$ and the rapidity is defined as $y=1/2 \cdot \ln [(E+p_{z})/(E-p_{z})])$, where *E* is the energy and $p_{z}$ the longitudinal momentum of the object of interest. The opening angle between two analysis objects in the detector is defined as $\Delta R=\sqrt{(\Delta y)^2+(\Delta \phi )^2}$.

2 The distance of closest approach between a particle object and the primary vertex (beamline) in the longitudinal (transverse) plane is denoted by $z_0$ ($d_0$).

## References

[1] Golfand YA, Likhtman EP (1971). Extension of the algebra of Poincare group generators and violation of p invariance. JETP Lett..

[2] Golfand YA, Likhtman EP (1971). Pisma. Zh. Eksp. Teor. Fiz..

[3] Volkov DV, Akulov VP (1973). Is the neutrino a goldstone particle?. Phys. Lett. B.

[4] Wess J, Zumino B (1974). Supergauge transformations in four-dimensions. Nucl. Phys. B.

[5] Wess J, Zumino B (1974). Supergauge invariant extension of quantum electrodynamics. Nucl. Phys. B.

[6] Ferrara S, Zumino B (1974). Supergauge invariant Yang–Mills theories. Nucl. Phys. B.

[7] Salam A, Strathdee JA (1974). Supersymmetry and nonabelian gauges. Phys. Lett. B.

[8] Sakai N (1981). Naturalness in supersymmetric GUTS. Z. Phys. C.

[9] Dimopoulos S, Raby S, Wilczek F (1981). Supersymmetry and the scale of unification. Phys. Rev. D.

[10] Ibanez LE, Ross GG (1981). Low-energy predictions in supersymmetric grand unified theories. Phys. Lett. B.

[11] Dimopoulos S, Georgi H (1981). Softly broken supersymmetry and SU(5). Nucl. Phys. B.

[12] Farrar GR, Fayet P (1978). Phenomenology of the production, decay, and detection of new hadronic states associated with supersymmetry. Phys. Lett. B.

[13] H. Goldberg, Constraint on the photino mass from cosmology. Phys. Rev. Lett. **50**, 1419 (1983). doi:10.1103/PhysRevLett.50.1419. [Erratum: Phys. Rev. Lett. 103, 099905 (2009)]

[14] Ellis JR (1984). Supersymmetric relics from the big bang. Nucl. Phys. B.

[15] Dine M, Fischler W (1982). A Phenomenological model of particle physics based on supersymmetry. Phys. Lett. B.

[16] Alvarez-Gaume L, Claudson M, Wise MB (1982). Low-energy supersymmetry. Nucl. Phys. B.

[17] Nappi CR, Ovrut BA (1982). Supersymmetric extension of the SU(3) x SU(2) x U(1) model. Phys. Lett. B.

[18] CMS Collaboration, Search for physics beyond the standard model in events with a $$Z$$ boson, jets, and missing transverse energy in $$pp$$ collisions at $$\sqrt{s}=7$$ TeV. Phys. Lett. B **716**, 260–284 (2012). doi:10.1016/j.physletb.2012.08.026. arXiv:1204.3774 [hep-ex]

[19] CMS Collaboration, Search for physics beyond the standard model in events with two leptons, jets, and missing transverse momentum in pp collisions at $$\sqrt{s}$$ = 8 TeV. JHEP **04**, 124 (2015). doi:10.1007/JHEP04(2015)124. arXiv:1502.06031 [hep-ex]

[20] ATLAS Collaboration, Search for supersymmetry in events containing a same-flavour opposite-sign dilepton pair, jets, and large missing transverse momentum in $$\sqrt{s} = 8\;TeV$$$$pp$$ collisions with the ATLAS detector. Eur. Phys. J. C **75**, 318 (2015). doi:10.1140/epjc/s10052-015-3518-2. arXiv:1503.03290 [hep-ex] 10.1140/epjc/s10052-015-3518-2 PMC4498693 26190940

[21] CMS Collaboration, Search for new physics in final states with two opposite-sign same-flavor leptons, jets and missing transverse momentum in pp collisions at $$\sqrt{s}=13$$ TeV. JHEP **12**, 013 (2016). doi:10.1007/JHEP12(2016)013. arXiv:1607.00915 [hep-ex]

[22] ATLAS Collaboration, Search for new physics in events with opposite-sign leptons, jets, and missing transverse energy in $$pp$$ collisions at $$\sqrt{s}=7$$ TeV. Phys. Lett. B **718**, 815–840 (2013). doi:10.1016/j.physletb.2012.11.036. arXiv:1206.3949 [hep-ex]

[23] ATLAS Collaboration, The ATLAS experiment at the CERN large hadron collider. JINST. **3**, S08003 (2008). doi:10.1088/1748-0221/3/08/S08003

[24] ATLAS Collaboration, Early inner detector tracking performance in the 2015 data at $$\sqrt{s}$$ = 13 TeV. ATL-PHYS-PUB-2015-051, 2015. http://cds.cern.ch/record/2110140

[25] Fayet P (1976). Supersymmetry and weak, electromagnetic and strong interactions. Phys. Lett. B.

[26] Fayet P (1977). Spontaneously broken supersymmetric theories of weak, electromagnetic and strong interactions. Phys. Lett. B.

[27] M. Cahill-Rowley et al., ATLAS Z+ missing transverse energy excess in the MSSM. Phys. Rev. D **92**, 075029 (2015). doi:10.1103/PhysRevD.92.075029. arXiv:1506.05799 [hep-ph]

[28] ATLAS Collaboration, Search for direct top squark pair production in events with a $$Z$$ boson, $$b$$-jets and missing transverse momentum in $$\sqrt{s} = 8\;TeV$$$$pp$$ collisions with the ATLAS detector. Eur. Phys. J. C **74**, 2883 (2014). doi:10.1140/epjc/s10052-014-2883-6. arXiv:1403.5222 [hep-ex] 10.1140/epjc/s10052-014-2883-6 PMC4370855 25814893

[29] ATLAS Collaboration, Search for direct top-squark pair production in final states with two leptons in $$pp$$ collisions at $$\sqrt{s} = 8\;TeV$$ with the ATLAS detector. JHEP **06**, 124 (2014). doi:10.1007/JHEP06(2014)124. arXiv:1403.4853 [hep-ex]

[30] D. Alves, E. Izaguirre, J. Wacker, Where the sidewalk ends: jets and missing energy search strategies for the 7 TeV LHC. JHEP **10**, 012 (2011). doi:10.1007/JHEP10(2011)012. arXiv:1102.5338 [hep-ph]

[31] ATLAS Collaboration, Improved luminosity determination in pp collisions at $$\sqrt{s}$$ = 7 TeV using the ATLAS detector at the LHC. Eur. Phys. J. C **73**, 2518 (2013). doi:10.1140/epjc/s10052-013-2518-3. arXiv:1302.4393 [hep-ex] 10.1140/epjc/s10052-013-2518-3 PMC4370906 25814867

[32] ATLAS Collaboration, Luminosity determination in pp collisions at $$\sqrt{s}=8$$ TeV using the ATLAS detector at the LHC. Eur. Phys. J. C **76**, 653 (2016). doi:10.1140/epjc/s10052-016-4466-1. arXiv:1608.03953 [hep-ex] 10.1140/epjc/s10052-016-4466-1 PMC5335615 28316496

[33] ATLAS Collaboration, 2015 start-up trigger menu and initial performance assessment of the ATLAS trigger using Run-2 data. ATL-DAQ-PUB-2016-001, 2016. http://cds.cern.ch/record/2136007

[34] J. Alwall et al., The automated computation of tree-level and next-to-leading order differential cross sections, and their matching to parton shower simulations. JHEP **07**, 079 (2014). doi:10.1007/JHEP07(2014)079. arXiv:1405.0301 [hep-ph]

[35] T. Sjöstrand, S. Mrenna, P. Skands, A brief introduction to PYTHIA 8.1. Comput. Phys. Commun. **178**, 852 (2008). doi:10.1016/j.cpc.2008.01.036. arXiv:0710.3820 [hep-ph]

[36] ATLAS Collaboration, ATLAS Pythia 8 tunes to 7 TeV data. ATL-PHYS-PUB-2014-021, 2014. http://cdsweb.cern.ch/record/1966419

[37] R.D. Ball et al., Parton distributions with LHC data. Nucl. Phys. B **867**, 244–289 (2013). doi:10.1016/j.nuclphysb.2012.10.003. arXiv:1207.1303 [hep-ph]

[38] P. Nason, A new method for combining NLO QCD with shower Monte Carlo algorithms. JHEP **11**, 040 (2004). arXiv:hep-ph/0409146 [hep-ph]

[39] S. Frixione, P. Nason, C. Oleari, Matching NLO QCD computations with parton shower simulations: the POWHEG method. JHEP **11**, 070 (2007). arXiv:0709.2092 [hep-ph]

[40] S. Alioli et al., A general framework for implementing NLO calculations in shower Monte Carlo programs: the POWHEG BOX. JHEP **06**, 043 (2010). arXiv:1002.2581 [hep-ph]

[41] T. Sjöstrand, S. Mrenna, P. Skands, PYTHIA 6.4 physics and manual. JHEP **05**, 026 (2006). doi:10.1088/1126-6708/2006/05/026. arXiv:hep-ph/0603175 [hep-ph]

[42] B. Cooper et al., Monte Carlo tuning in the presence of matching. Eur. Phys. J. C **72**, 2078 (2011). doi:10.1140/epjc/s10052-012-2078-y. arXiv:1109.5295 [hep-ph]

[43] Lange DJ (2001). The EvtGen particle decay simulation package. Nucl. Instrum. Methods A.

[44] T. Gleisberg et al., Event generation with Sherpa 1.1. JHEP **02**, 007 (2009). arXiv:0811.4622 [hep-ph]

[45] S. Schumann, F. Krauss, A Parton shower algorithm based on Catani–Seymour dipole factorisation. JHEP **03**, 038 (2008). doi:10.1088/1126-6708/2008/03/038. arXiv:0709.1027 [hep-ph]

[46] T. Gleisberg, S. Höche, Comix, a new matrix element generator. JHEP **12**, 039 (2008). doi:10.1088/1126-6708/2008/12/039. arXiv:0808.3674 [hep-ph]

[47] F. Cascioli, P. Maierhofer, S. Pozzorini, Scattering amplitudes with open loops. Phys. Rev. Lett. **108**, 111601 (2012). doi:10.1103/PhysRevLett.%20108.111601. arXiv:1111.5206 [hep-ph] 10.1103/PhysRevLett.108.111601 22540459

[48] S. Höche et al., QCD matrix elements + parton showers: the NLO case. JHEP **04**, 027 (2013). doi:10.1007/JHEP04(2013)%20027. arXiv:1207.5030 [hep-ph]

[49] S. Catani et al., QCD Matrix elements + Parton showers. JHEP **11**, 063 (2001). doi:10.1088/1126-6708/2001/11/063. arXiv:hep-ph/0109231

[50] L. Lönnblad, Correcting the colour-dipole cascade model with fixed order matrix elements. JHEP **05**, 046 (2002). doi:10.1088/1126-6708/2002/05/046. arXiv:hep-ph/0112284

[51] W. Beenakker et al., Squark and gluino production at hadron colliders. Nucl. Phys. B **492**, 51–103 (1997). doi:10.1016/S0550-3213(97)00084-9. arXiv:hep-ph/9610490 [hep-ph]

[52] A. Kulesza, L. Motyka, Threshold resummation for squark–antisquark and gluino-pair production at the LHC. Phys. Rev. Lett. **102**, 111802 (2009). doi:10.1103/PhysRevLett.102.111802. arXiv:0807.2405 [hep-ph] 10.1103/PhysRevLett.102.111802 19392192

[53] A. Kulesza, L. Motyka, Soft gluon resummation for the production of gluino–gluino and squark–antisquark pairs at the LHC. Phys. Rev. D **80**, 095004 (2009). doi:10.1103/PhysRevD.80.095004. arXiv:0905.4749 [hep-ph] 10.1103/PhysRevLett.102.111802 19392192

[54] W. Beenakker et al., Soft-gluon resummation for squark and gluino hadroproduction. JHEP **12**, 041 (2009). doi:10.1088/1126-6708/2009/12/041. arXiv:0909.4418 [hep-ph]

[55] W. Beenakker et al., Squark and gluino hadroproduction. Int. J. Mod. Phys. A **26**, 2637–2664 (2011). doi:10.1142/S0217751X11053560. arXiv:1105.1110 [hep-ph]

[56] ATLAS Collaboration, The ATLAS simulation infrastructure. Eur. Phys. J. C **70**, 823–874 (2010). doi:10.1140/epjc/s10052-010-1429-9. arXiv:1005.4568 [physics.ins-det]

[57] Agostinelli S (2003). GEANT4: a simulation toolkit. Nucl. Instrum. Methods A.

[58] ATLAS Collaboration, Summary of ATLAS Pythia 8 tunes. ATL-PHYS-PUB-2012-003, 2012. http://cds.cern.ch/record/1474107

[59] G. Watt, R.S. Thorne, Study of Monte Carlo approach to experimental uncertainty propagation with MSTW 2008 PDFs. JHEP **08**, 052 (2012). doi:10.1007/JHEP08(2012)052. arXiv:1205.4024 [hep-ph]

[60] ATLAS Collaboration, Modelling of the $$t\bar{t}H$$ and $$t\bar{t}V$$$$(V=W,Z)$$ processes for $$\sqrt{s}=13$$ TeV ATLAS analyses. ATL-PHYS-PUB-2016-005, 2016. http://cds.cern.ch/record/2120826

[61] M.V. Garzelli et al., $$t\bar{t}$$$$W^{+-}$$ and $$t\bar{t}Z$$ Hadroproduction at NLO accuracy in QCD with Parton Shower and Hadronization effects. JHEP **11**, 056 (2012). doi:10.1007/JHEP11(2012)056. arXiv:1208.2665 [hep-ph]

[62] J.M. Campbell, R.K. Ellis, $$t\bar{t}W$$ production and decay at NLO. JHEP **07**, 052 (2012). arXiv:1204.5678 [hep-ph]

[63] A. Lazopoulos et al., Next-to-leading order QCD corrections to $$t\bar{t}Z$$ production at the LHC. Phys Lett. B **666**, 62 (2008). arXiv:0804.2220 [hep-ph]

[64] ATLAS Collaboration, Simulation of top quark production for the ATLAS experiment at $$\sqrt{s}$$ = 13 TeV. ATL-PHYS-PUB-2016-004, 2016. http://cds.cern.ch/record/2120417

[65] M. Czakon, P. Fiedler, A. Mitov, Total top-quark pair-production cross section at hadron colliders through $$O(\alpha _s^4)$$. Phys. Rev. Lett. **110**, 252004 (2013). arXiv:1303.6254 [hep-ph] 10.1103/PhysRevLett.110.252004 23829732

[66] M. Czakon, A. Mitov, Top++: a program for the calculation of the top-pair cross-section at hadron colliders. Comput. Phys. Commun. **185**, 2930 (2014). doi:10.1016/j.cpc.2014.06.021. arXiv:1112.5675 [hep-ph]

[67] N. Kidonakis, Two-loop soft anomalous dimensions for single top quark associated production with a $$W^-$$ or $$H^-$$. Phys. Rev. D **82**, 054018 (2010). doi:10.1103/PhysRevD.82.054018. arXiv:1005.4451 [hep-ph]

[68] ATLAS Collaboration, Multi-Boson simulation for 13 TeV ATLAS analyses. ATL-PHYS-PUB-2016-002, 2016. http://cds.cern.ch/record/2119986

[69] J.M. Campbell, R.K. Ellis, An update on vector boson pair production at hadron colliders. Phys. Rev. D **60**, 113006 (1999). arXiv:hep-ph/9905386 [hep-ph]

[70] J.M. Campbell, R.K. Ellis, C. Williams, Vector boson pair production at the LHC. JHEP **07**, 018 (2011). arXiv:1105.0020 [hep-ph]

[71] ATLAS Collaboration, Monte Carlo generators for the production of a $$W$$ or $$Z/\gamma ^*$$ boson in association with jets at ATLAS in Run 2. ATL-PHYS-PUB-2016-003, 2016. http://cds.cern.ch/record/2120133

[72] S. Catani et al., Vector boson production at hadron colliders: a fully exclusive QCD calculation at NNLO. Phys. Rev. Lett. **103**, 082001 (2009). arXiv:0903.2120 [hep-ph] 10.1103/PhysRevLett.103.082001 19792718

[73] S. Catani, M. Grazzini, An NNLO subtraction formalism in hadron collisions and its application to Higgs boson production at the LHC. Phys. Rev. Lett. **98**, 222002 (2007). arXiv:hep-ph/0703012 [hep-ph] 10.1103/PhysRevLett.98.222002 17677837

[74] ATLAS Collaboration, Vertex reconstruction performance of the ATLAS detector at $$\sqrt{s}$$ = 13 TeV. ATL-PHYS-PUB-2015-026, 2015. http://cds.cern.ch/record/2037717

[75] ATLAS Collaboration, Electron efficiency measurements with the ATLAS detector using the 2012 LHC proton–proton collision data. ATLAS-CONF-2014-032, (2014). https://cds.cern.ch/record/1706245 10.1140/epjc/s10052-017-4756-2 PMC5434979 28579919

[76] ATLAS Collaboration, Muon reconstruction performance of the ATLAS detector in proton–proton collision data at $$\sqrt{s} = 13 TeV$$. Eur. Phys. J. C **76**, 292 (2016). doi:10.1140/epjc/s10052-016-4120-y. arXiv:1603.05598 [hep-ex] 10.1140/epjc/s10052-016-4120-y PMC5321258 28280436

[77] ATLAS Collaboration, Topological cell clustering in the ATLAS calorimeters and its performance in LHC Run 1. (2016). arXiv:1603.02934 [hep-ex] 10.1140/epjc/s10052-017-5004-5 PMC5586976 28943797

[78] M. Cacciari, G.P. Salam, G. Soyez, The anti-k$$_t$$ jet clustering algorithm. JHEP **04**, 063 (2008). doi:10.1088/1126-6708/2008/04/063. arXiv:0802.1189 [hep-ph]

[79] M. Cacciari, G.P. Salam, Dispelling the N$$^{3}$$ myth for the Kt jet-finder. Phys. Lett. B **641**, 57–61 (2006). doi:10.1016/j.physletb.2006.08.037. arXiv:hep-ph/0512210

[80] ATLAS Collaboration, Jet energy measurement and its systematic uncertainty in proton–proton collisions at $$\sqrt{s} = 7 TeV$$ with the ATLAS detector. Eur. Phys. J. C **75**, 17 (2015). doi:10.1140/epjc/s10052-014-3190-y. arXiv:1406.0076 [hep-ex] 10.1140/epjc/s10052-014-3190-y PMC4684939 26709345

[81] ATLAS Collaboration, Jet calibration and systematic uncertainties for jets reconstructed in the ATLAS detector at $$\sqrt{s}=13$$ TeV. ATL-PHYS-PUB-2015-015, 2015. http://cds.cern.ch/record/2037613

[82] ATLAS Collaboration, Tagging and suppression of pileup jets with the ATLAS detector. ATLAS-CONF-2014-018, 2014. http://cds.cern.ch/record/1700870

[83] ATLAS Collaboration, Characterisation and mitigation of beam-induced backgrounds observed in the ATLAS detector during the 2011 proton–proton run. JINST **8**, P07004 (2013). doi:10.1088/1748-0221/8/07/P07004. arXiv:1303.0223 [hep-ex]

[84] ATLAS Collaboration, Selection of jets produced in 13 TeV proton–proton collisions with the ATLAS detector. ATLAS-CONF-2015-029, 2015. http://cds.cern.ch/record/2037702

[85] ATLAS Collaboration, Performance of $$b$$-Jet Identification in the ATLAS experiment. JINST **11**, P04008 (2016). doi:10.1088/1748-0221/11/04/P04008. arXiv:1512.01094 [hep-ex]

[86] ATLAS Collaboration, Optimisation of the ATLAS b-tagging performance for the 2016 LHC Run. ATL-PHYS-PUB-2016-012, 2016. http://cds.cern.ch/record/2160731

[87] ATLAS Collaboration, Electron and photon energy calibration with the ATLAS detector using LHC Run 1 data. Eur. Phys. J. C **74**, 3071 (2014). doi:10.1140/epjc/s10052-014-3071-4. arXiv:1407.5063 [hep-ex]

[88] ATLAS Collaboration, Expected performance of missing transverse momentum reconstruction for the ATLAS detector at $$\sqrt{s}=13$$ TeV. ATL-PHYS-PUB-2015-023, 2015. http://cds.cern.ch/record/2037700

[89] ATLAS Collaboration, Performance of missing transverse momentum reconstruction for the ATLAS detector in the first proton–proton collisions at at $$\sqrt{s}$$ = 13 TeV. ATL-PHYS-PUB-2015-027, 2015. http://cds.cern.ch/record/2037904

[90] G. Cowan et al., Asymptotic formulae for likelihood-based tests of new physics. Eur. Phys. J. C **71**, 1554 (2011). doi:10.1140/epjc/s10052-011-1554-0. arXiv:1007.1727 [physics.data-an]

[91] ATLAS Collaboration, Measurement of the differential cross-section of highly boosted top quarks as a function of their transverse momentum in $$\sqrt{s}$$ = 8 TeV proton–proton collisions using the ATLAS detector. Phys. Rev. D **93**, 032009 (2016). doi:10.1103/PhysRevD.93.032009. arXiv:1510.03818 [hep-ex]

[92] C.M.S. Collaboration, Measurement of the integrated and differential t-tbar production cross sections for high-$$p_{\text{T}}$$ top quarks in pp collisions at $$\sqrt{s}$$ = 8 TeV. Phys. Rev. D **94**, 072002 (2016). doi:10.1103/PhysRevD.94.072002. arXiv:1605.00116 [hep-ex]

[93] M. Czakon, D. Heymes, A. Mitov. Dynamical scales for multi-TeV top-pair production at the LHC. (2016). arXiv:1606.03350 [hep-ph]

[94] ATLAS Collaboration, Search for squarks and gluinos in events with isolated leptons, jets and missing transverse momentum at $$\sqrt{s} = 8 TeV$$ with the ATLAS detector. JHEP **04**, 116 (2015). doi:10.1007/JHEP04(2015)116. arXiv:1501.03555 [hep-ex]

[95] M. Krämer et al., Supersymmetry production cross sections in $$pp$$ collisions at $$\sqrt{s} = 7~TeV$$. (2012). arXiv:1206.2892 [hep-ph]

[96] C. Borschensky et al., Squark and gluino production cross sections in pp collisions at $$\sqrt{s}$$ = 13, 14, 33 and 100 TeV Eur. Phys. J. C **74**, 3174 (2014). doi:10.1140/epjc/s10052-014-3174-y. arXiv:1407.5066 [hep-ph] 10.1140/epjc/s10052-014-3174-y PMC4423871 25983637

[97] M. Baak et al., HistFitter software framework for statistical data analysis Eur. Phys. J. C **75**, 153 (2014). doi:10.1140/epjc/s10052-015-3327-7. arXiv:1410.1280 [hep-ex]

[98] Read A (2002). Presentation of search results: the CLs technique. J. Phys. G Nucl. Part. Phys..

[99] ATLAS Collaboration, ATLAS computing acknowledgements 2016–2017. ATL-GEN-PUB-2016-002, 2016. https://cds.cern.ch/record/2202407

